# Synthesis and Evaluation of Novel Ring‐Strained Noncanonical Amino Acids for Residue‐Specific Bioorthogonal Reactions in Living Cells

**DOI:** 10.1002/chem.202100322

**Published:** 2021-03-04

**Authors:** Christopher D. Reinkemeier, Christine Koehler, Paul F. Sauter, Nataliia V. Shymanska, Cecile Echalier, Anna Rutkowska, David W. Will, Carsten Schultz, Edward A. Lemke

**Affiliations:** ^1^ European Molecular Biology Laboratory Meyerhofstr.1 69117 Heidelberg Germany; ^2^ Biocentre, Departments of Biology and Chemistry Johannes Gutenberg-University Mainz Hanns-Dieter-Hüsch-Weg 17 55128 Mainz Germany; ^3^ Institute of Molecular Biology Ackermannweg 4 55128 Mainz Germany; ^4^ ARAXA Biosciences GmbH Meyerhofstraße 1 69117 Heidelberg Germany; ^5^ Cellzome GmbH GlaxoSmithKline Meyerhofstrasse 1 69117 Heidelberg Germany; ^6^ Department of Chemical Physiology and Biochemistry Oregon Health & Science University (OHSU) Portland Oregon 97239-3098 USA

**Keywords:** click chemistry, kinetics, live-cell labeling, protein engineering, unnatural amino acids

## Abstract

Bioorthogonal reactions are ideally suited to selectively modify proteins in complex environments, even *in vivo*. Kinetics and product stability of these reactions are crucial parameters to evaluate their usefulness for specific applications. Strain promoted inverse electron demand Diels–Alder cycloadditions (SPIEDAC) between tetrazines and strained alkenes or alkynes are particularly popular, as they allow ultrafast labeling inside cells. In combination with genetic code expansion (GCE)‐a method that allows to incorporate noncanonical amino acids (ncAAs) site‐specifically into proteins *in vivo*. These reactions enable residue‐specific fluorophore attachment to proteins in living mammalian cells. Several SPIEDAC capable ncAAs have been presented and studied under diverse conditions, revealing different instabilities ranging from educt decomposition to product loss due to β‐elimination. To identify which compounds yield the best labeling inside living mammalian cells has frequently been difficult. In this study we present a) the synthesis of four new SPIEDAC reactive ncAAs that cannot undergo β‐elimination and b) a fluorescence flow cytometry based FRET‐assay to measure reaction kinetics inside living cells. Our results, which at first sight can be seen conflicting with some other studies, capture GCE‐specific experimental conditions, such as long‐term exposure of the ring‐strained ncAA to living cells, that are not taken into account in other assays.

## Graphical abstract

**How do they click?** A high throughput fluorescence flow cytometry‐based FRET assay enables us to directly measure bioorthogonal reaction kinetics in living cells. Using this assay, we test several new compounds and identify well suited strained dienophiles for genetic code expansion experiments to site‐specifically label proteins *in vivo*.

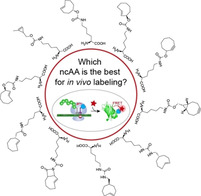

## Introduction

Bioorthogonal reactions are an essential part in the toolbox of chemical biologists that strive to label and modify biomolecules *in vivo*. They can be used to label nucleic acids, sugars, lipids and proteins.^[1]^ For the labeling of proteins, bioorthogonal reactions can be exquisitely combined with genetic code expansion (GCE) technology that enables the co‐translational, site‐specific incorporation of reactive noncanonical amino acids (ncAAs) into proteins.^[2]^ To this end, an orthogonal tRNA/aminoacyl‐tRNA‐synthetase pair is used to suppress a stop codon in the protein of choice.^[3]^ In order for such a pair to be orthogonal to the host, it is crucial that the orthogonal tRNA is not recognized by any endogenous tRNA‐synthetase, while the orthogonal tRNA‐synthetase does not use any endogenous tRNA or any canonical amino acids as a substrate.^[4]^ For eukaryotes, several GCE systems have been described,^[5]^ however the pyrrolysyl‐tRNA/tRNA‐synthetase systems (PylRS/tRNA^Pyl^) derived from methanogenic archaea arguably represent the most versatile and efficient ones, and therefore most commonly used GCE tools in eukaryotes.^[6]^ These PylRS systems have been used to encode a plethora of ncAAs, compatible with several types of bioorthogonal reactions into proteins in living cells. So far, the fastest, and one of the most potent bioorthogonal reactions that has been encoded for *in vivo* labeling is the strain‐promoted inverse electron demand Diels–Alder cycloaddition (SPIEDAC).^[7]^ Commonly a tetrazine is used as the diene component, which readily reacts with a strained dienophile like *trans*‐cyclooctene or cyclooctyne.[^2b^, ^2c^, ^2d^, ^2e^] Several ncAAs with such ring strained side chains have been genetically encoded using the PylRS/tRNA^Pyl^ system, these include some examples of tyrosine derivatives^[8]^ and various lysine derivatives, including equatorial *trans*‐cyclooct‐4‐ene‐ (**1**, TCO‐E), axial *trans*‐cyclooct‐2‐ene‐ (**2**, TCO*‐A), cyclooctyne‐ (**3**, SCO, note that this is a special case as it likely does not proceed with an inverse electron demand^[9]^) and *endo* bicyclononyne‐lysine (**4**, BCN).[^2b^, ^2c^, ^2d^, ^2e^]

For live cell labeling experiments, an ideal compound should have a fast reaction rate, it should be stable prior to the conjugation reaction and the reaction product should remain stable. Therefore, it is of high interest to accurately measure these reaction parameters in order to choose the best possible ncAA. Kinetic parameters of the head group, the amino acid or a purified protein have often been measured *in vitro* and sometimes requiring even non‐physiological buffers due to solubility issues.[^7a^, ^10^] SPIEDAC reactions have also been evaluated using enzymatic methods to attach a reactive group to a protein *in vivo* which is then subsequently modified with the corresponding bioorthogonal reactive group.[^2e^, ^11^] A recent study found the head group of TCO*‐A to be less reactive than the head group of BCN.^[11d]^ At first sight this seems to conflict with the observation made by us and others that in GCE based experiments TCO*‐A outperforms BCN and yields the highest contrast for fluorescence imaging, indicating most stable product formation.^[12]^ However, our observation in itself might already be counterintuitive, as the TCO*‐A/tetrazine SPIEDAC reaction products can undergo β‐elimination,^[13]^ in which the fluorophore would be lost. The efficiency of this elimination process depends on the solvent and on the tetrazine substituents^[14]^ and it is clearly necessary to take it into account for developing quantitative fluorescent labeling strategies in cells. These conflicting data stress the importance of evaluating bioorthogonal reactions at application specific conditions, and that kinetic and stability information cannot always easily be transferred between different assays. This is particularly relevant, for optimizing the labeling parameters in the complex environment of a cell, as it is typically impossible to fully control all reaction parameters without interfering with the normal physiology of the host.

While most commonly the dienophile is genetically encoded into proteins, it is also possible to genetically encode the tetrazine.^[15]^ This technology was recently extended to mammalian cells and it was shown that the genetically encoded tetrazine can react fast with a particularly strained dienophile.^[7c]^ For fluorescence imaging based studies, this however comes with a limitation. Dyes often tend to stick to various parts of the cell and therefore fluorogenicity is an important feature to enable better signal to noise ratios for *in vivo* applications.^[16]^ For tetrazine based SPIEDAC reactions it has been shown that the tetrazine itself can serve as a quenching moiety for the organic fluorophore and thus the reaction with the dienophile leads to a substantial fluorescence increase.^[17]^ Therefore, it is advantageous to genetically encode the dienophile and use the tetrazine coupled to a dye as the fluorogenic probe. It is thus of high interest to optimize the stability and reactivity of the genetically encoded dienophile in order to enable the access to the reported repertoire of fluorogenic tetrazine derivatives.^[17]^

**Figure 1:**
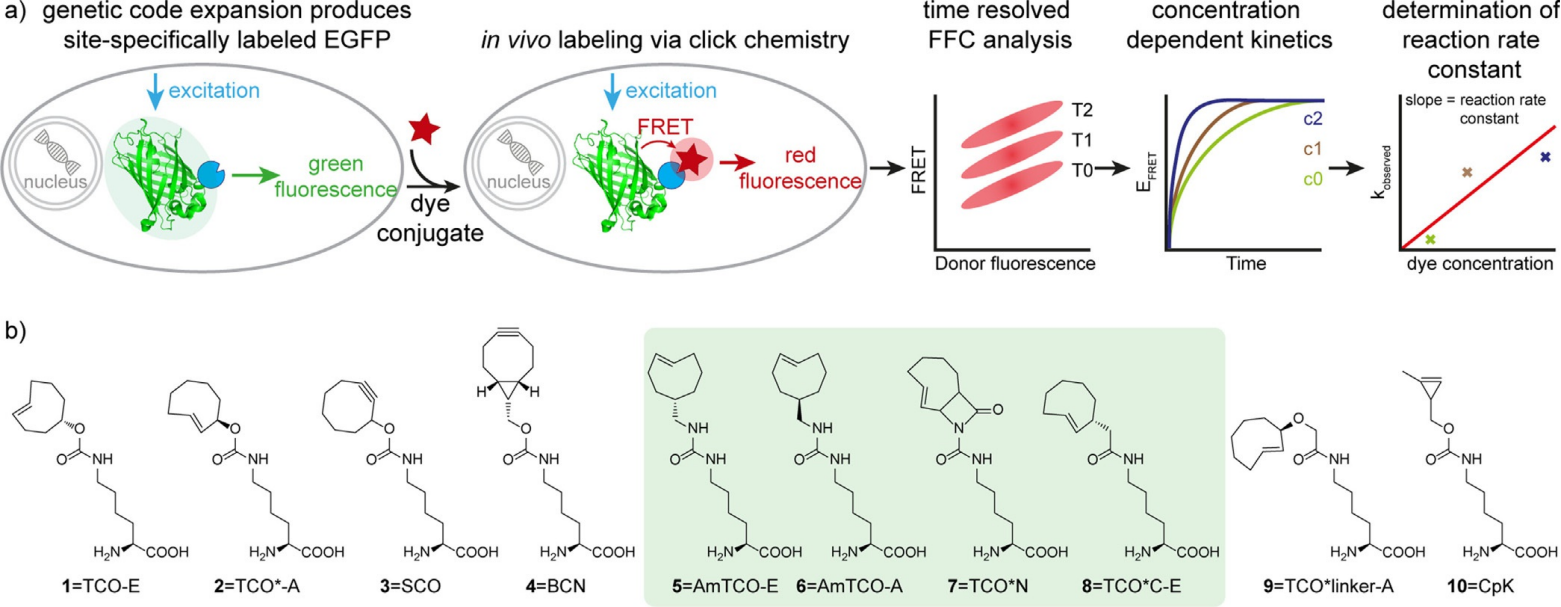
*In cellulo* FRET measurements to obtain kinetics inside living mammalian cells. a) EGFP with a site‐specifically incorporated ncAA is expressed in HEK293T cells. A tetrazine‐dye conjugate is added at defined concentrations and FRET from EGFP to the dye is measured in a time resolved fashion (EGFP structure PDB 2y0g^[18]^). The relative E_FRET_ is calculated for every time point and plotted against the time. From these data an observed reaction rate constant (*k*_Obs_ is calculated for each concentration and plotted against the dye concentration. Finally, a pseudo first‐order reaction rate constant (*k*_On_) is calculated from these data. b) Chemical structures of ncAAs analyzed in this work. Newly synthesized ncAAs are highlighted with a green background.

In this paper we present multiple advancements towards this goal. i) We designed and synthesized four conceptually novel ncAAs which cannot lose their fluorescent dye conjugate due to β‐elimination. ii) We developed a simple, high‐throughput FRET‐based assay to quantitatively measure relative reaction kinetics of genetically encoded bioorthogonal handles directly in living cells using fluorescence flow cytometry (FFC), see Figure 1 for a graphical overview. This assay enables the faithful evaluation of labeling reactions under the actual *in vivo* GCE conditions, simultaneously evaluating compound stability, reaction rate and product stability, and thereby the assay enables the simple identification of the best ncAA for labeling in living mammalian cells.

## Results and Discussion

As we found TCO*‐A so far to yield the highest contrast imaging in eukaryotic cells,[^10a^, ^12a^] we aimed to design ncAAs that maintain the positive features of this compound (high reactivity) but less prone to losing their payload after conjugation.

**Scheme 1:**
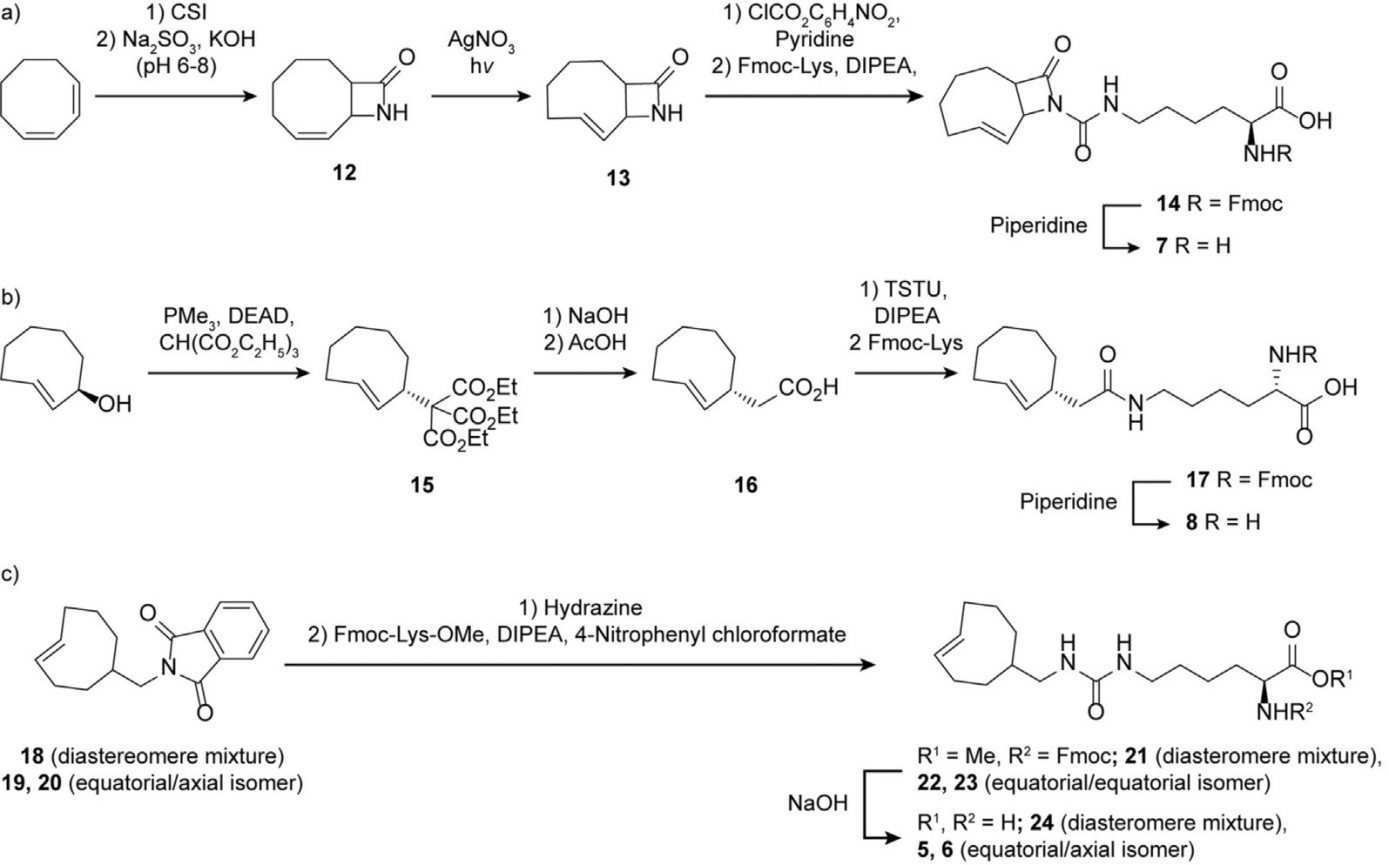
Synthesis of TCO* derivatives. a) Synthesis of TCO*N, b) synthesis of TCO*C‐E and c) synthesis of AmTCO‐A and AmTCO‐E. The synthesis of 18–20 was described before.^[19]^

To this end, we synthesized axial and equatorial aminomethyl‐*trans*‐cyclooct‐2‐ene‐lysine (**5**, AmTCO‐E and **6**, AmTCO‐A) based on the recently reported highly reactive AmTCO head group.^[19]^ In addition, we synthesized, lactame‐*trans*‐cyclooct‐2‐ene‐lysine (**7**, TCO*N) and equatorial *trans*‐cyclooct‐2‐ene acetic acid (**8**, TCO*C‐E). The synthesis procedure for the four new ncAAs is shown in Scheme 1; for more details see Supplementary Methods. *Trans*‐cyclooctene moieties are usually synthesized as alcohol derivatives, i.e. *trans*‐cyclooctenols and then connected as a carbamate to the amino acid backbone. In brief, AmTCO‐E and AmTCO‐A instead are connected to the lysine residue via a urea functionality, based on the hypothesis that a different linkable group on the *trans*‐cyclooctene moiety could help tuning the properties of the resulting conjugates. In TCO*C‐E the *trans*‐cyclooctene moiety is attached to the lysine backbone via an amide bond instead of the carbamate found in TCO*‐A.

These three amino acids lack the carbamate in β position to the double bond and should be less susceptible to undergoing β‐elimination. For TCO*N a different strategy was envisioned to suppress payload loss. Here β‐elimination can still occur, but due to the lactam bond in the 3‐position the payload would still remain attached to the protein.

In addition to the already mentioned ncAAs, we also tested methylene‐*trans*‐cyclooct‐2‐ene‐ (**9**, TCO*linker‐A) and cyclo‐propene‐lysine (**10**, CpK).^[20]^

In GCE experiments the ncAA bearing the reactive group is incorporated into the protein of interest during translation. This process is very slow and hence the reactive group is exposed to the cellular environment for a much longer period of time, as compared to for example other SPIEDAC reactive assays where the compounds are only added for a short time to the cell.^[11a]^ We thus aimed to develop an assay that allows to directly assess the reaction speed and product stability in living cells.

The first step for our assay development was to determine appropriate ncAA concentrations to obtain similar amber suppression levels by titrating the ncAAs in transient transfection experiments (Figure S1). Then we expressed EGFP^Y39TAG^ with the respective ncAA in HEK293T cells, washed out excess ncAA and labeled EGFP^Y39ncAA^ with three different concentrations of a silicone rhodamine tetrazine conjugate (SiR‐tet). We aimed for a comparatively low EGFP expression level to avoid aggregation or accumulation of EGFP in the cell as well as ensuring a pseudo‐first‐order reaction with the tetrazine group. The position of the amber stop codon at Y39 in the EGFP protein was selected to ensure that the fluorophore of EGFP and the attached fluorophore of the tetrazine–dye conjugate are in close proximity to enable efficient FRET from EGFP to the dye. We took samples after appropriate time points and measured EGFP fluorescence as well as the resulting FRET signal via FFC. We subsequently calculated the relative FRET efficiency (*E*_FRET_) for each dye concentration over time, determined the observed reaction rate constant (*k*_Obs_), and used this concentration dependent value to calculate the pseudo‐first‐order rate constant *k*_On_ (Figure 1). Additionally, we also calculated the maximal reached value of *E*_FRET_, which we term relative *E*_FRET‐MAX_, namely the highest *E*_FRET_ signal at the highest dye concentration for each ncAA. Assuming that the FRET efficiency and quantum efficiency of the fluorophores used is initially constant across all experimental conditions, this value can be seen as a relative estimate of the educt stability after expression, that is, how much reactive ncAA was present at the start of the labeling experiment. We also observed the *E*_FRET_ over an extended period of time (>3 h). In some cases the reaction curves reached a temporary maximum and afterwards a slight drop in *E*_FRET_ occurred. This is indicative of decaging or formation of a nonfluorescent species over time. To estimate this effect, we also report *E*_FRET‐Final_, that is, the measured *E*_FRET_ after 5 h of measurement, which can be seen as a relative parameter to compare fluorescent product stabilities.

**Figure 2:**
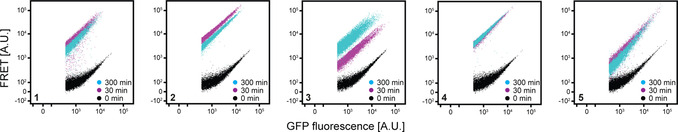
*In cellulo* FRET based kinetic measurements to evaluate bioorthogonal reactions *in vivo*. HEK293T transiently expressing PylRS, tRNA^Pyl^ and EGFP^Y39ncAA^ (with either of the ncAAs 1–5) were labeled with 1 μm SiR‐tet and analyzed via FFC after indicated time points. The FFC plots show overlays of the GFP fluorescence vs. FRET signal of cells for each ncAA before labeling (0 min, black) as well as 30 (magenta) and 300 minutes (cyan) after addition of SiR‐tet. For this analysis only cells above a GFP threshold of 4*×*10^2^ A.U. are taken into account (each dot shows the fluorescence/FRET values of a single cell).

**Figure 3:**
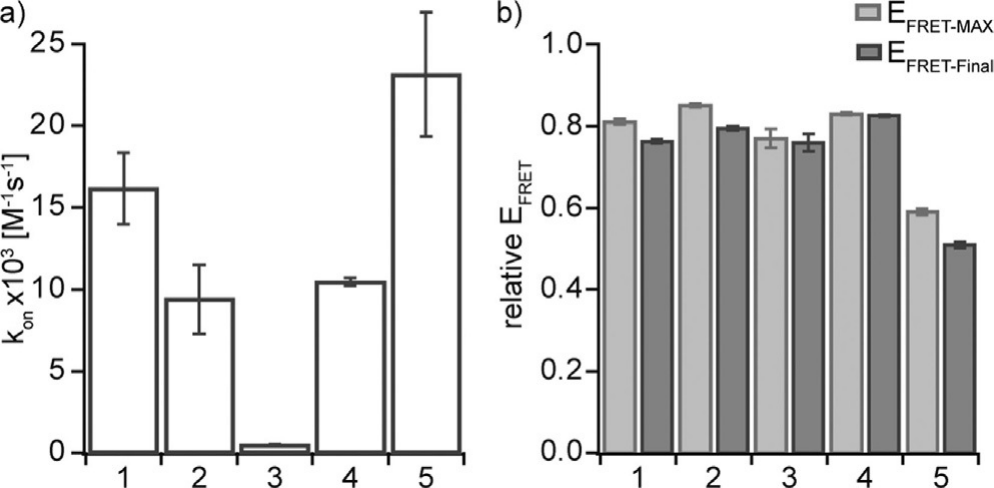
Kinetic parameters of SPIEDAC reactions *in cellulo*. a) Bar graph showing the measured *in cellulo* reaction rates for the ncAAs. Shown are the mean values with SEM for at least three independent experiments. b) Bar graph showing the measured relative *E*_FRET‐MAX_ values for the ncAAs as well as the *E*_FRET‐Final_ (relative *E*_FRET_ after 5 hours). Shown are the mean values with SEM for at least three independent experiments.

Using our new *in cellulo* FRET assay, we observed both rapid and highly efficient labeling for TCO‐E, TCO*‐A, BCN, AmTCO‐E and TCO*linker‐A (Figure 2, Figures 3, S2 and S3). The fastest of these is AmTCO‐E with *k*_On_ above 20 000 m ^−1^ s^−1^, however it only reaches an *E*_FRET‐MAX_ of less than 0.6, its axial isomer AmTCO‐A reacts similarly fast but reaches an even lower *E*_FRET‐MAX_ of merely 0.2. The low relative *E*_FRET‐MAX_ for these two compounds is likely caused by instability of the ncAA prior to reaction. The ncAA with second highest *k*_On_ of about 15 000 m ^−1^ s^−1^ is TCO‐E, which also reaches a high relative *E*_FRET‐MAX_ of 0.8. BCN and TCO*‐A react slower and both exhibit a reaction rate constant of about 10 000 m ^−1^ s^−1^, but both also reach a relative *E*_FRET‐MAX_ of 0.8. TCO*linker‐A is even a bit slower with a *k*_On_ of approximately 6000 m ^−1^ s^−1^, but it also reaches a relative *E*_FRET‐MAX_ of 0.8. TCO*N and TCO*C‐E also reach high relative *E*_FRET‐MAX_ values of 0.8 but they react comparatively slow with a *k*_On_ below 2000 m ^−1^ s^−1^. Expectedly, the reaction proceeds even slower for SCO and CpK (Figures 2, 3 and S3).

Within 5 hours TCO*‐A, and TCO‐E loose about 7 % relative *E*_FRET_, TCO*linker‐A looses 10 %, while AmTCO‐E looses about 15 % (Figures 2, 3 and S3). For SCO and BCN we observe no substantial loss of relative *E*_FRET_ during the entire measurement window. In the case of TCO*‐A this is most likely caused by β‐elimination as reported previously.^[13]^ As TCO‐E and AmTCO‐E cannot undergo ß‐elimination, the signal loss can potentially be attributed to another form of product instability or an alternative dye quenching mechanism.^[21]^ In the case of AmTCO‐E, the reaction at 37 °C was too fast to faithfully calculate *k*_Obs_. To overcome this bottleneck, we measured TCO‐E, AmTCO‐A and TCO*‐A also on ice (Figure S4). Here, AmTCO‐E also showed faster labeling rates than TCO*‐A and TCO‐E, but did not reach the same relative *E*_FRET‐MAX_. Overall, these data reveal that, while our new compounds showed partially better characteristics then TCO*‐A or BCN, in total they are not better choices for GCE based fluorescence labeling than these two.

Already in preliminary screening experiments, we discovered one previously hardly used compound TCO‐E which reacted even faster than TCO*‐A. However, TCO‐E was only poorly accepted by the known PylRS mutants, and thus yields very low incorporation efficiency explaining why this compound was rarely used. To increase the incorporation efficiency of TCO‐E, we performed a synthetase library screening. Therefore, five positions in the binding pocket of PylRS^AF^ were mutated to any of the 20 amino acids at these positions. The library was selected via chloramphenicol resistance (positive selection) and barnase expression (negative selection).^[22]^ The resulting PylRS variant (PylRS^AF‐A1^) containing the mutations Y306A, L309M, C348G, Y384F and I405R showed up to five‐fold higher incorporation efficiency (Figure S5). This new mutant also showed better performance for TCO‐E incorporation and was used for all experiments shown, demonstrating that in fact TCO‐E reacts substantially faster than TCO*‐A and also reaches a comparable relative *E*_FRET‐MAX_ (Figures 2, 3). Interestingly, the corresponding axial isomer (TCO‐A) is completely unreactive in mammalian cells and when testing proteins purified from *E. coli* (Figure S6). This seems to contrasts the previously reported higher reactivity of the axial TCO isomer compared to the equatorial isomer.^[11e]^

To better understand the origin of some of those discrepancies, we also performed a set of *in vitro* experiments. To this end, we measured reaction kinetics *in vitro* using EGFP, with site‐specifically inserted ncAAs purified from *E. coli*.

To measure the kinetics we labeled the protein with a 3‐(*p*‐benzylamino)‐1,2,4,5‐tetrazine‐Cy5 (Cy5‐tet) conjugate and followed the increasing FRET from EGFP to Cy5. In good agreement with the *in cellulo* data TCO*‐A reacts rapidly and almost quantitatively, while SCO reacts slow but also quantitatively. Interestingly, BCN shows hardly any labeling *in vitro*, which indicates that this compound decomposes almost completely under these experimental conditions. *In vitro*, TCO‐E shows lower *E*_FRET_ than TCO*‐A, which can potentially be attributed to some extend to *trans*–*cis* isomerization of the double bond (Figure S7). The isomer TCO‐A is even less reactive, indicating a further destabilization of the double bond in this isomer (Figure S6).

We confirmed that TCO‐E and TCO‐A both are highly reactive as free amino acids by performing an LC‐MS based measurement (solvent 50 % acetonitrile in water, Figure S8). Intriguingly, once the pure amino acids are incubated in LB medium or *E. coli* cultures they quickly isomerize. Particularly, in *E. coli* cultures grown in LB medium for 12 hours, 95 % of TCO‐A isomerized from *trans* to *cis*, while of TCO‐E only 72 % and of TCO*‐A merely 33 % were in *cis*‐conformation. In the same time, 78 % of BCN decomposed in *E. coli* cultures (Figure S9).

## Conclusions

In this work we developed four novel ncAAs that after click labeling with a tetrazine should not be able to loose their payload by β‐elimination. We then developed a simple assay to quantitatively compare the compounds for *in cellulo* labeling experiments. We demonstrate that this assay can be executed at different biologically relevant temperatures and it should thus be possible to adapt this assay to any host system that is amenable to FFC analysis.

With this assay we identified that our four new ncAAs can undergo SPIEDAC reactions in living cells, we can confirm fast labeling reactions for TCO*‐A and BCN and we rediscover TCO‐E, a previously largely ignored ncAA, to exhibit very fast kinetics as well as high educt and product stability in mammalian cells. We also note that the synthesis of TCO*N turned out to be impractical to yield the large amounts necessary for large scale protein expression experiments.

Strikingly, we observed that kinetics and stability measured *in cellulo* can be different from those measured *in vitro*, highlighting that it is necessary to evaluate bioorthogonal reactions under the relevant conditions of the host system. Therefore, we hope that our new assay will find broad application for further ncAA optimization.

## Conflict of interest

The authors declare no conflict of interest.

## Supporting information

As a service to our authors and readers, this journal provides supporting information supplied by the authors. Such materials are peer reviewed and may be re‐organized for online delivery, but are not copy‐edited or typeset. Technical support issues arising from supporting information (other than missing files) should be addressed to the authors.

Supplementary
