# Supplementary material for: Synthesis and Evaluation of Novel Ring‐Strained Noncanonical Amino Acids for Residue‐Specific Bioorthogonal Reactions in Living Cells
Source: Chemistry. 2021 Mar 4;27(19):6094–9. doi: 10.1002/chem.202100322 (PMC8049044; doi:10.1002/chem.202100322)
Supplement: Supplementary file 1 — Supplementary [file CHEM-27-6094-s001.pdf]

Chemistry–A European Journal

Supporting Information

Synthesis and Evaluation of Novel Ring-Strained Noncanonical Amino Acids for Residue-Specific Bioorthogonal Reactions in Living Cells

Christopher D. Reinkemeier+,[a, b, c] Christine Koehler+,[a, b, c, d] Paul F. Sauter,[a, d] Nataliia V. Shymanska,[a] Cecile Echalier,[a] Anna Rutkowska,[e] David W. Will,[a] Carsten Schultz,[a, f] and Edward A. Lemke*[a, b, c]

Author Contributions

C.R. Conceptualization: Equal; Investigation: Equal; Writing – original draft: Equal; Writing – review & editing: Equal
C.K. Conceptualization: Equal; Investigation: Equal; Writing – original draft: Equal; Writing – review & editing: Equal.

Contents

Experimental details

Cell culture

Cloning of constructs

E. coli expression and protein purification

In vitro kinetic studies

Measurement of the reactivity and stability of the free ncAA

Estimation of incorporation efficiency of different ncAAs

In vivo kinetic measurements (*in cellulo* FRET)

Evolution of PylRS synthetase for incorporation of TCO-E

Synthetic procedures

Synthesis of TCO*N

Synthesis of TCO*C-E

Synthesis of Am-TCO

Supporting figures

Figure S1. Comparison of the incorporation efficiency of different ncAA

Figure S2. *In cellulo* FRET FFC data, kinetics and reaction rate constant determination of TCO*-A, TCO-E, BCN, SCO and AmTCO-E

Figure S3. *In cellulo* FRET FFC data, kinetics and reaction rate constant determination for TCO*N, TCO*C-E, TCO*linker-A, CpK and AmTCO-A

Figure S4. *In cellulo* FRET on ice for TCO*-A, TCO-E and AmTCO-E

Figure S5. Results of the evolution of PylRS synthetase

Figure S6. TCO-A *in vitro* reactivity (sfGFP purified)

Figure S7. *In vitro* FRET studies of recombinant EGFP^{Y39ncAA}

Figure S8. LC-MS analysis of free ncAAs

Figure S9. IED-MS analysis of ncAAs in LB medium and *E. coli* cultures

Supporting references

Experimental details

Cell culture

HEK293T cells (ATCC CRL-3216) were cultivated in Dulbecco's modified Eagle's medium (DMEM, Gibco 41965-039) supplemented with 10% FBS (Sigma-Aldrich F7524), 1% penicillin-streptomycin (Sigma-Aldrich P0781), 1% L-Glutamine (Sigma-Aldrich G7513) and 1% sodium pyruvate (Life Technologies 11360). Cells were incubated at 37 °C and 5% CO_2, split every 2-3 days up to passage 20. For transient transfections, HEK293T cells were seeded in a 24-well plate (Nunclon Delta Surface ThermoFisher SCIENTIFIC) at a cell density of 220.000 cells/ml, 500 µl/well. The seeding density in the case of 60 mm petri dishes was 500.000 cells/ml, 3 ml per dish. Cells were seeded 16 hours prior to transfection. For transfection, Dulbecco's modified Eagle's medium without Phenol-Red (DMEM, Gibco 11880-028) and as transfection reagent polyethylenimine (PEI, Sigma 408727) at a concentration of 1 mg/ml was used.

Cloning of constructs

For the expression in mammalian cells, EGFP was cloned with an N-terminal FLAG tag and a C-terminal 6xHis-tag into the commercial pCI plasmid (Promega, E1731), containing an amber stop codon at position Y39, resulting in the plasmid pCI-FLAG-EGFP^{Y39TAG}-6His. As a second reporter plasmid, we are using the pCI-iRFP-EGFP^{Y39TAG}-6His plasmid, as published earlier by us[1]. Plasmid pCMV-NES-PylRS^{AF}-U6tRNArv is carrying the PylRS tRNA-synthetase from *Methanosarcina mazei* (Mm PylRS^{AF}), containing the Y306A and the Y384F mutation respectively and an N-terminal NES signal, as well as the tRNA expression cassette in an opposite direction, consisting of an U6 promoter signal and the *Methanosarcina mazei* tRNA^{Pyl} gene.

For the *in vitro* studies, we used the construct pALS-Flag-EGFP^{Y39TAG}-6His, which we obtained by cloning FLAG-EGFP^{Y39TAG}-6His into the plasmid pALS-sfGFP^{N150TAG}-MbPyl-tRNA replacing the sfGFP gene. For the expression of the synthetase, we cloned the different PylRS genes into the pBK plasmid[2], a generous gift from Ryan Mehl. First, we cloned Mm PylRS^{WT} into the pBK plasmid, using the restriction sites NdeI and PstI, after introducing a BglII site into the PylRS gene at bps 870-875, resulting into pBK-PylRS^{WT} plasmid, containing a Kanamycin resistance cassette (Kan). The BglII site was further used together with PstI to clone the specific binding pocket of the Mm PylRS^{AF} mutant (pBK-PylRS^{AF}) and Mm PylRS$^{AF\text{-}A1}$ mutant into the pBK plasmid (pBK-PylRS$^{AF\text{-}A1}$), by replacing this part of the PylRS^{WT} synthetase.

Plasmid used for the synthetase evolution, pREP-PylT, pYOBB2-PylT and pALS-sfGFP^{N150TAG}-MbPyl-tRNA were generous gifts from the Ryan Mehl lab[2-3].

The PylRS synthetase variant AF-A1 was cloned out of the pBK-PylRS$^{AF\text{-}A1}$ plasmid extracted from the synthetase selection procedure and after digesting with BglII and PstI inserted into pCMV-NES-PylRS^{AF}-U6tRNArv, replacing the corresponding nucleotides of PylRS^{AF}.

E. coli expression and protein purification

DH10B cells were transfected with both the pALS-Flag-EGFP^{Y39TAG}-6His and the required pBK-PylRS plasmid (PylRS^{AF} or PylRS$^{AF\text{-}A1}$). 2 ng of each plasmid was used together with 50 µl of electrocompetent DH10B cells. The electroshock was performed in 1 mm cuvettes using a Bio-Rad Gene Pulser Xcell™ Electroporation Systems. Directly after the electroshock 200 µl of SOC medium were added to the cells. After incubation at 37 °C shaking at 800 rpm for at least 30 minutes, the cells were plated on LB-agar plates containing Tetracycline (Tet, 12.5 µg/ml) and Kanamycin (Kan, 50 µg/ml) as resistance markers. The plates were incubating at 37 °C for at least 16 hours. For the expression, one single colony was incubated overnight shaking at 37 °C in 5 ml LB medium containing the appropriate antibiotics. Next day, 500 µl of this pre-culture were used to inoculate 250-500 ml of LB medium with Tet and Kan and the cultures were incubated again shaking at 37 °C. When the cultures reach an OD_{600} of 0.2-0-4, the noncanonical amino acids were added in a final concentration of 1 mM. Therefore, each non-canonical amino acid was dissolved in 0.1 mM NaOH to 100 mM stock concentration. At OD_{600} of 0.4-0.6, the protein expression was induced, using 0.02% Arabinose. The cultures were incubated for another 16 hours, harvested and pellets stored at -20 °C if not directly used for purification.

For the expression of sfGFP^{N150TAG}, the plasmid pALS-sfGFP^{N150TAG}-MbPyl-tRNA was co-transformed with pBK-PylRS$^{AF\text{-}A1}$ into DH10B cells, following the same procedure as above. The expression was carried out the same way as for the EGFP expression.

For the purification cells were resuspended in 2.5-5 ml of 4xPBS, 0.2 mM TCEP, 1 mM PMSF and 5 mM Imidazole pH 8 (lysis buffer) and brought over into 15 ml falcon tubes. After sonication for 30 seconds on ice, cells were centrifuged at 25000 rcf at 4 °C for 30 minutes. The supernatant was incubated in 15 ml Eppendorf tubes on 250-500 µl nickel beads, equilibrated in lysis buffer, for 1-2 hours at 4 °C on a rocker, packed in aluminum foil. The solution was poured into PD10 columns and tubes were cleaned out with 5 ml lysis buffer. Nickel beads were washed with 10 mM Imidazole in lysis buffer for 50 ml and protein was eluted in 5 ml of 4xPBS, 0.2 mM TCEP, 1 mM PMSF and 500 mM Imidazole pH 8. To further purify the samples, a size-

exclusion column was used (Superdex S200 Increase 10/300, GE Healthcare), equilibrated in 1xPBS pH 8. Fractions were analyzed by SDS-PAGE and pure proteins were concentrated in an Amicon filter device (3 kDa cutoff, Merck Millipore).

In vitro kinetic studies

Concentrations of purified EGFP^{Y39ncAA} were measured by Absorbance scan (Duetta - Fluorescence and Absorbance Spectrometer, HORIBA) and 10 µM stock solutions of each protein sample was prepared. Kinetic measurements were done with 100 nM of protein in a 1 mm quartz cuvette, diluting the 10 µM stock 1:100 in 1xPBS. The zero point was measured using a fluorescent scan, exciting at 450 nm and measuring emission form 470-700 nm (Duetta, Horiba). Then Cy5-H-tetrazine (Cy5-tet) was added into the cuvette, to obtain a final concentration of 1 µM of dye. The resulting FRET signal was measured with the same fluorescent scan as before, measuring every 5 seconds for 600 seconds long, in the case of the EGFP$^{Y39TCO*-A}$ and EGFP$^{Y39TCO-E}$. For EGFP^{Y39SCO} and EGFP^{Y39BCN}, the measuring time was elongated to 120 minutes, measured every 60 seconds. For the 10-hour measurement, the data were recorded every 5 minutes. For each *in vitro* kinetics analysis, 120 single measurements were taken and the maximum value at 500-520 nm and 660-680 nm emission was extracted out of the data to calculate the E_{FRET} value:

$$E_{FRET,} = \frac{Max\,(660-680\,nm)}{(Max\,(660-680\,nm) + Max(500-520\,nm))}$$

Measurement of the reactivity and stability of the free ncAA

To confirm reactivity of ncAAs they were first analyzed via LC-MS to identify when they elute from the column. Subsequently, equimolar amounts of 1 mM ncAA in MeCN and 1 mM 3,6-dimethyl-1,2,4,5-tetrazine (dimethyl-tetrazine) were mixed and analyzed after five minutes via LC-MS.

ncAA stability was either analyzed directly in LB medium by incubating it at a concentration of 1 mM at 37 °C, shaking at 180 rpm followed by LC-MS analysis at appropriate time points or directly in bacterial cultures. To this end, an overnight culture of *E. coli* Top10 cells was diluted to an OD600 of 0.1. For the experiment 5 ml cultures in 14 ml test tubes were set up with a concentration of the respective ncAA of 1 mmol/l or without any ncAA as a control. 500 µl samples were taken at the appropriate time points and cells were lysed via sonication on ice in a Bandelin Sonopuls for 2 times 20 sec at 40% power. Samples were spun down (25000 rcf) and the supernatant analyzed via LC-MS. For identification of the ncAAs the extracted ion count (EIC) of the respective mass of the used ncAA was extracted from the obtained mass spectra.

LC-MS data were obtained on an Agilent 1260 Infinity / Infinity II equipped with an analytical column (Macherey-Nagel EC 250/4 Nucleodur 100-5 C18ec).

Estimation of incorporation efficiency of different ncAAs

To analyze the incorporation efficiency for each ncAA used in the in-cell FRET studies, we performed transient transfection with different amounts of ncAAs and followed the EGFP signal after 24 hours post transfection. Therefore, HEK293T cells were seeded in a 24-well plate 16 hours prior to transfection. For the two-plasmid transfection (pCI-FLAG-iRFP-25Helix-EGFP^{Y39TAG}-6His and pCMV-NES-PylRS^{AF}-U6tRNArv), 1 µg of total DNA was used per well. The DNA was mixed with 50 µl of DMEM medium without Phenol Red and 3 µl of PEI were added. After 10 seconds of vortexing and a short centrifugation step, the DNA mixture was incubated 15 minutes in the hood, before it was added drop-wise to the well. A master mix was prepared for all wells whenever suitable. After four hours of incubation, the medium was aspirated and fresh medium with different concentrations of ncAAs was added. The concentration of ncAA was ranging from 2 µM up to 500 µM. After 20 hours of incubation, the EGFP signal was analyzed with a flow cytometer analyzer (LSRFortessa™, BD Biosciences) using the green laser with a 530-30 filter. Stock solutions for all ncAAs were prepared as described previously[4], ncAAs not synthesized by us were purchased from SiChem (SIRIUS FINE CHEMICALS, SICHEM GMBH, Germany). N$^{\varepsilon}$-tert-butyloxycarbonyl-L-lysine (Boc, **11**) was purchased from Iris Biotech GmbH, Germany)

In vivo kinetic measurements (*in cellulo* FRET)

For each ncAA to be tested, three 60 mm petri dishes were seeded 16 hours prior to transfection with 500.000 cells/ml, 3 ml per dish, as mentioned above. In addition, two rows in a 24-well plate were seeded for control experiments. In the morning, cells were transfected with pCI-FLAG-EGFP^{Y39TAG}-6His and pCMV-NES-PylRS^{AF}-U6tRNArv in a 1:1 ratio, using 17 µg of total DNA per 60 mm petri dish. A master mix for three 60 mm petri dishes was set up by using 53 µg total DNA mixed with 1500 µl DMEM medium without Phenol Red and 153 µl PEI. The master mix was vortexed for 10 seconds, centrifuged down for 4-5 seconds and incubated at room temperature (RT) for 15 minutes. The master mix was distributed equally throughout the three 60 mm petri dishes. For the control plate, the master mix contained 6 µg total DNA, 600 µl of medium and 36 µl of PEI, was vortexed for 10 seconds, spun down and also incubated for 15 minutes at RT before it was pipetted drop-wise into 12-wells. The cells were incubated in the incubator for at least 4 hours, then the medium was changed and the appropriate amount of each ncAA was added to the medium. Cells were further incubated for 18 hours in the incubator. Three hours before

the *in vivo* kinetic measurements, the washing procedures was started. Therefore, the medium was aspirated from the petri dishes and the 24-well plate and replaced by fresh medium without any ncAAs. After 30 minutes in the incubator, the cells were washed with DMEM without Phenol Red and incubated another 30 minutes, this washing step was repeated another time and the cells were stored for another hour in the incubator.

An aliquot of silicon rhodamine 4-(1,2,4,5-tetrazin-3-yl)benzylamino (SiR-tet, Spirochrome) was dissolved in DMSO and concentration was measured in 1xPBS, 0.1 % SDS solution (extinction factor 100.000 mol^{-1} * cm^{-1} at λ_{abs} at 652 nm)[5].

The kinetic measurements were performed on a flow cytometry analyzer (LSRFortessa™, BD Biosciences) using the green laser (488 nm) with a 530/30 filter to monitor EGFP signal and a 710/50 filter to capture the FRET signal. The measurements were started with aspirating the medium of the three 60 mm petri dishes from the same ncAA. The cells from each 60 mm petri dish were resuspended in 1.2 ml of the same DMEM medium without Phenol Red, filtered and transferred into test tubes suitable for the LSRFortessa. 1 ml of these cells was used for the labeling reaction, while the rest was used to measure the zero-time point. Three different kinds of SiR-tet concentrations were used for the measurements, ranging from 100 nM to 1 µM of dye. The appropriate amount of dye was mixed with the cells, vortexed briefly and incubated in a cell culture incubator at 37 °C, 5% CO_2. The labeling reactions were set up in a 2-minute time interval, to give enough time to measure all three concentrations at a specific time point. Measurements were done after 5, 10, 30, 60, 180, 240 and 300 minutes. If the reaction between the ncAA and the tetrazine was very low, a 360-minute time point was also included as well. After 1 hour and 6 minutes a second batch of cells with another ncAA was labeled with the dye and the measurement was started.

For the measurements on ice, cells were cooled down on ice before labeling and kept on ice after labelling as well as throughout the whole measurements.

To analyze the $E_{FRET\text{-}MAX}$ and the k_{on} for each ncAA-tetrazine reaction, data was evaluated with FlowJo™ (Becton, Dickinson & Company). Cells were gated for live cells (FSC vs SSC), followed by single cells gating (FSC vs SSW). After gating for EGFP positive cells, the mean values for the 530 nm and 710 nm signal were extracted, out of which the E_{FRET} for each time point can be calculated as follows:

$$E_{FRET} = \frac{mean(FRET\ signal)}{mean(FRET\ signal) + mean(GFP\ fluorescence)}$$

The resulting E_{FRET} values were corrected with the E_{FRET} value at time point zero. The data was plotted in Igor and fitted with an exponential fit:

$$f(x) = y_0 + Ae^{(-invTau \cdot x)}$$

from which the k_{Obs} for each concentration curve could be obtained. Plotting the three k_{Obs} of each ncAA versus the concentrations used and fitting these data points with a linear fit:

$$f(x) = a + bx$$

one can obtain the k_{On} of the reaction between this ncAA and the tetrazine, which is the incline of the linear fit.

We note that the concentration of SiR-tet given is the concentration pipetted in the medium. For the reaction to take place, the dye also has to cross the membrane to get into the cell. All those steps are absorbed into the reaction rate constant we observe, as opposed to fitting with more complex models, as other than the dye concentration all other concentration were chosen to be as similar as possible.

The highest E_{FRET} value, which was reached throughout the experiment was defined as $E_{FRET\text{-}MAX}$.

Evolution of PylRS synthetase for incorporation of TCO-E

A NNK synthetic library, which was based on the *Methanosarcina mazei* $PylRS^{AF}$ variant (Y306A, Y384F) was ordered from GenScript Biotech Corp. with five sites (L305, L309, C348, I405 and W417) mutated to contain any of the 20 amino acids at these positions excluding two stop codons, which leads to 32 possible variants at each of the five sides, resulting in a 3.3 x 10^7 library size. The library was cloned into the selection plasmid, pBK-$PylRS^{AF}$, as mentioned in the previous section (Cloning of constructs), replacing the binding pocket of PylRS with the library gene, resulting in pBK-$PylRS^{lib}$. For the selection pREP-pylT was transformed into DH10B cells and highly competent electro competent cells were prepared freshly, using LB medium with Tet to grow the cells up to OD_{600} of 0.5. After harvesting the cells, they were washed with 10% Glycerol. To resuspend the cells after each harvesting step, the cells should be gently mixed by shaking the harvesting bottles, avoiding any pipetting step. The washing step was repeated two times and finally cells were taken up in as little volume as possible (for 1-liter

expression in 1 ml 10% Glycerol) and aliquoted. 100 ng of pBK-PylRS^{lib} were transformed into 50 µl DH10B (pREP-PylT) cells by electroporation in a 1 mm cuvette and 800 µl of SOC medium were directly added. This step was repeated ten times and cells were combined into a 50 ml shaking flask to incubate for 1 hour at 37 °C shaking at 200 rpm. To estimate the library coverage after the transformation, LB-agar plates were prepared, containing Tet and Kan, and 10 µl of the cell suspension were used to make serial dilutions (1:10^2 to 1:10^7). On each LB-agar plate 100 µl of dilution was plated and incubated overnight in a 37 °C incubator. The rest of the 10 ml transformation mixture was added into 500 ml 2xYT medium, containing Tet and Kan as antibiotics, and incubated over night at 37 °C on a shaker. To estimate the library coverage, 45 colonies on the 1:10^6 plate were counted, which means 4.5 x 10^9 total amount of cells. This yields in a 140-fold coverage of the library size. Next day, the cells were diluted 1:100 in 500 ml fresh 2xYT medium (Tet, Kan) and grown up to OD_{600} of 1, which normally took 2-4 hours. For the 1st positive selection, 10 LB-agar plates (150 mm petri dish) were prepared, containing 1 mM of the TCO-E as well as 60 µg/ml Chloramphenicol (Cm), Tet and Kan. As control, one plate was prepared the same way, just without Cm. The plates were prepared under sterile conditions and cooled down until they were dry. To start the selection, 100 µl of the culture were plated on each 15 mm LB-agar plate, spread by using glass beads and dried next to a flame. The plates were incubated overnight at 37 °C, but not longer than 16 hours. The resulting colonies were scratched from plate with 5 ml 2xYT medium per plate and a cell scraper. The cell suspension of all ten plates was pooled into a 50 ml Erlenmeyer flask and shaken for 1 hour at 37 °C. To isolate the library plasmid, the DNA was extracted first with a Miniprep Kit (Invitrogen) followed by gel extraction. Therefore, the DNA was loaded on a 1% agarose gel and the lowest band was cut. The DNA was isolated by a gel extraction kit (Invitrogen). The plasmid for the negative selection, pYOBB2-PylT, which contains a Barnase gen harboring two amber sites (Gln2 and Asp44), was transformed into DH10B cells and electro competent cells were prepared as mentioned above. 10 ng of gel extracted library plasmid were transformed into 50 µl freshly prepared DH19B (pYOBB2-PylT) cells and recovered after electroporation with 800 µl SOC medium for 1 hour at 37 °C, shaking at 200 rpm in a 14 ml test tube. During the incubation time, the plates for the negative selection were prepared. Six 15 mm petri dishes were casted with LB-Agar, three of them containing 0.2% Arabinose to induce the Barnase expression. All of them containing 50 µg/ml Kan (pBK-PylRS^{lib} plasmid) and 33 µg/ml Cm (pYOBB2-PylT plasmid). 100 µl of the pure cells, as well as 100 µl of a 1:10 and 1:100 dilutions, were plated each on two plates (one with and one without Arabinose). After drying the plates next to a flame, they were incubated overnight in a 37 °C incubator. Colonies were scratched from plates and the DNA was extracted as mentioned above. The positive selection was repeated once more transforming 10 ng of gel extracted library plasmid in 50 µl of DH10B (pREP-PylT), recovering in 800 µl of SOC for 1 hour shaking at 37 °C. This time only three 15 mm LB-agar plates containing 33 µg/ml Cm (Kan, Tet) with 1 mM TCO-E and one control plate without TCO-E were used for the selection. 100 µl of cell suspension were plated and incubated overnight at 37 °C. The surviving colonies were scratched from plate and the library plasmid was extracted out of a 1% agarose gel. To test the amber suppression efficiency of the remaining PylRS variants, an expression test based on superfolder GFP (sfGFP) was performed. Therefore, the library DNA was transformed together with the plasmid pALS-sfGFP^{N150TAG}-MbPyl-tRNA, encoding sfGFP with an amber site at position N150 and the tRNA^{Pyl} from *Methanosarcina barkeri* into DH10B cells. To include some controls, the pBK-PylRS plasmid, encoding for PylRS^{WT} as well one encoding or PylRS^{AF} were also co-transformed with the pALS plasmid. The transformations were recovered in SOC medium for 1 hour and different amounts (50, 100 and 200 µl) were plated on auto-inducing minimal media plates (Kan, Tet) with 1 mM TCO-E and without ncAA. Colonies were allowed to grow for 24 hours at 37 °C and if needed, another 24 hours at RT. Green colonies were picked and grown overnight in a 96-well plate, containing 480 µl of non-inducing minimal media in each well. The controls (PylRS^{WT} and PylRS^{AF}) were also picked and included in this 96-well plate. Next day, two 96-well plates were prepared, each with 480 µl of auto-inducing minimal medium per well, one plate with 1 mM TCO-E and one plate without. 20 µl of each overnight culture were pipetted into corresponding wells in the new 96-well plates and incubated for 24 hours at 37 °C shaking at 250 rpm. The sfGFP expression was analyzed with a fluorescent measurement (BIOTEK Synergy 2 Microplate Reader). Therefore, a 96-well plate, containing 180 µl water per well was prepared, in which 20 µl of the sfGFP expression culture was pipetted in the corresponding well. To correct for different OD_{600} of each expression, also an optical density scan at 600 nm was performed (150 µl water + 50 µl expression culture).

Out of the 96-well plate with the non-inducing minimal media, each well of interest can be analyzed further, e.g. the DNA of the PylRS variant can be extracted to send for sequencing and further subcloning.

Synthetic procedures

General Information for TCO*N and TCO*C-E:

Unless otherwise stated, reagents were purchased from general chemical suppliers (Sigma-Aldrich (Merck), Alfa Aesar, Abcr and used as received. Anhydrous solvents (DMF, DCM, MeCN, THF) were purchased from Sigma-Aldrich in sealed bottles and transferred in reaction vessel via syringe under inert atmosphere of argon. Reactions were monitored by TLC analysis (pre-coated silica gel 60 F_{254} plates, 500 µm layer thickness) and visualization was accomplished with a 254 nm UV light and by staining with a $KMnO_4$ solution (1.5 g of $KMnO_4$, 10 g of K_2CO_3, and 1.25 mL of a 10% NaOH solution in 200 ml of water). Reactions were also monitored with UPLC-MS (Acquity UPLC BEH C18 column: 1.7 µm; 2.1x50 mm; T = 40 °C; flow rate: 1.5 ml/min; inject volume 3 µl; runtime 3 min; A: water plus 0.1% TFA, B: MeCN plus 0.1% TFA; method: 0 – 2.3 min 5-100% B, 2.3 – 2.5 min 100% B, 2.5 – 2.6 min 100-5% B, 2.6 – 3.0 min 5% B) on Agilent Infinity 1290 series equipment set up (Agilent 1290 quaternary pump and sampler, 1290 thermostated column compartment and 1290 Diode array detector VL) equipped with a quadrupole LC/MS 6120 and an Infinity 1260 ELSD. Milli-Q quality water used, was obtained after filtration of distilled water through a Milli-Q® cartridge system.

Flash chromatography on SiO_2 was used to purify the crude reaction mixtures and performed on the Biotage Isolera Four flash system utilizing pre-packed cartridges and linear gradients. ^{1}H and ^{13}C NMR spectra were obtained on a Bruker 400 MHz instrument in $CDCl_3$, CD_3OD or DMSO-d6 (deutero.de) unless otherwise noted. Chemical shifts were reported in parts per million with the residual solvent peak used as an internal standard ($CDCl_3$ = 7.26 ppm for ^{1}H and 77.16 ppm for ^{13}C; CD_3OD = 3.31 ppm for ^{1}H and 49.00 ppm for ^{13}C; DMSO-d6 = 2.50 ppm for ^{1}H and 39.52 ppm for ^{13}C). ^{1}H NMR spectra were run at 400 MHz and are tabulated as follows: chemical shift, multiplicity (s = singlet, d = doublet, t = triplet, q = quartet, p = pentet, m = multiplet, bs = broad singlet, dt = doublet of triplet, tt = triplet of triplet), number of protons, and coupling constant(s). ^{13}C NMR spectra were run at 100 MHz using a proton-decoupled pulse sequence with a d1 of 0 second unless otherwise noted, and are tabulated by observed peak. High-resolution mass spectra were obtained on a Bruker Apex-Qe mass spectrometer using electrospray ionization (ESI).

Continuous flow synthesis of trans-cyclooctenes

To be able to perform efficient photoisomerization of cis-cyclooctenes we have built a continuous flow photoreactor. Inspired by work of Fox[6] and Mikula[7] et. al, we thought to further optimize the existing designs and additionally, improve on the safety features. Based on the previous observations, cooling of the system improves stereo-selectivity of the isomerization reaction[8]. Enhanced cooling of the reaction mixture is achieved by distinct design features of this reactor: re-packable glass column with a cooling jacket and fan-ventilated light-impermeable aluminum wall of the UV-unit casing (Figure below: In house built photoreactor). Fan ventilation is necessary to dissipate the heat from the lamps and prevent evaporation of the solvents (hexanes, Et_2O) during the long runs. Additionally, cooling bath can be applied to the reaction flask. Electrical unit allows independent switching of the low pressure Hg-lamps of variable intensity (2x55 W and 1x95 W) to control the intensity of the UV-light (Figure below, 5). The lamps are positioned closely around the quartz tube to allow for efficient light penetration. This minimizes the space required for the UV-unit and eliminates the need for expansive quartz flasks.

In house built photoreactor:

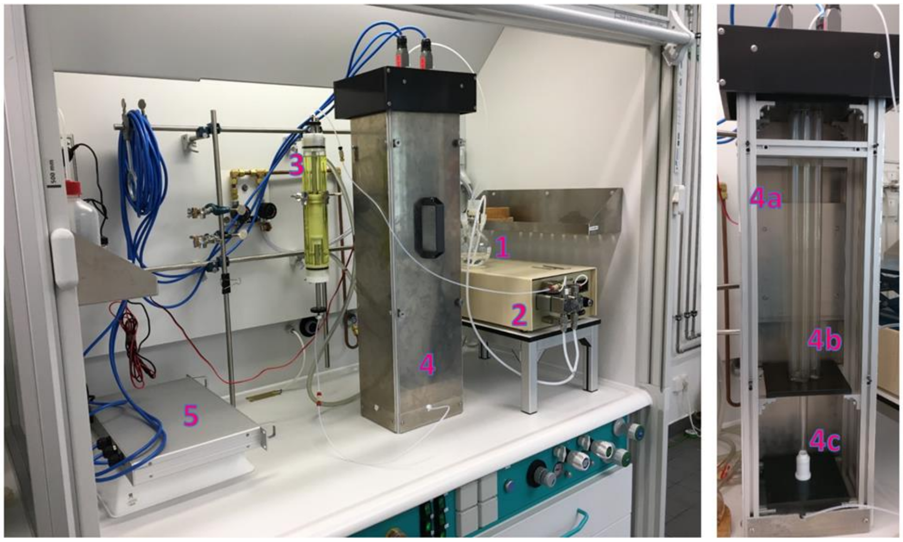

Left – continuous flow photoisomerization setup: (1) three-neck round-bottom glass flask; (2) HPLC pump; (3) re-packable glass column with cooling jacket (4) UV-unit in aluminum case; (5) electrical unit; right – quartz tube (4c) surrounded by three low pressure Hg-lamps (4b); (4a) air fan behind aluminum plate.

Additional details: Quartz tube of desired dimensions (OD 16 mm, ID 14 mm) was made to order by QSIL GmbH (https://www.qsil.com/en/products-solutions/tubes/). The tube was capped with tightly fitting Teflon adaptors containing an O-ring and threaded hole (made in house) for the attachment of the flanged Teflon tubing via threaded PEEK adapters. HPLC pump (Model P402), glass column with cooling jacket and flanged tubing with adapters were purchased from Latek Labortechnik-Geräte GmbH & Co. (http://www.latek.de/index-2_LC_GLS.html, http://www.latek.de/index-2_kapillaren.html, http://www.latek.de/index-2_pumpen.html). UV lamps (40 mm x 533 mm, two of HNS L 55W 2G11 and one HNS L 96W 2G11) and their electrical adapters were purchased from Osram Licht AG (https://www.proflamps.de/datasheets/ZMP_1164638.pdf, https://www.proflamps.de/datasheets/ZMP_1164636_ZMP_1164636_HNS-L_55W_2G11_1st%20(1).pdf)

General procedure for the synthesis of trans-cyclooctenes using in-house built photoreactor.

Glass column with a cooling jacket was packed with an oven-dried unmodified SiO_2 (bottom; 2 g per mole of the starting material *cis*-cyclooctene) and 10%$AgNO_3$/SiO_2 (top; 2 g per mole of the starting material *cis*-cyclooctene). The column was equilibrated at 6-10 °C at 100 ml/min with 5:1 Et_2O-hexanes for 30 minutes using HPLC pump. Starting material *cis*-cyclooctene (1.0 equiv.) was placed on the ice bath in the 3-neck flask and dissolved in 5:1 Et_2O-hexanes (~30 ml per mole of starting material *cis*-cyclooctene). The continuous flow was achieved between flask, quartz tube and column and the solution was equilibrated at 6-10 °C at 100 ml/min for 20 minutes. Methyl benzoate (2.0 equiv.) was added into the flask and equilibration continued for another 30 minutes while degassing with nitrogen. The quartz tube was then irradiated with UV light under 100 ml/min continuous flow of the reaction mixture (4-8 h). The reaction progress was monitored by the consumption of the starting material using TLC (EtOAc/heptane 2:1). After completion of the reaction the UV light was switched off and the flow continued for another 10 minutes. The column was briefly flushed with 5:1 Et_2O-hexanes and Et_2O then dried using nitrogen. The content of the column was then transferred into Erlenmeyer`s flask containing NaCl (4 g per mole of the starting material *cis*-cyclooctene) and ice-water slurry (10 ml per mole of the starting material *cis*-cyclooctene) and stirred vigorously. Ammonium hydroxide (2.0 equiv.) was added as 25% aqueous solution and resultant heterogeneous mixture was vigorously stirred for 1 hour. The mixture was then filtered and the filter cake was excessively washed with Et_2O. Aqueous layer was extracted with Et_2O twice and combined ethereal phase was dried over magnesium sulfate, filtered and evaporated to afford corresponding *trans*-cyclooctene in purity sufficient for the next step.

Synthesis of TCO*N

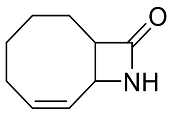

***(Z)*-9-azabicyclo[6.2.0]dec-6-en-10-one (12)**[9]

To a cooled (0 °C, ice bath) stirred solution of cycloocta-1,3-diene (3.0 ml, 24.1 mmol) in 6.0 ml of anhydrous DCM under an inert atmosphere was slowly added chlorosulfonyl isocyanate (CSI, 2.1 ml. 24.1 mmol). Reaction mixture was allowed to warm up and stirred at RT overnight (14 h). Reaction was then diluted with 3 ml of DCM, drawn into a syringe and added slowly into a vigorously stirring mixture of DCM-H_2O (40 ml, 1:1) and 5.2 g of Na_2SO_3. Simultaneously, via another syringe 10% w/w KOH solution was added so that pH was maintained between 6-8. Resultant reaction mixture was vigorously stirred for 2 hours at RT in an opened flask and then transferred into a separatory funnel. Organic layer (as milky suspension) was separated and aqueous layer was extracted with DCM (150 ml x 4). Combined DCM was removed *in vacuo* and resultant solid was purified by flash column chromatography on SiO_2 (EtOAc/heptane, 10 to 100% EtOAc) to provide 3.10 g (85%) of **12** as a white solid: rf = 0.29 (EtOAc-heptane, 2:1); ^{1}H NMR (400 MHz, $CDCl_3$) δ 6.16 (s, 1H), 5.91 – 5.55 (m, 1H), 5.39 (dd, *J* = 11.5, 2.3 Hz, 1H), 4.53 (dt, *J* = 5.0, 2.3 Hz, 1H), 3.31 (dd, *J* = 12.5, 5.4 Hz, 1H), 2.10 – 1.97 (m, 2H), 1.95 – 1.82 (m, 2H), 1.78 – 1.56 (m, 2H), 1.48 – 1.35 (m, 2H); ^{13}C NMR (101 MHz, $CDCl_3$) δ 171.33, 133.64, 125.89, 58.65, 51.43, 31.49, 29.22, 25.88, 22.59; in a similar experiment 3.0 ml of cycloocta-1,3-diene provided 2.86 g of the product **(12)** in 78% yield.

^{1}H NMR:

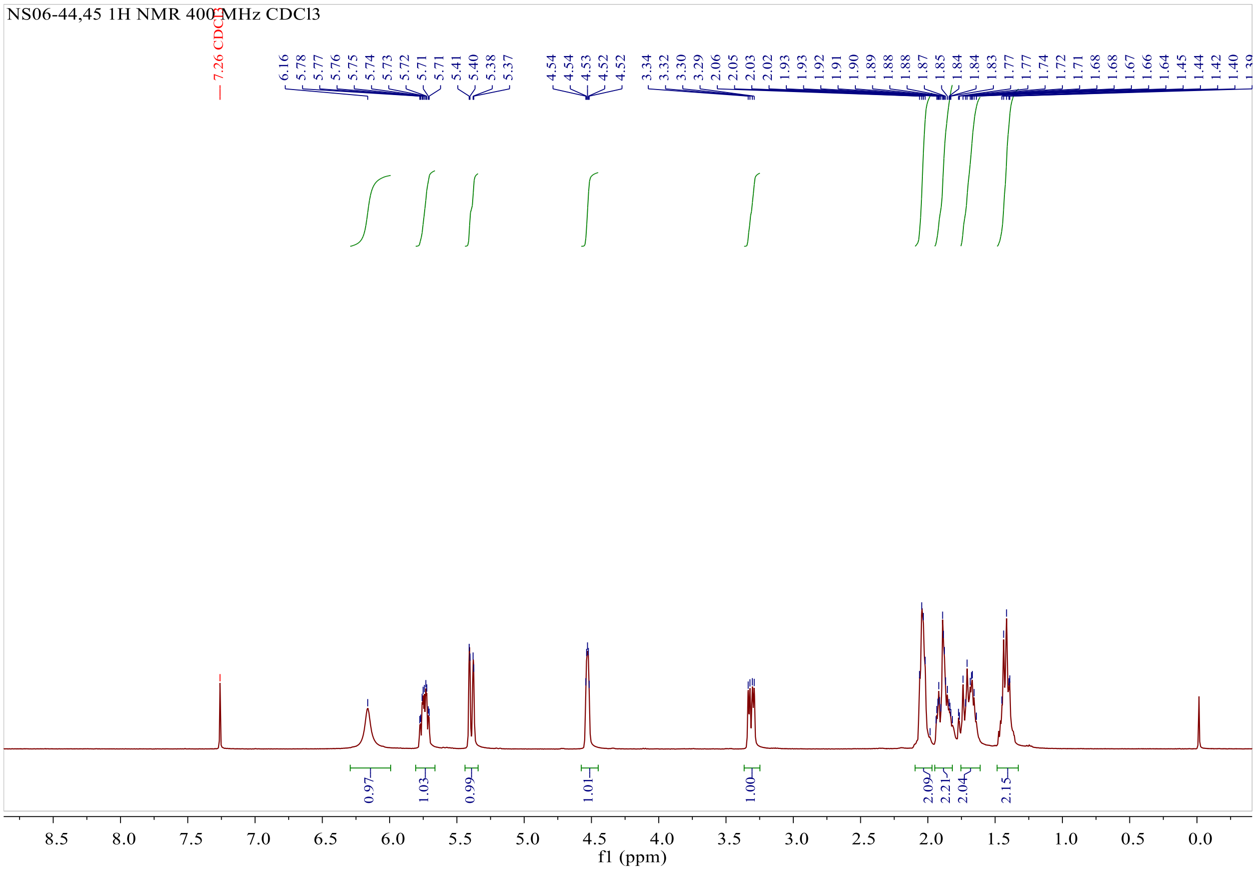

^{13}C NMR:

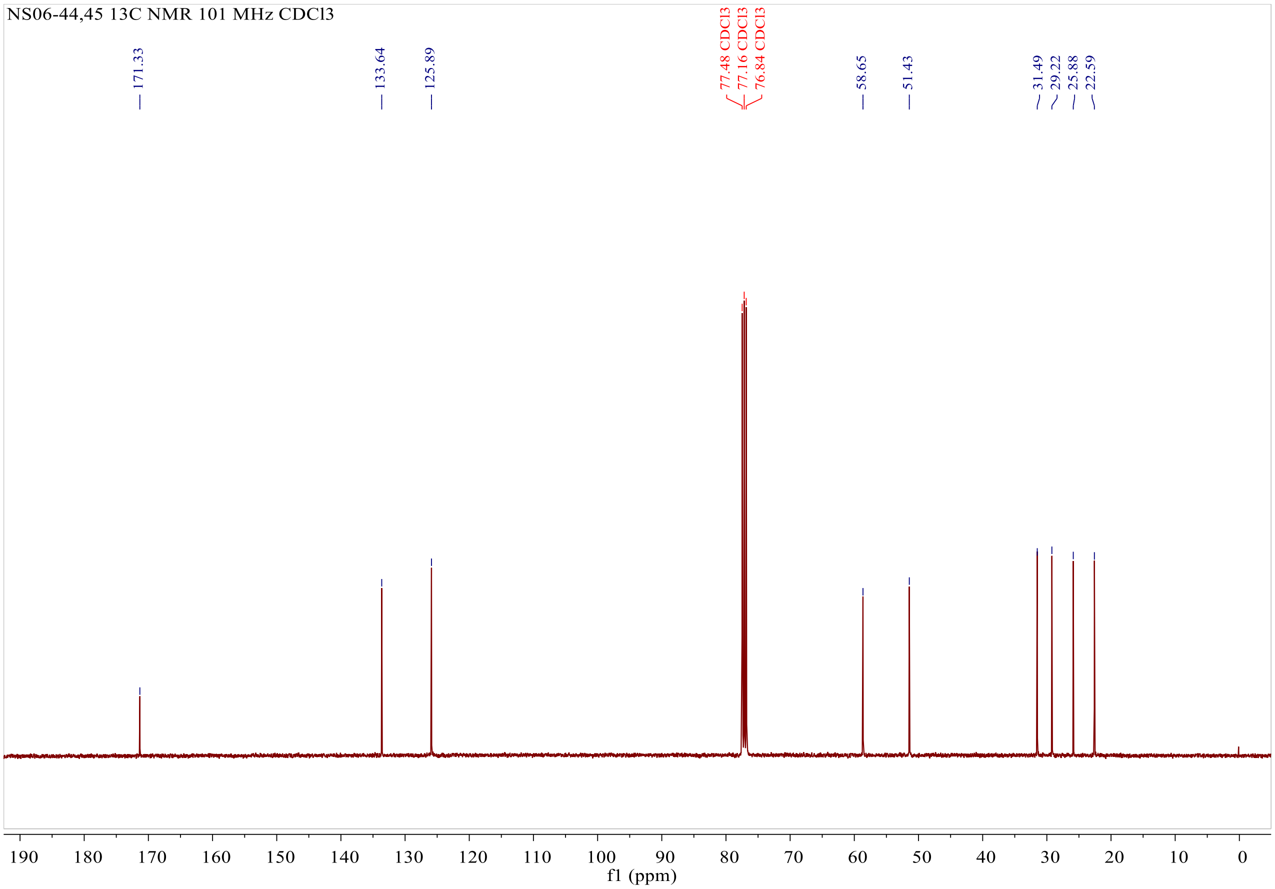

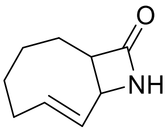

rac-*(E)*-9-azabicyclo[6.2.0]dec-6-en-10-one (13)

According to general procedure (1.60 g, 10.6 mmol) provided **13** (1.41 g, 88%) as a white solid as a 5:1 mixture of atropisomers: rf = 0.30 (EtOAc-heptane, 2:1); highlighted in bold are distinct NMR peaks of a major stereoisomer: ^{1}H NMR (400 MHz, $CDCl_3$) δ **6.60** (br s, 2H), 6.30 (br s, 1H), 6.20 (td, J = 10.5, 5.1 Hz, 1H) **5.70 – 5.49** (m, 5H), **4.39** (t, J = 5.4 Hz, 2H), 4.23 (t, J = 4.0 Hz, 1H), **3.67** (td, J = 5.0, 3.7 Hz, 2H), 3.56 – 3.46 (m, 1H), 2.46 – 2.37 (m, 2H), 2.30 – 2.10 (m, 2H), 2.05 – 1.90 (m, 4H), 1.88 – 1.33 (m, 4H), 1.31 – 1.18 (m, 1H), 1.15 – 1.07 (m, 1H), 1.06 – 0.98 (m, 1H); ^{13}C NMR (101 MHz, $CDCl_3$) δ **172.48**, 171.91, **136.15**, 134.84, **134.78**, 131.60, **62.88**, 62.05, **52.28**, 50.76, 38.14, 36.18, 34.48, 31.81, 28.26, 28.11, 23.57, 23.28; HRMS (ESI): *m/z* calcd for $C_9H_{12}NO$, $[M–H]^-$ 150.0913, found 150.0907; in a similar experiment 1.60 g of **12** provided 1.29 g the product **(13)** in 81% yield.

^{1}H NMR:

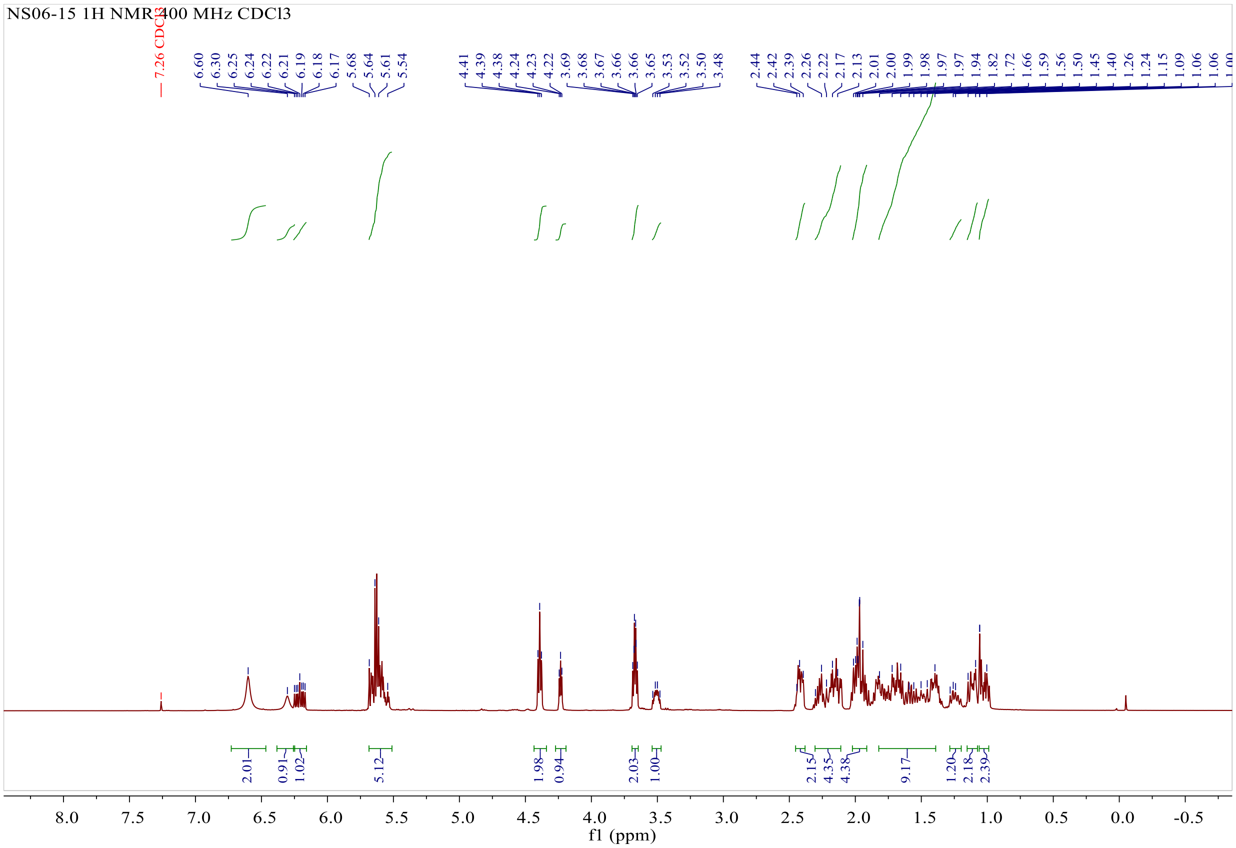

^{13}C NMR:

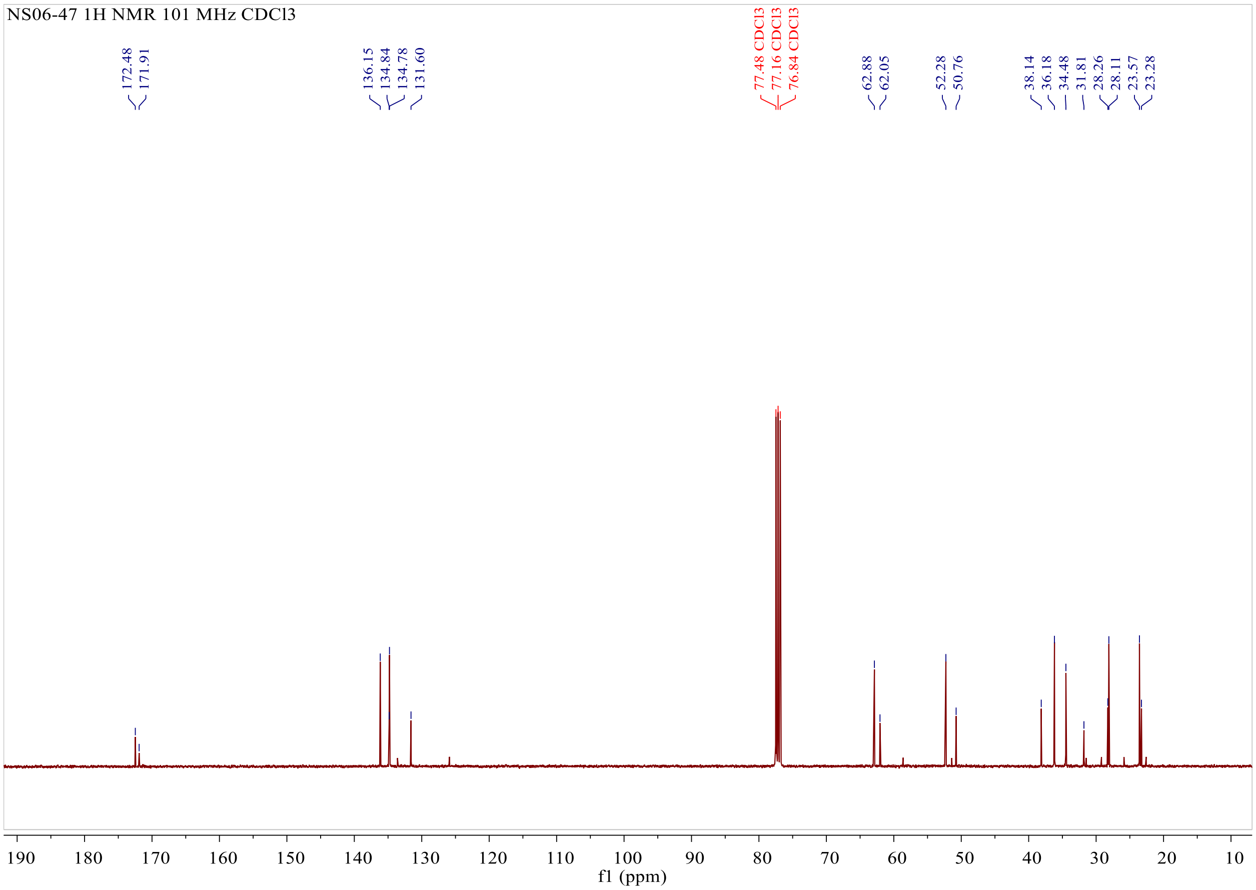

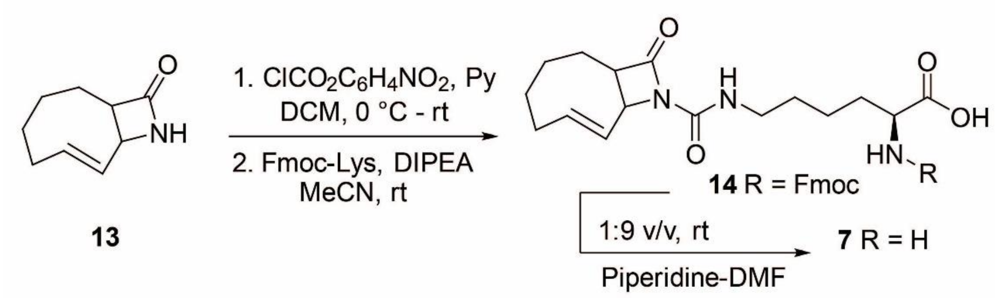

N6-((E)-10-oxo-9-azabicyclo[6.2.0]dec-6-ene-9-carbonyl)-L-lysine (7)

13 (137 mg, 0.91 mmol, 1.0 equiv.) was dissolved in dry DCM (0.1 M) and the solution was placed in an ice bath and cooled to 0 °C under an inert atmosphere of argon. Pyridine (184 µl, 2.28 mmol, 2.50 equiv.) was added and the reaction mixture was stirred for 5 minutes. 4-Nitrophenylchloroformate (204 mg, 1.01 mmol, 1.1 equiv.) in 1.0 ml of dry DCM was added slowly dropwise, ice bath was removed and the reaction mixture allowed to warm up and stirred at RT for 6 hours. Solvent was removed *in vacuo* and crude orange oil was dissolved in dry acetonitrile (18 ml) under an inert atmosphere of argon. Fmoc-lysine hydrochloride (387 mg, 0.96 mmol, 1.05 equiv.) was then added in one portion followed by slow, dropwise addition of DIPEA (476 µl, 2.73 mmol, 3.0 equiv.) and reaction mixture was stirred at room temperature overnight (19 h). The reaction mixture was concentrated *in vacuo* crude residue was well mixed with 3 g SiO_2 in small amount of DCM, excess of DCM was removed *in vacuo* and resultant yellow solid was semi-purified by flash column chromatography on SiO_2 (DCM/MeOH, 1 to 11% MeOH) to provide **14** as a white foamy oil which was immediately carried to the next step. **14** was dissolved in 3.30 ml of DMF and piperidine (0.36 ml, 3.67 mmol) was added dropwise. Reaction mixture was stirred at RT for 45 minutes. DMF and excess piperidine were then removed *in vacuo* and the resultant crude material was purified by reverse phase

chromatography on C18 SiO_2 (H_2O/CH_3CN, 0.1% TFA additive, 1 to 25% CH_3CN) to provide 13.6 mg (3.4 %, TFA salt; 3 steps) of **7** as a white hygroscopic solid: ^1H NMR (400 MHz, MeOD) major: δ 5.83 – 5.71 (m, 1H), 5.66 (dd, ***J* = 17.1**, 6.0 Hz, 1H), 4.80 (t, *J* = 5.9 Hz, 1H), 3.97 (t, *J* = 6.3 Hz, 1H), 3.83 (td, *J* = 5.4, 3.5 Hz, 1H), 3.30 – 3.25 (m, 1H), 2.54 – 2.43 (m, 1H), 2.24 – 2.12 (m, 1H), 2.09 – 1.84 (m, 4H), 1.77 – 1.41 (m, 7H), 1.24 – 1.03 (m, 2H); ^{13}C NMR (101 MHz, MeOD) δ 171.96, 171.82, 152.55, 61.76, 55.73, 53.81, 40.17, 36.78, 35.40, 31.13, 30.41, 28.20, 24.68, 23.21; HRMS (ESI): *m/z* calcd for $C_{16}H_{26}N_3O_4$, $[M+H]^+$ 324.1918, found 324.1916; in a similar experiment 226 mg of **13** provided 21.5 mg of the product **(7)** in 3.3% yield.

^1H NMR:

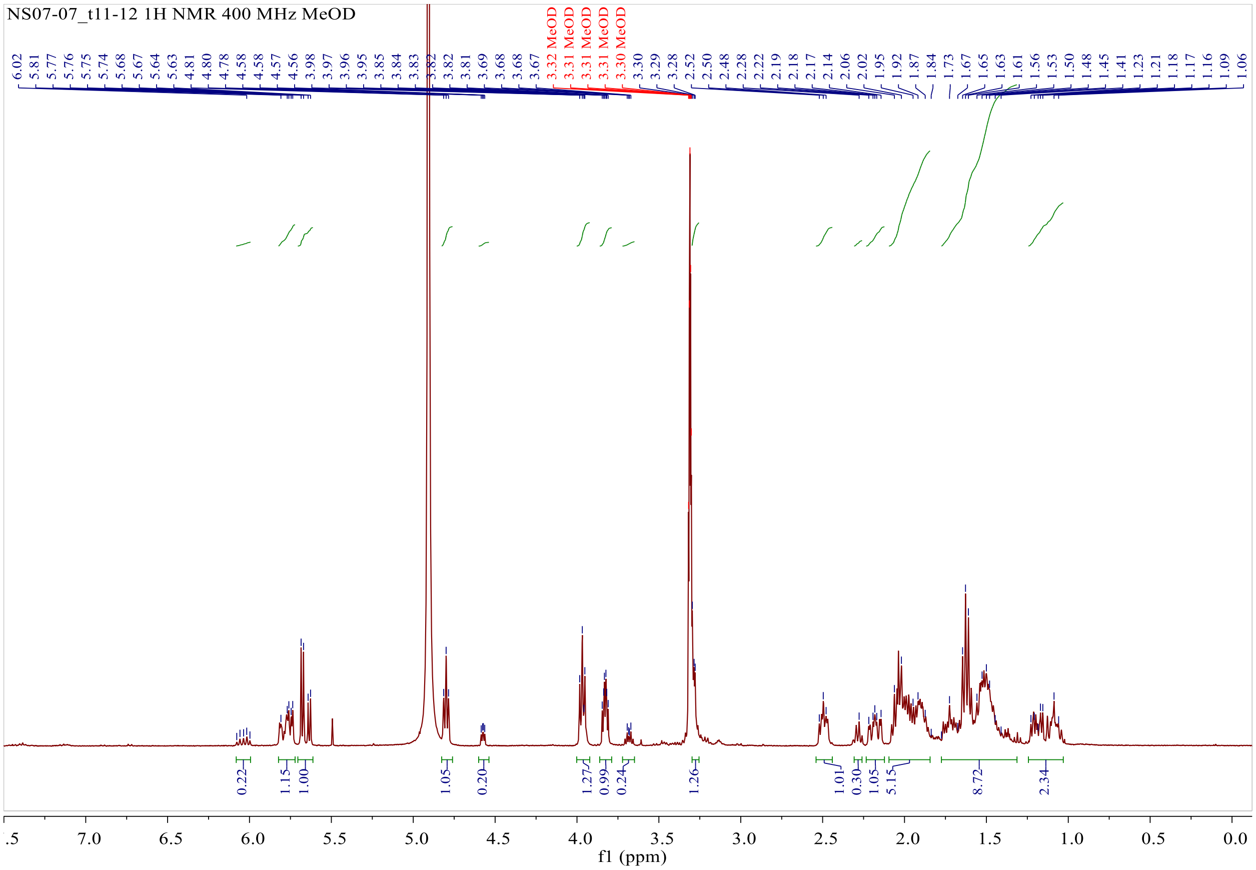

^{13}C NMR:

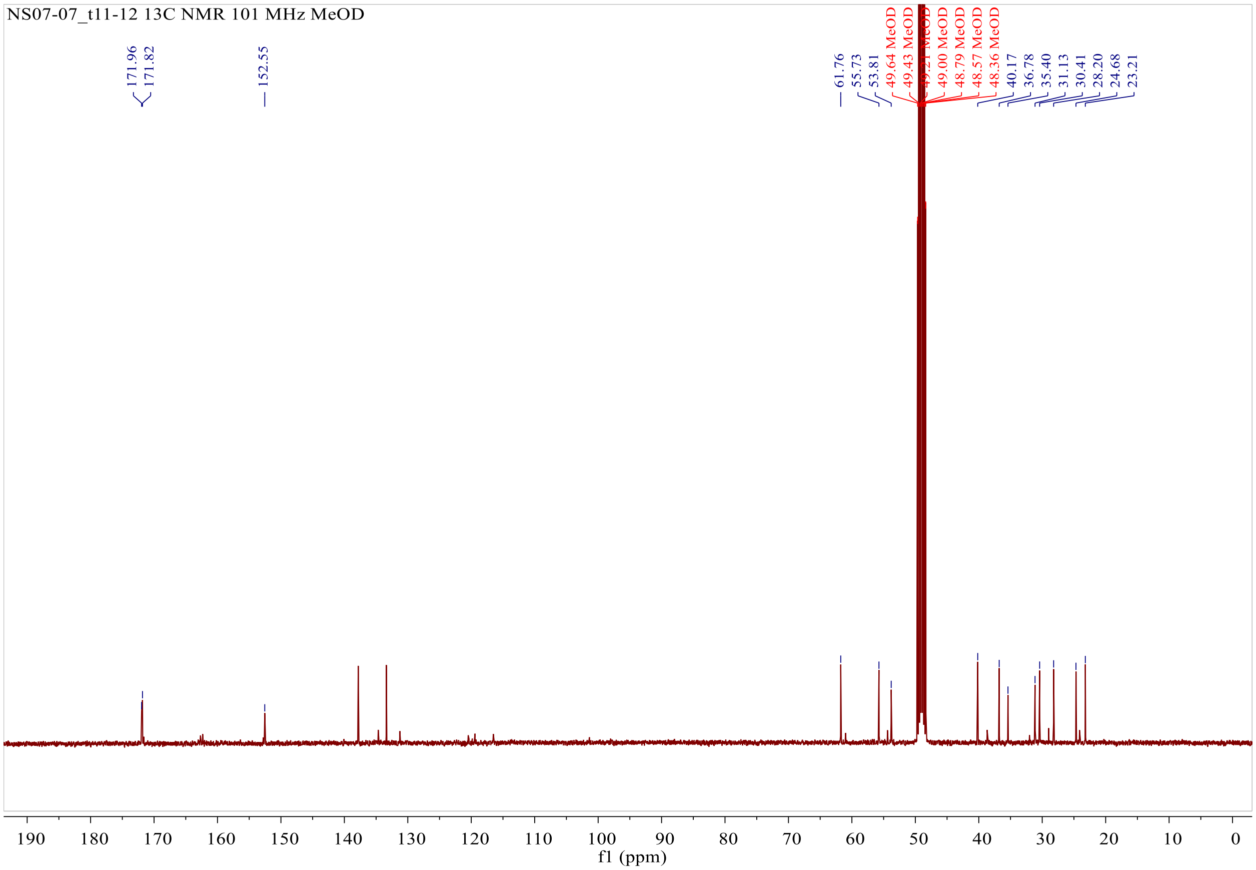

Synthesis of TCO*C-E:

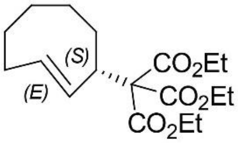

triethyl-cyclooct-2-en-1-ylmethanetricarboxylate (15)

To a solution of commercially available *trans*-TCO-OH equatorial alcohol (4.10 mmol, 520 mg) in toluene (10 mL) was added PMe_3 (1 M in toluene, 7 ml) and triethyl methanetricarboxylate (6.2 mmol, 1.3 ml) at 0 °C followed by slow addition of DEAD (3.19 ml, 7.0 mmol) as 40% v/v solution in toluene, and the resulting solution was then heated at 90 °C for 3 hours. Toluene was then evaporated *in vacuo* and resultant orange oil was dry loaded on 3 g of SiO_2 and purified by flash column chromatography on SiO_2 (EtOAc/heptane, 1 to 20% EtOAc) to provide 1.21 g (86%) of **15** as clear oil. ^1H NMR (400 MHz, $CDCl_3$) δ 5.65 (ddd, ***J* = 15.9**, 10.5, 3.5 Hz, 1H), 5.55 (dd, ***J* = 15.9**, 10.1 Hz, 1H), 4.32 – 4.19 (m, 6H), 3.06 (ddd, *J* = 11.4, 10.0, 4.5 Hz, 1H), 2.42 – 2.32 (m, 1H), 2.25 – 2.14 (m, 1H), 2.01 – 1.75 (m, 4H), 1.61 – 1.37 (m, 2H), 1.34 – 1.22 (m, 9H), 0.95 – 0.74 (m, 2H); ^{13}C NMR (101 MHz, $CDCl_3$) δ 166.72, 135.38, 131.04, 67.73, 61.88, 51.36, 37.41, 35.75, 35.61, 29.56, 29.04, 14.07; HRMS (ESI): *m/z* calcd for $C_{18}H_{29}O_6$, $[M+1]^+$ 341.1959, found 341.1961; in a similar experiment 520 mg of *trans*-TCO-OH equatorial alcohol 1.10 g of the product **(15)** in 78% yield.
In this reaction we obtained only one stereoisomer. This is most likely the equatorial isomer as for Mitsunobu reactions S_N2' type mechanisms have been described that lead to stereochemical[10]. This could be further investigated by obtaining a crystal structure, however as the final amino acid did not exhibit high reaction rate constants we did not investigate this compound further.

^{1}H NMR:

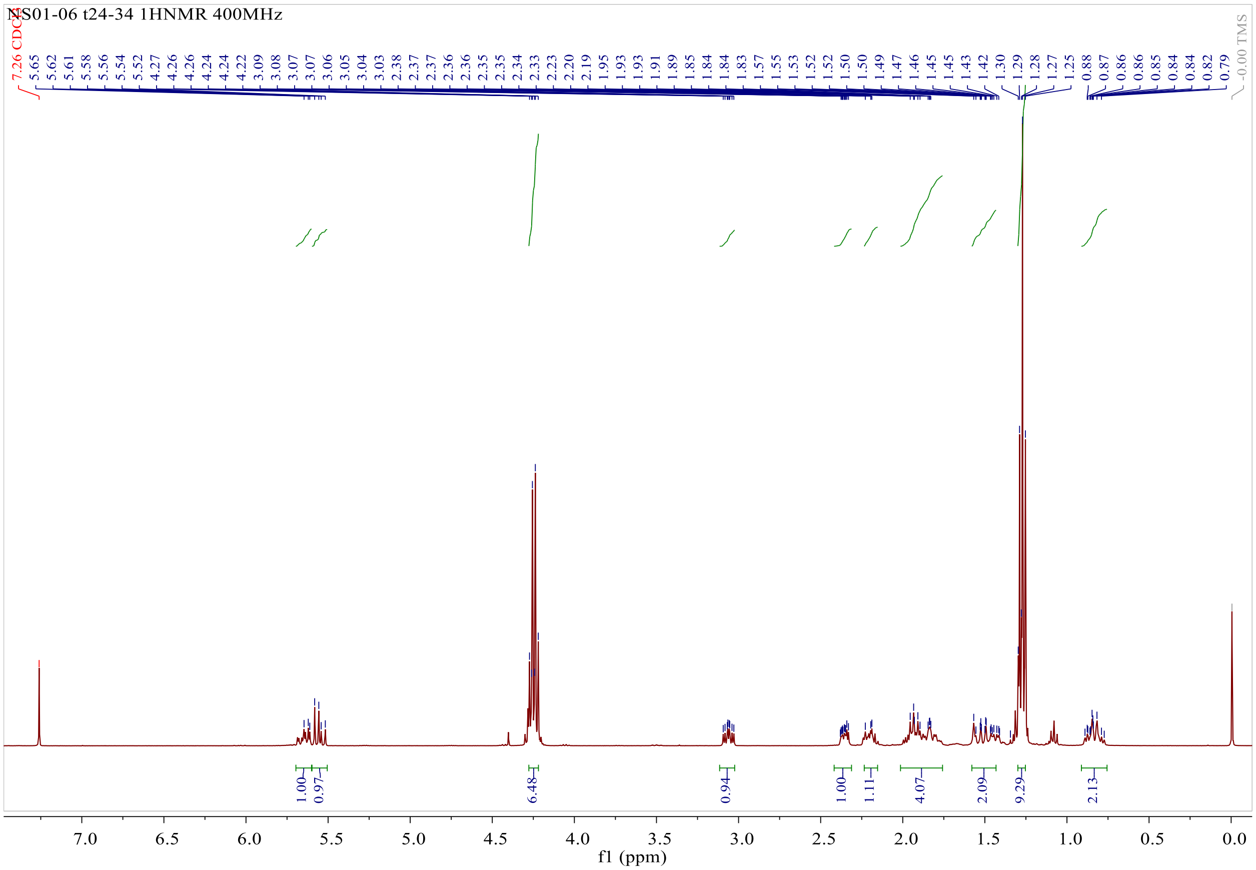

NS01-06 t24-34 1HNMR 400MHz
7.26 CDCl3
5.65
5.62
5.61
5.58
5.56
5.54
5.52
4.27
4.26
4.26
4.24
4.24
4.22
3.09
3.08
3.07
3.07
3.06
3.05
3.04
3.03
2.38
2.37
2.37
2.36
2.36
2.35
2.35
2.34
2.33
2.23
2.20
2.19
1.95
1.93
1.93
1.91
1.89
1.85
1.84
1.84
1.83
1.57
1.55
1.53
1.52
1.52
1.50
1.50
1.49
1.47
1.46
1.45
1.45
1.43
1.42
1.30
1.29
1.28
1.27
1.25
0.88
0.87
0.86
0.86
0.85
0.84
0.84
0.82
0.79
-0.00 TMS
1.00
0.97
6.48
0.94
1.00
1.11
4.07
2.09
9.29
2.13
7.0
6.5
6.0
5.5
5.0
4.5
4.0
3.5
3.0
2.5
2.0
1.5
1.0
0.5
0.0
f1 (ppm)

^{13}C NMR:

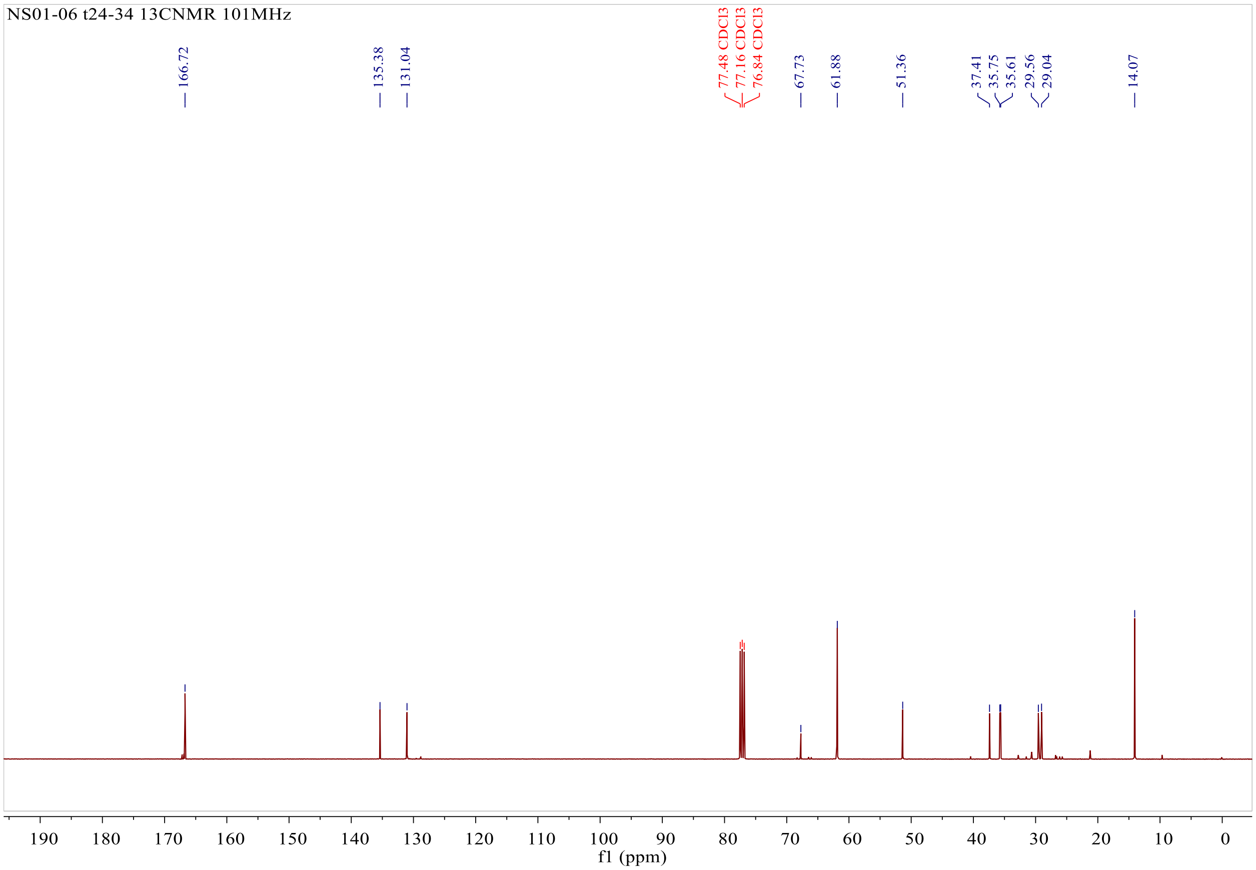

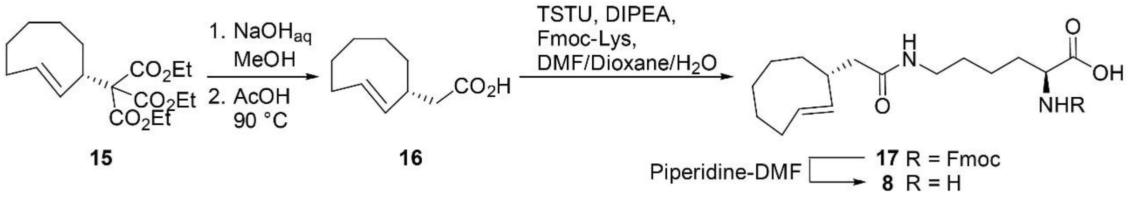

N6-(2-((*E*)-cyclooct-2-en-1-yl)acetyl)-*L*-lysine (8)

15 (511 mg, 1.5 mmol) was dissolved in 8 ml of MeOH and treated with a 10 fold excess of 3 M NaOH (5 ml, 15 mmol). The reaction was heated under reflux (90 °C) for 25 hours. MeOH was evaporated at RT and the crude product was suspended in 16 ml of AcOH, briefly sonicated and heated at 90 °C for 24 h. The reaction mixture was extracted with excess pentane and the solvent evaporated to give crude **16** which was immediately carried to the next step. To a solution of **16** (67.0 mg, 0.398 mmol) in 0.8 mL DMF, 0.8 ml of Dioxane and 0.4 ml water were added sequentially. DIPEA (196 µl, 1.12 mmol) and TSTU (141 mg, 0.468 mmol) were added and the reaction mixture stirred at RT for 15 min. Then Fmoc-Lys (271 mg, 0.562 mmol) in 1 ml DMF was added and the mixture stirred for 6 h at RT. 8 ml of water were added and the resultant milky suspension was freeze dried. The oily residue was redissolved in DCM and purified by flash column chromatography on SiO_2 ((MeOH-AcOH 3:1)/DCM, 1 to 20%) to provide **17**. Fmoc-protected UAA **17** (53.0 mg, 0.102 mmol) was dissolved in 0.8 ml DMF and 0.2 ml piperidine was added dropwise. The reaction mixture was stirred at RT for 1 h. The solvent was evaporated and the resulting white solid resuspended in 10 ml of water and sonicated. The aqueous solution was washed with DCM (2 x 5 ml) and the aqueous phase freeze dried and the product purified using reversed phase chromatography to provide 26.1 mg (14% over 3 steps) of **8** as white solid: ^1H NMR (400 MHz, MeOD) δ 5.59 (ddd, ***J* = 15.2**, 10.9, 3.7 Hz, 1H), 5.26 (dd, ***J* = 15.9**, 10.2 Hz, 1H), 3.92 (td, *J* = 6.4, 2.7 Hz, 1H), 3.26 – 3.09 (m, 2H), 2.63 – 2.48 (m, 1H), 2.40 – 2.30 (m, 1H), 2.29 – 2.15 (m, 2H), 2.01 – 1.79 (m, 6H), 1.61 – 1.38 (m, 5H), 1.34 – 1.13 (m, 2H), 0.98 – 0.79 (m, 2H); ^{13}C NMR (101 MHz, MeOD) δ 175.53, 171.80, 136.95, 135.04, 53.81, 46.88, 43.64, 42.66, 39.68, 36.80, 36.55, 31.13, 30.29, 30.02, 23.31; HRMS (ESI): *m/z* calcd for $C_{16}H_{29}N_2O_3$ 297.2173, found 297.2175.

^{1}H NMR:

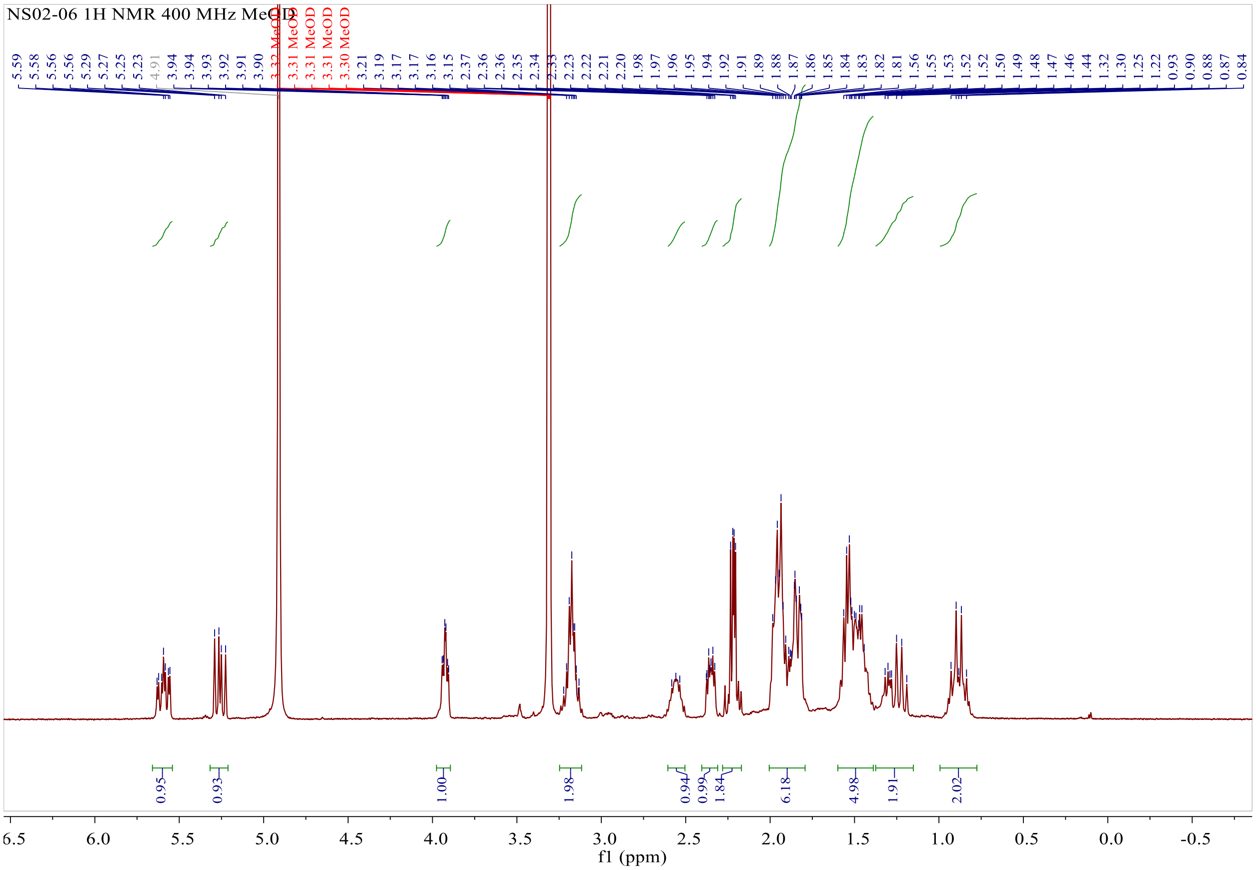

^{13}C NMR:

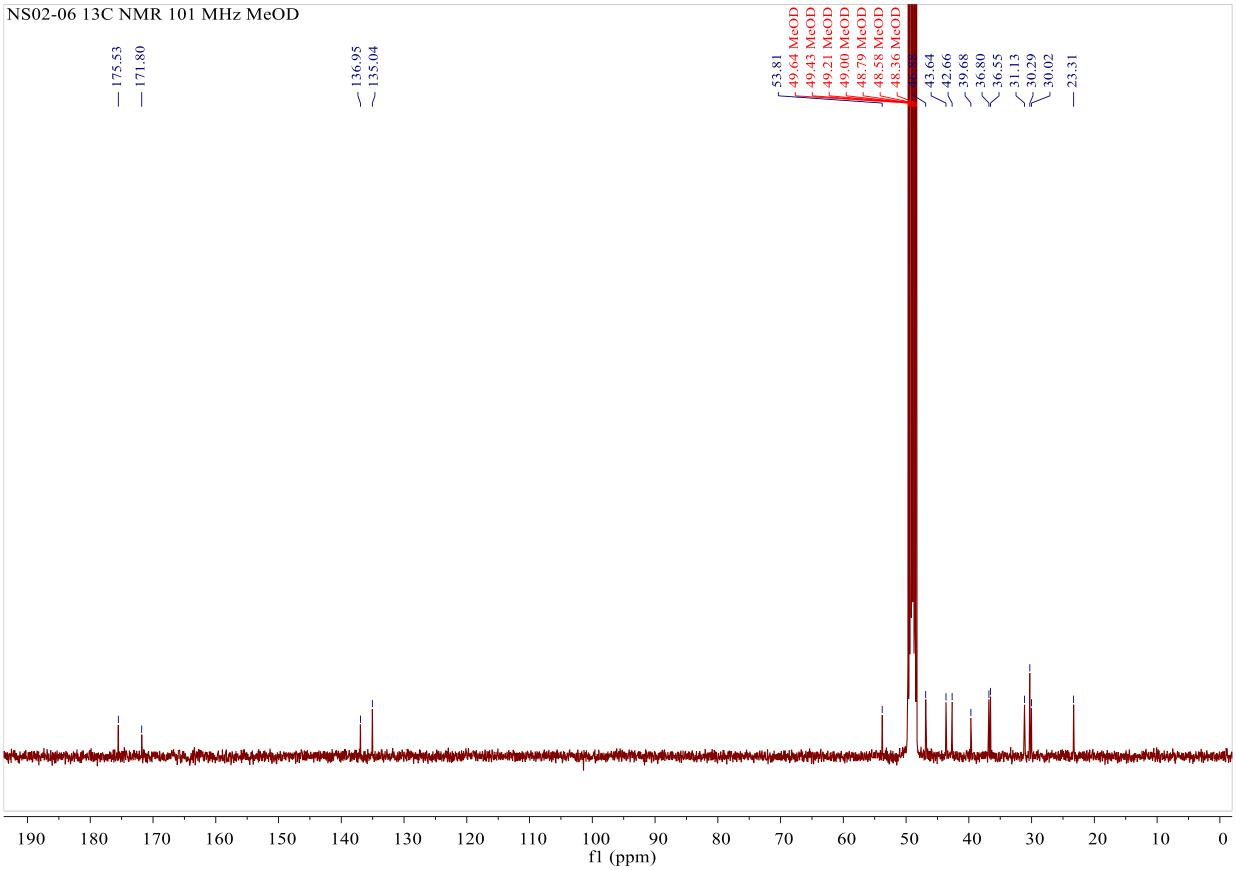
NS02-06 13C NMR 101 MHz MeOD
175.53
171.80
136.95
135.04
53.81
49.64 MeOD
49.43 MeOD
49.21 MeOD
49.00 MeOD
48.79 MeOD
48.58 MeOD
48.36 MeOD
43.64
42.66
39.68
36.80
36.55
31.13
30.29
30.02
23.31
190 180 170 160 150 140 130 120 110 100 90 80 70 60 50 40 30 20 10 0
f1 (ppm)

General Information for AmTCO:

All solvents and reagents were used as supplied without further purification.
Ultra performance liquid chromatography coupled to mass spectrometry (UPLC/MS) analyses were carried out on a Waters Acquity system (detectors: Acquity SQD, Acquity ELSD, Acquity PDA) fitted with a Waters Acquity UPLC BEH C18 column (1.7 µm, 2.1 mm x 50 mm). Water + 0.2% formic acid (A) and acetonitrile + 0.2% formic acid (B) were used as eluents. The analyses were performed at a flow rate of 0.5 mL/min with a linear gradient from 5 to 100% over 4 min. Electrospray mass spectra were acquired in positive/negative ion alternate mode. The purity of compounds was determined by detection at 254 nm.
High Resolution Mass Spectrometry analyses (HRMS) were performed with a Q Exactive™ Plus Hybrid Quadrupole-Orbitrap™ Mass Spectrometer.
Preparative high performance liquid chromatography (prep HPLC) was performed on a Waters autopurification system (2767 Sample manager, 2545 Binary Gradient Module, 2420 ELS Detector, 2996 Photodiode Array Detector, 3100 Mass Detector, Waters SFO, Waters 515 HPLC Pump) fitted with a XBridge BEH C18 OBD prep column (5 µm, 19 mm x 150 mm). The purifications were carried out at a flow rate of 30 mL/min using water and acetonitrile as eluents with 0.2% formic acid as modifier.
Nuclear magnetic resonance (NMR) analyses were acquired on a Bruker Avance 400 MHz spectrometer equipped with a 5 mm PABBO BB/19F-1H/D Z-GRD probe at 295 K. The deuterated solvent was used as an internal deuterium lock. Chemical shifts (δ) are reported in parts per million using residual non-deuterated solvents as internal references. Spectra were processed and visualized with MestReNova 14.1.0.

Synthesis of AmTCO amino acids:

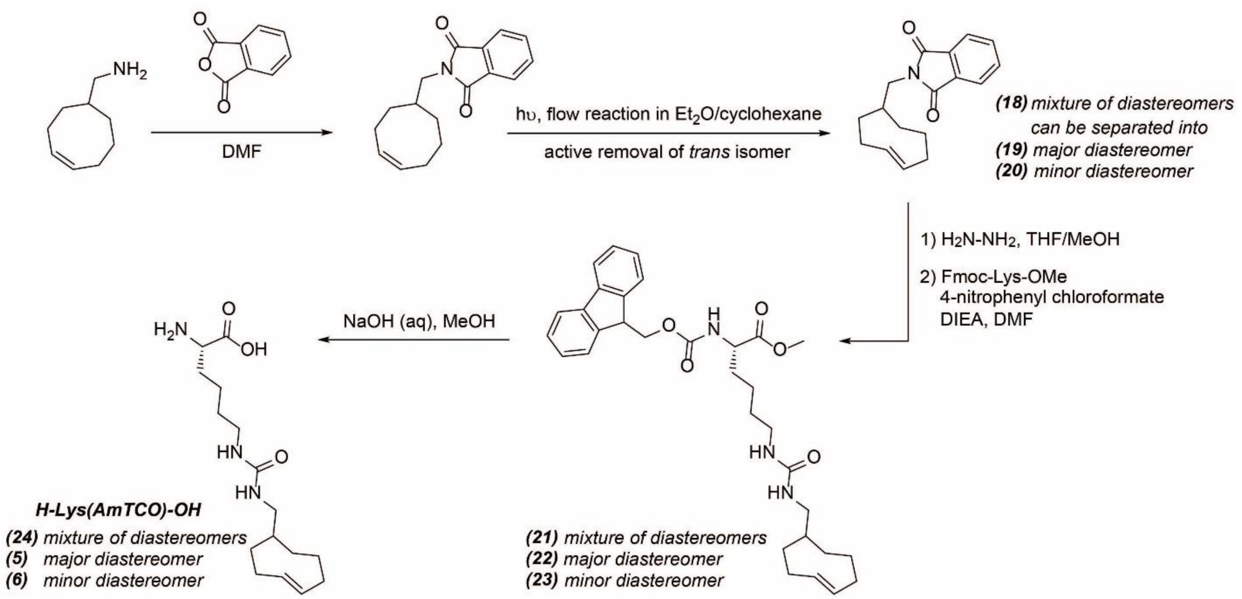

1-(Phthalimidomethyl)-trans-cyclooct-4-ene (18)

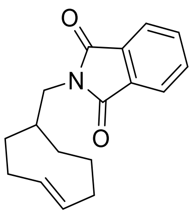

1-(aminomethyl)-*cis*-cyclooct-4-ene hydrochloride was purchased from Enamine and used as supplied. This synthesis is also described in[11]. It was protected with a phthalimide group using phthalic anhydride. 1-(Phthalimidomethyl)-*cis*-cyclooct-4-ene was photoisomerized in a closed-loop flow reactor. 1-(Phthalimidomethyl)-*trans*-cyclooct-4-ene **(18)** was actively trapped on $AgNO_3$-impregnated silica. The **(18)**-$AgNO_3$ complex was eluted from the trapping column, dissociated with ammonia and the product was purified by flash chromatography. It was obtained as a mixture of two diastereomers (7/3 ratio as determined by UPLC) that can be regarded as axial and equatorial diastereomers. ^{1}H NMR (400 MHz, $CDCl_3$) δ 7.86-7.82 (m, 2H), 7.74-7.70

(m, 2H), 5.76-5.45 (m, 2H), 3.84-3.39 (m, 2H), 2.55-1.06 (m, 11H). LC/MS (ESI): t_R = 3.15 min (major diastereomer) and 3.23 min (minor diastereomer), m/z 270.2 $[M+H]^+$. HRMS of major diatereomer (ESI): m/z 270.1490 $[M+H]^+$; calculated for $C_{17}H_{20}NO_2$, 270.1494. HRMS of minor diatereomer (ESI): m/z 270.1491 $[M+H]^+$; calculated for $C_{17}H_{20}NO_2$, 270.1494

Part of the mixture of diastereomers **(18)** (200 mg) was separated by liquid chromatography to yield the major diastereomer **(19)** (82 mg, 59% yield, >99% purity) and the minor diastereomer **(20)** (30 mg, 50% yield, 95% purity) as transparent oils. The separation was performed by the company SiChem. ^{1}H NMR of major diastereomer **(19)** (400 MHz, $CDCl_3$) δ 7.84 (dd, *J* = 5.4, 3.0 Hz, 2H), 7.72 (dd, *J* = 5.4, 3.0 Hz, 2H), 5.52 (qdd, *J* = 16.0, 10.5, 3.4 Hz, 2H), 3.53-3.36 (m, 2H), 2.43-2.30 (m, 2H), 2.12-1.80 (m, 3H), 1.76 (dd, *J* = 13.1, 4.3 Hz, 1H), 1.70-1.60 (m, 1H), 1.54-1.29 (m, 3H), 1.09 (ddd, *J* = 15.6, 12.1, 4.2 Hz, 1H). LC/MS (ESI) of major diastereomer **(22)**: t_R = 3.12 min, m/z 270.1 $[M+H]^+$. ^{1}H NMR of minor diastereomer **(20)** (400 MHz, $CDCl_3$) δ 7.83 (dd, *J* = 5.6, 3.0 Hz, 2H), 7.71 (dd, *J* = 5.6, 3.0 Hz, 2H), 5.72 (ddd, *J* = 16.0, 10.7, 3.7 Hz, 1H), 5.57 (ddd, *J* = 16.0, 10.7, 3.7 Hz, 1H), 3.81 (dd, *J* = 13.5, 10.9 Hz, 1H), 3.57 (dd, J = 13.5, 5.2 Hz, 1H), 2.50 (qd, *J* = 12.0, 4.4 Hz, 1H), 2.37 (tt, *J* = 9.1, 4.5 Hz, 1H), 2.33-2.24 (m, 1H), 2.17-1.99 (m, 2H), 1.98-1.79 (m, 3H), 1.78-1.65 (m, 1H), 1.66-1.52 (m, 1H), 1.28 (dd, *J* = 15.4, 12.3 Hz, 1H). LC/MS (ESI) of minor diastereomer **(23)**: tR = 3.19 min, m/z 270.1 $[M+H]^+$.

Fmoc-Lys(AmTCO)-OMe (21)

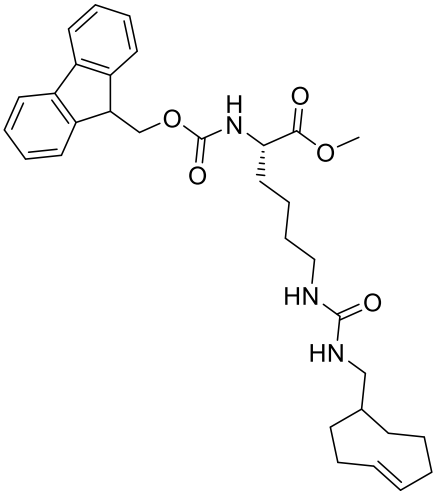

The mixture of diastereomers **(18)** (50 mg, 0.186 mmol, 1 eq) was dissolved in THF (800 µL) and methanol (800 µL). Hydrazine monohydrate (180 µL, 3.71 mmol, 20 eq) was added. The reaction was stirred at 60 °C for 1 h. Then it was concentrated under reduced pressure and hydrazine was co-evaporated with methanol. 1-(Aminomethyl)-*trans*-cyclooct-4-ene was obtained as a white powder.

Fmoc-Lys-OMe.HCl (128 mg, 0.306 mmol, 1.6 eq) was dissolved in DMF (1 mL) in the presence of DIEA (324 µL, 1.85 mmol, 10 eq). 4-Nitrophenyl chloroformate (75 mg, 0.372 mmol, 2 eq) was added into the Fmoc-Lys-OMe solution at 0 °C. The reaction mixture was kept at 0 °C for 10 min and then added onto 1-(aminomethyl)-*trans*-cyclooct-4-ene. The reaction was stirred at RT for 45 min. The resulting mixture was diluted with water and extracted three times with DCM. Organic layers were combined and concentrated. The product was purified by prep HPLC and freeze-dried to yield the mixture of diastereomers **(21)** as a white powder (21 mg, 21% yield, 84% purity). LC/MS (ESI): tR = 3.12 min, m/z 548.4 $[M+H]^+$.

Diastereomers **(22)** (28 mg, 63% yield, 75% purity) and **(23)** (11 mg, 24% yield, 86% purity) were synthesized following the same protocol, starting from diastereomers **(19)** (22 mg, 0.082 mmol) and **(20)** (22 mg, 0.082 mmol) respectively. LC/MS (ESI) of major diastereomer **(22)**: tR = 3.10 min, m/z 548.4 $[M+H]^+$. LC/MS (ESI) of minor diastereomer **(23)**: tR = 3.11 min, m/z 548.4 $[M+H]^+$.

H-Lys(AmTCO)-OH (24)

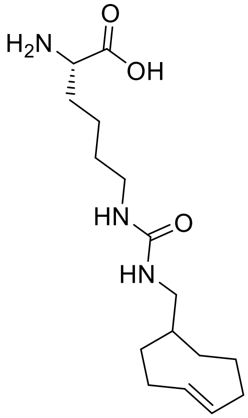

Fmoc-Lys(AmTCO)-OMe diastereomers **(21)** (21 mg, 0.039 mmol, 1 eq) were dissolved in methanol (2 mL). 0.2 M aqueous sodium hydroxide (600 µl, 0.120 mmol, 3 eq) was added. A precipitate formed upon addition but disappeared quickly with stirring. The reaction mixture was stirred at RT overnight. Methanol was removed under reduced pressure and the residue was purified by reverse phase flash chromatography (SNAP Ultra C18 12 g column, gradient from 0 to 30% acetonitrile in water in 15 column volumes). Pure fractions were freeze-dried to yield H-Lys(AmTCO)-OH **(24)** as a white powder (11 mg, 87% yield, >95% purity by NMR). ^{1}H NMR (400 MHz, D_2O) δ 5.76 – 5.44 (m, 2H), 3.69 (t, *J* = 6.1 Hz, 1H), 3.22 – 2.72 (m, 4H), 2.40 – 2.29 (m, 1H), 2.22 – 1.59 (m, 8H), 1.58 – 1.08 (m, 7H), 1.08 – 0.93 (m, 1H). LC/MS (ESI): tR = 1.40 min, m/z 312.3 $[M+H]^+$. HRMS (ESI): m/z 312.2291 $[M+H]^+$; calculated for $C_{16}H_{30}N_3O_3$, 312.2287.

Diastereomers **(5)** (7 mg, 43% yield, >95% purity by NMR) and **(6)** (4 mg, 64% yield, >95% purity by NMR) were synthesized following the same protocol, starting from diastereomers **(22)** (28 mg, 0.051 mmol) and **(23)** (11 mg, 0.020 mmol) respectively. ^{1}H NMR of major diastereomer **(5)** (400 MHz, D_2O) δ 5.74 – 5.43 (m, 2H), 3.64 (t, *J* = 6.2 Hz, 1H), 3.08 (t, *J* = 6.8 Hz, 2H), 2.84 (qd, *J* = 13.3, 7.5 Hz, 2H), 2.40 – 2.30 (m, 2H), 2.09 – 1.68 (m, 6H), 1.55 – 1.27 (m, 7H), 1.21 – 1.11 (m, 1H), 1.07 – 0.95 (m, 1H). ^{13}C NMR of major diastereomer **(5)** (101 MHz, D_2O) δ 175.82 (C), 160.70 (C), 135.19 (CH), 134.09 (CH), 54.75 (CH), 46.97 (CH_2), 41.86 (CH), 39.33 (CH_2), 38.45 (CH_2), 34.81 (CH_2), 34.48 (CH_2), 33.16 (CH_2), 33.03 (CH_2), 30.53 (CH_2), 28.88 (CH_2), 21.65 (CH_2). LC/MS (ESI) of major diastereomer **(5)**: tR = 1.41 min, m/z 312.2 $[M+H]^+$. HRMS (ESI) of major diastereomer **(5)**: m/z 312.2285 $[M+H]^+$; calculated for $C_{16}H_{30}N_3O_3$, 312.2287.

^{1}H NMR of minor diastereomer **(6)** (400 MHz, D_2O) δ 5.71 – 5.48 (m, 2H), 3.67 (t, *J* = 6.1 Hz, 1H), 3.21 – 3.01 (m, 4H), 2.22 – 1.72 (m, 10H), 1.71 – 1.59 (m, 1H), 1.59 – 1.41 (m, 3H), 1.44 – 1.28 (m, 2H), 1.20 (t, *J* = 13.5 Hz, 1H). ^{13}C NMR of minor diastereomer **(6)** (101 MHz, D_2O) δ 175.25 (C), 160.61 (C), 134.90 (CH), 134.25 (CH), 54.75 (CH), 41.16 (CH_2), 39.39 (CH_2), 38.16 (CH_2), 34.61 (CH_2), 34.29 (CH), 30.34 (CH_2), 30.31 (CH_2), 30.17 (CH_2), 30.07 (CH_2), 28.99 (CH_2), 21.67 (CH_2). LC/MS (ESI) of minor diastereomer **(6)**: tR = 1.39 min, m/z 312.3 $[M+H]^+$. HRMS (ESI) of minor diastereomer **(6)**: m/z 312.2285 $[M+H]^+$; calculated for $C_{16}H_{30}N_3O_3$, 312.2287.

NMR analyses

^{1}H NMR spectrum (400 MHz) of the mixture of diastereomers **(18)** in $CDCl_3$

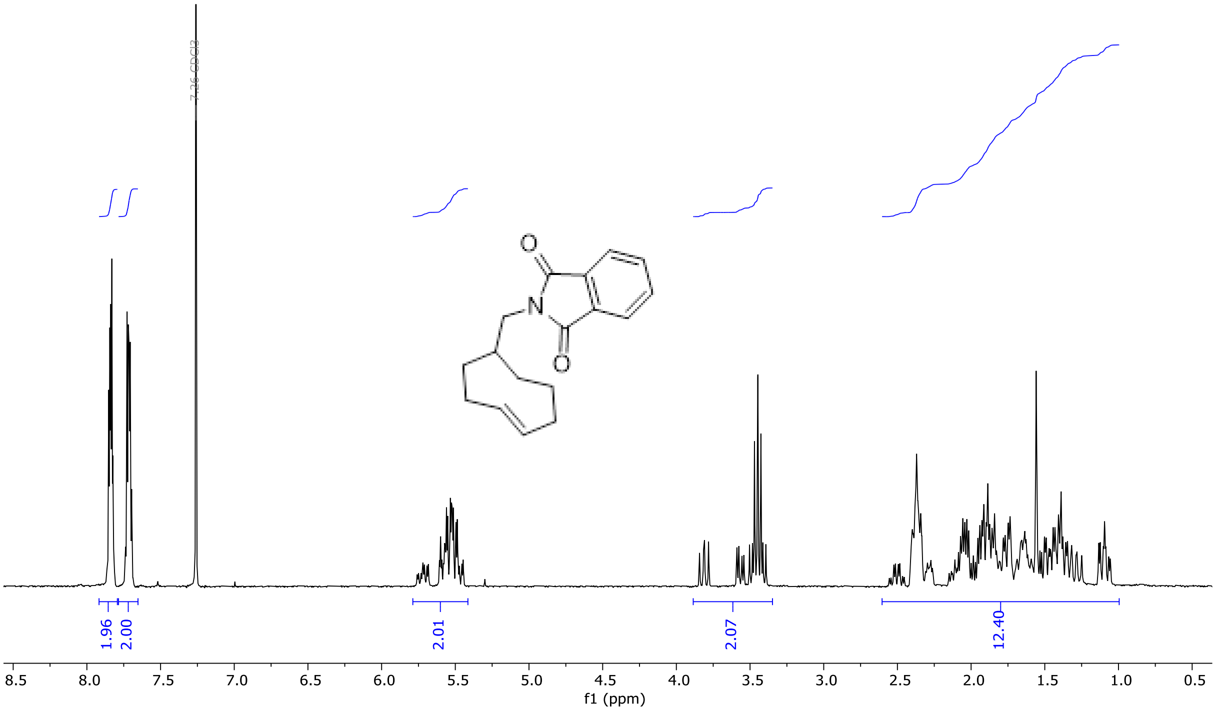

^{13}C NMR spectrum (101 MHz) of the mixture of diastereomers **(18)** in $CDCl_3$

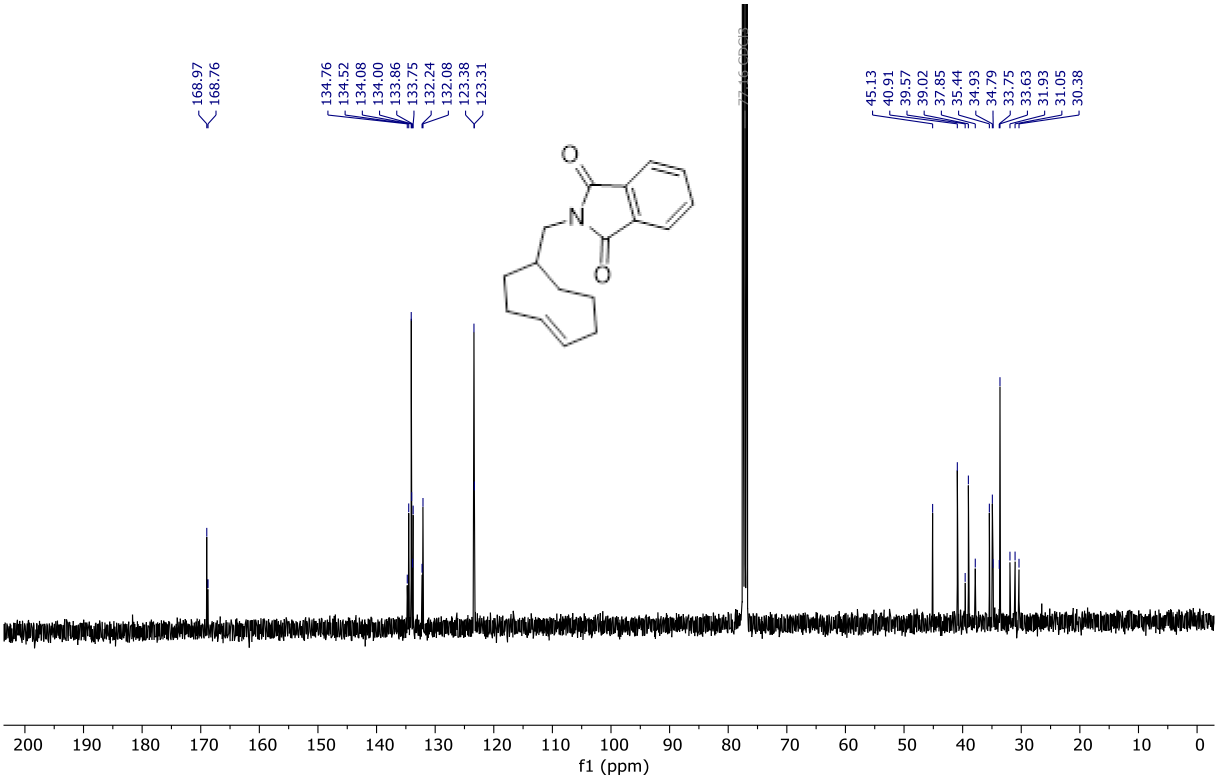

^{1}H NMR spectrum (400 MHz) of the major diastereomer **(19)** in $CDCl_3$

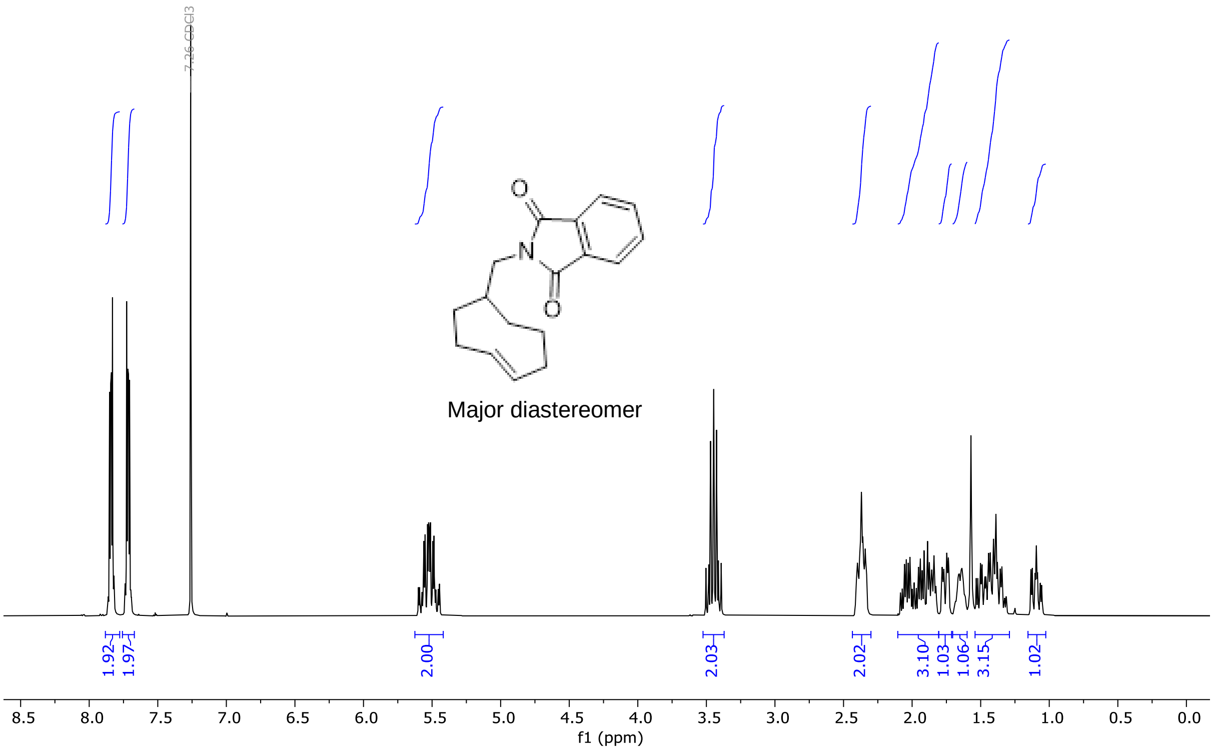

^{13}C NMR spectrum (101 MHz) of the major diastereomer **(19)** in $CDCl_3$

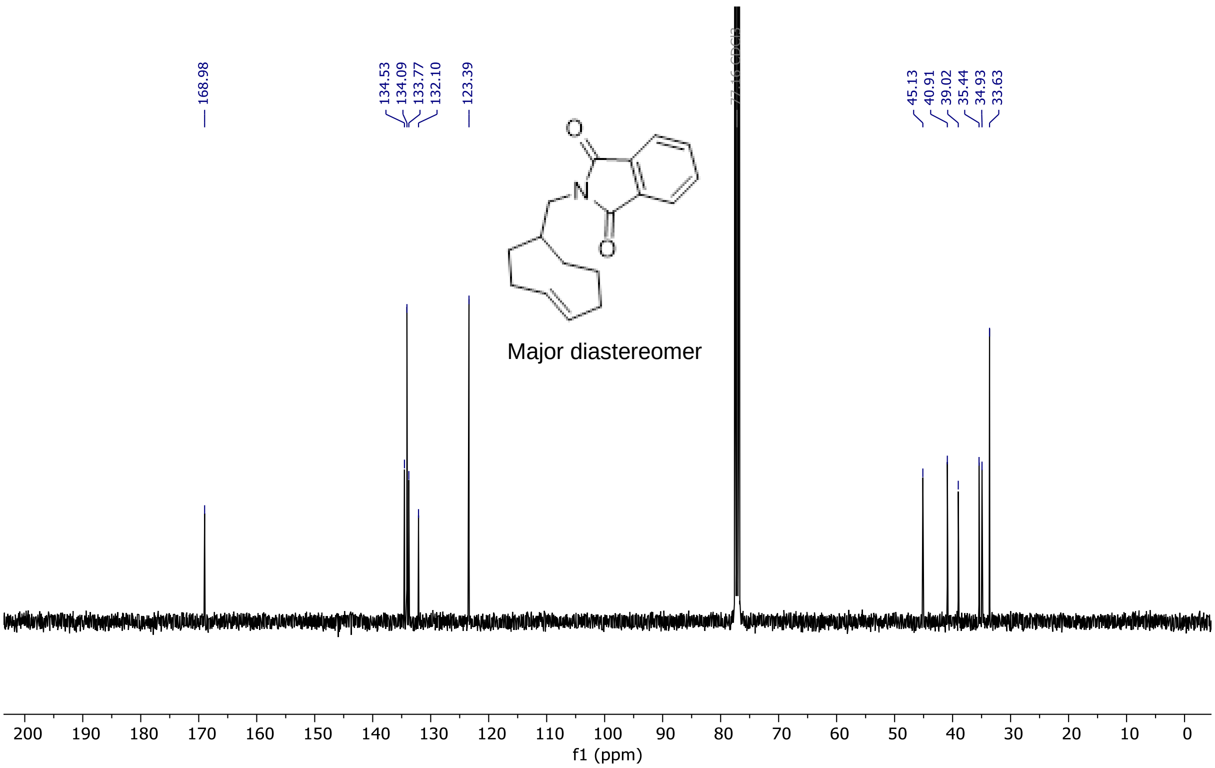

^{1}H NMR spectrum (400 MHz) of the minor diastereomer **(20)** in $CDCl_3$

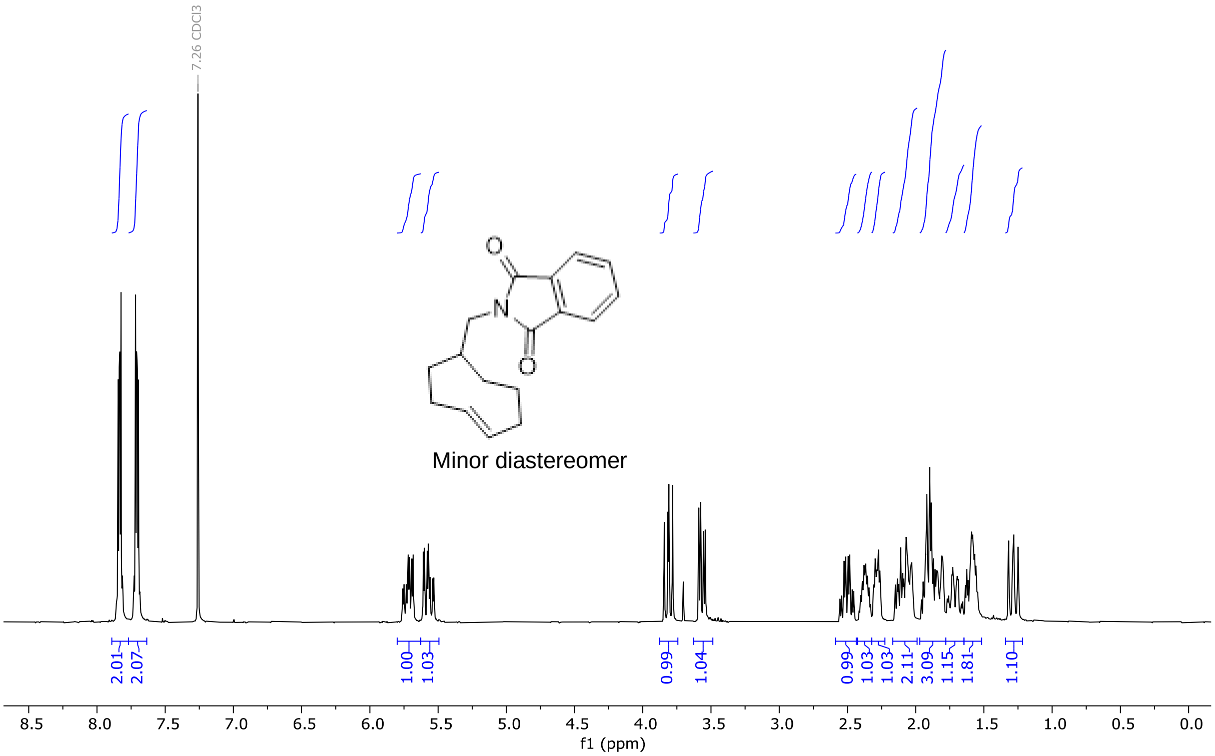

^{13}C NMR spectrum (101 MHz) of the minor diastereomer **(20)** in $CDCl_3$

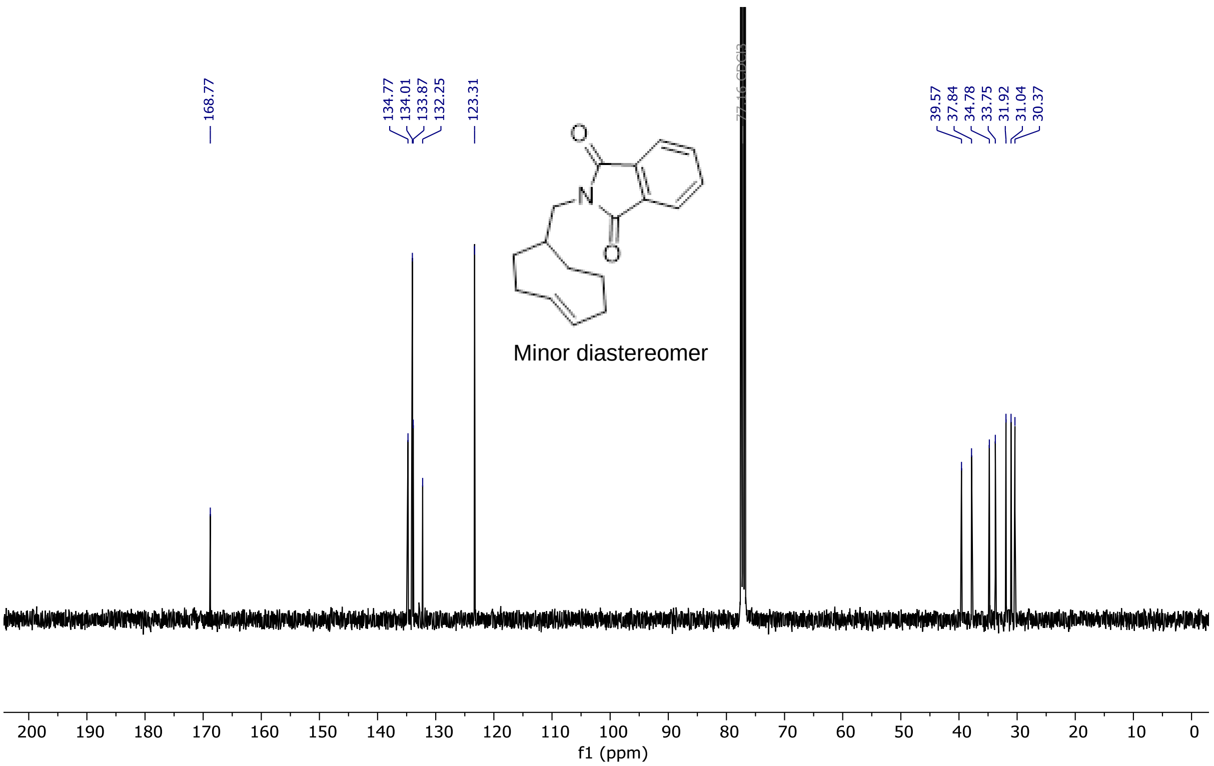

^{1}H NMR spectrum (400 MHz) of the mixture of diastereomers **(24)** in D_2O

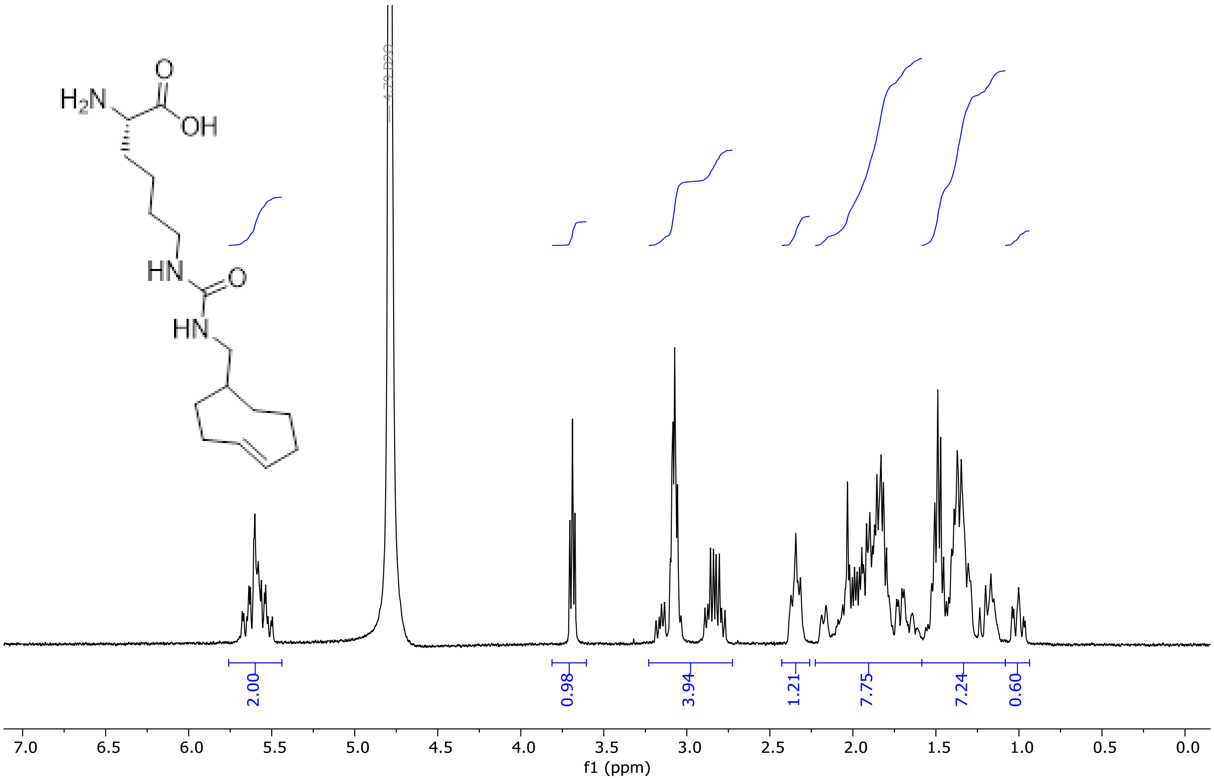

^{13}C NMR spectrum (101 MHz) of the mixture of diastereomers **(24)** in D_2O

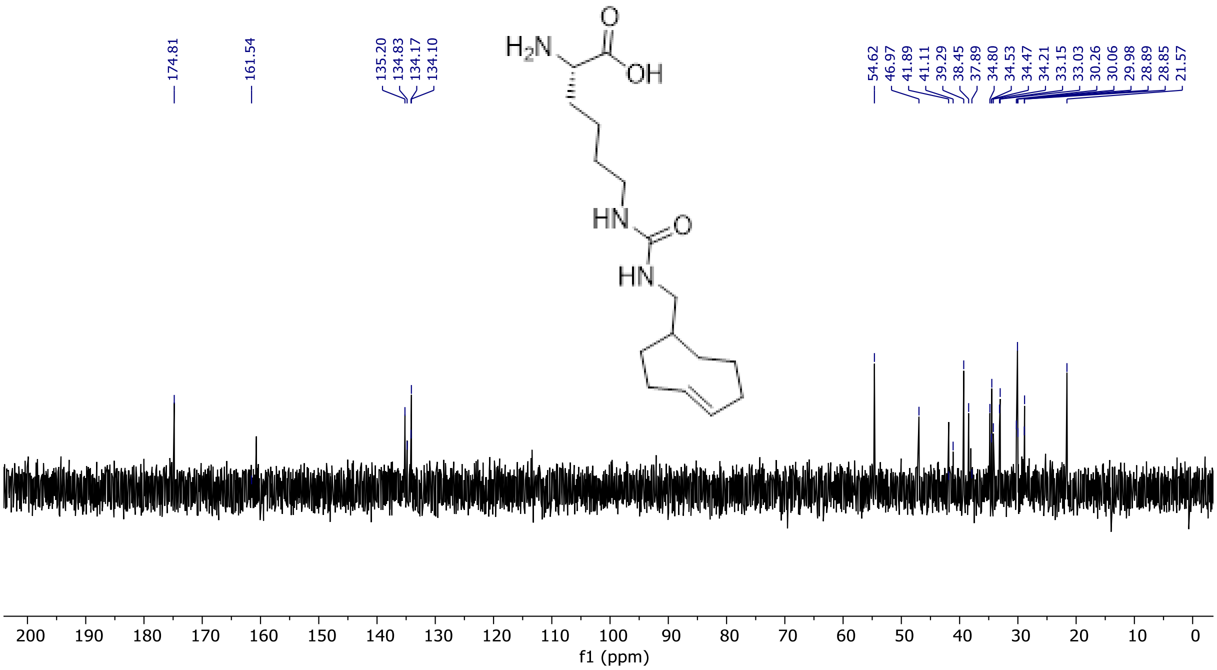

^{1}H NMR spectrum (400 MHz) of the major diastereomer **(5)** in D_2O

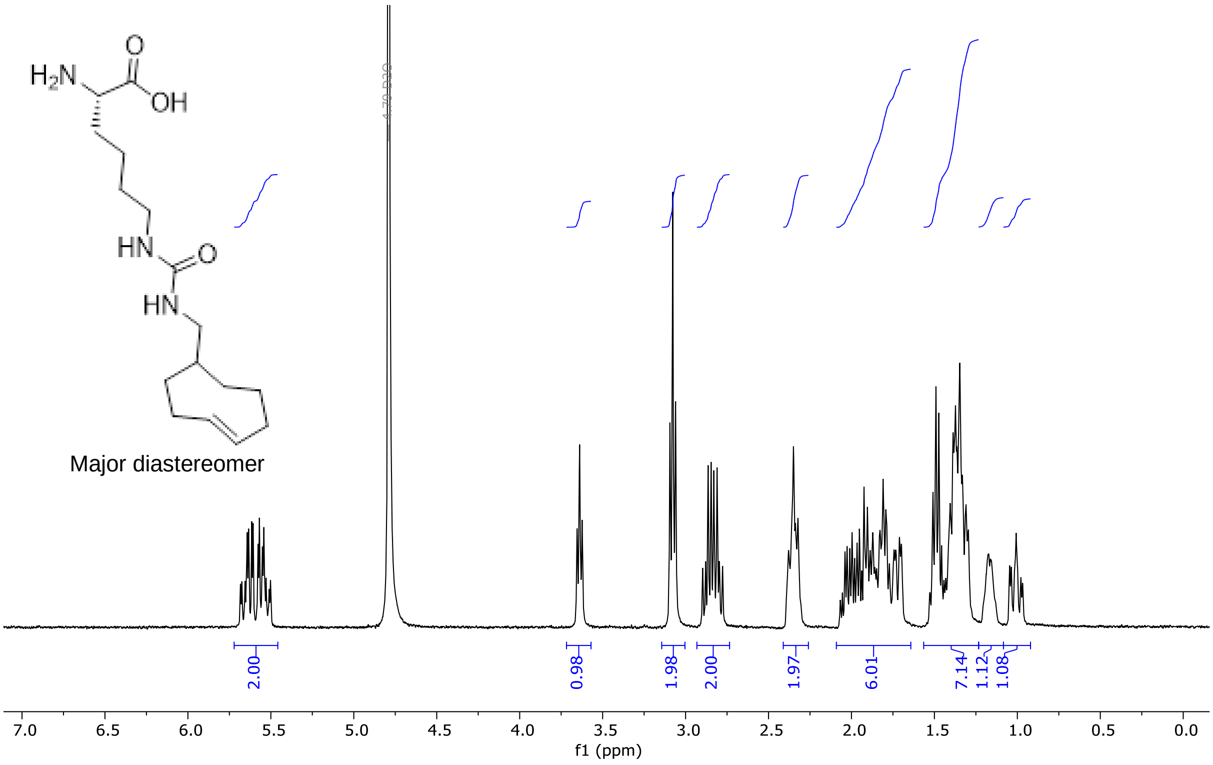

^{13}C NMR spectrum (101 MHz) of the major diastereomer **(5)** in D_2O

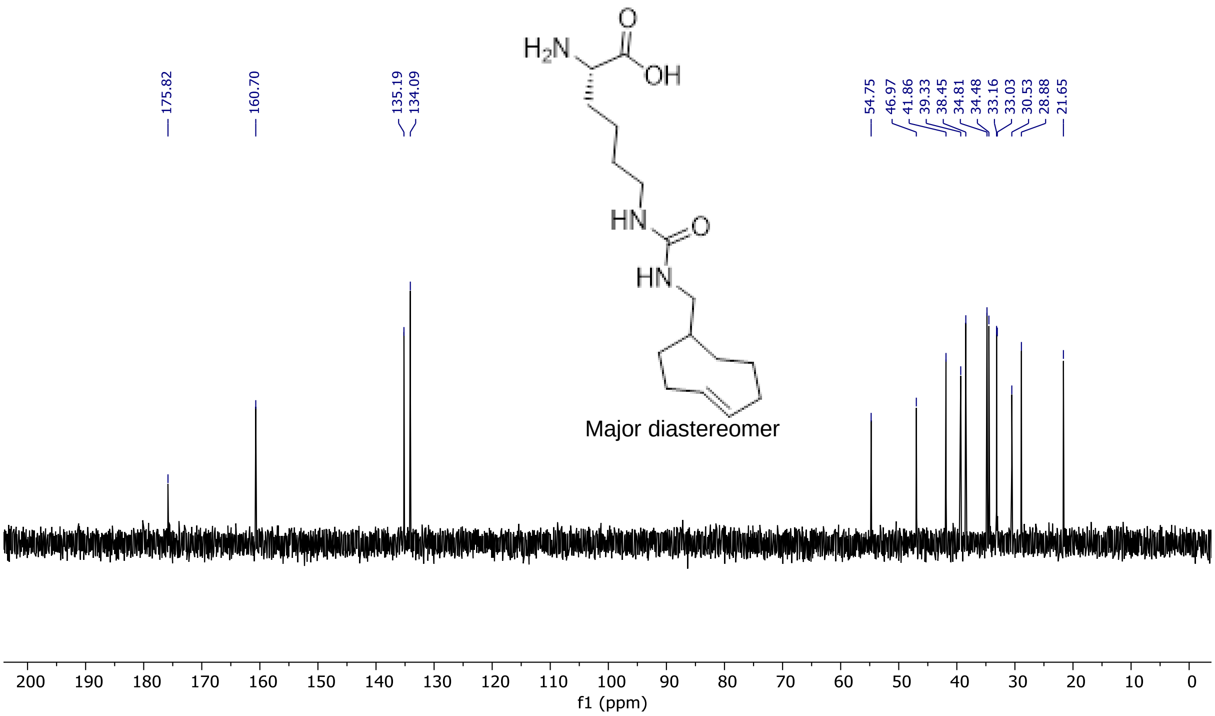

^{1}H NMR spectrum (400 MHz) of the minor diastereomer **(6)** in D_2O

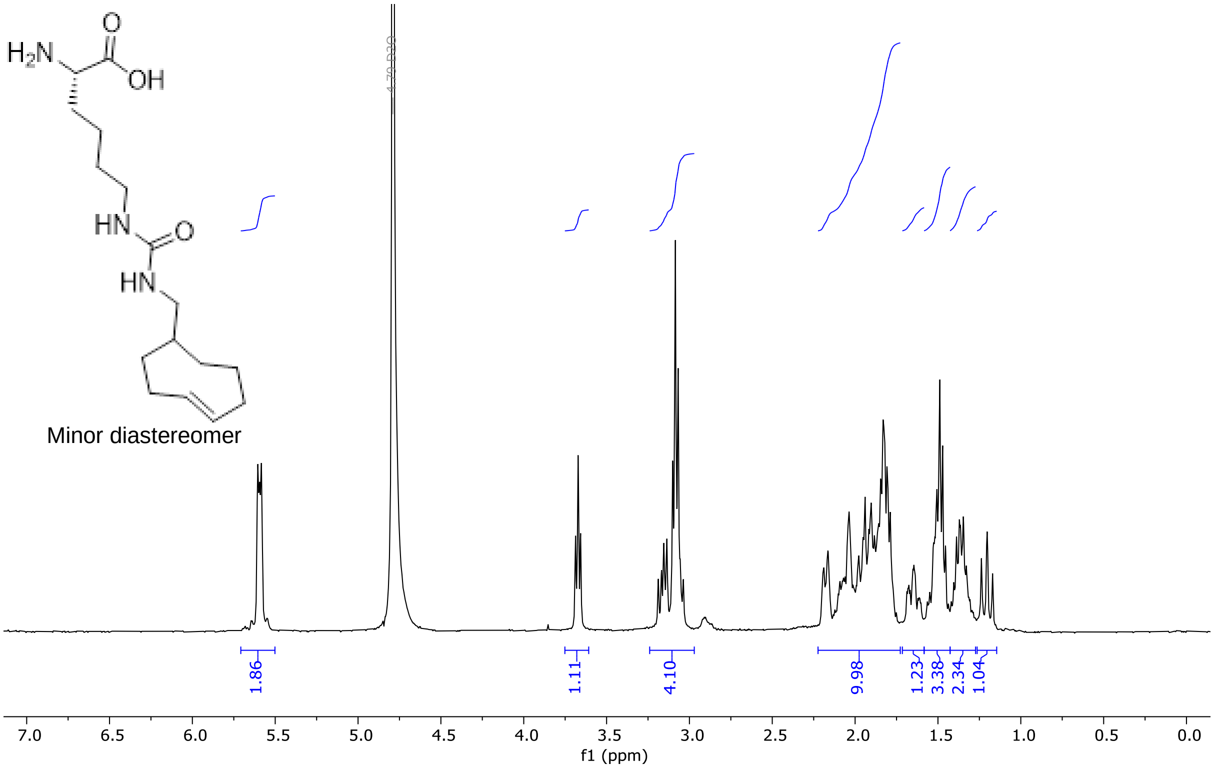

^{13}C NMR spectrum (101 MHz) of the minor diastereomer **(6)** in D_2O

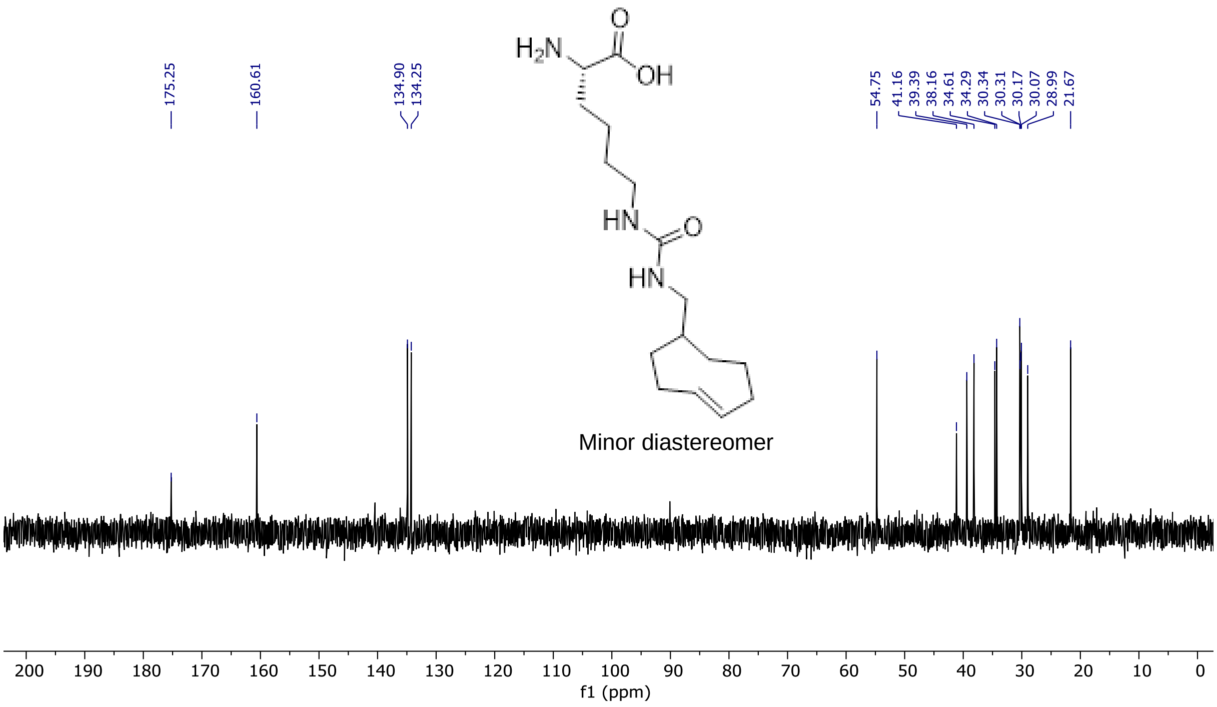

Supporting figures

Figure S1.: Comparison of the incorporation efficiency of different ncAA. The plot shows the incorporation efficiency versus different concentrations of ncAAs. To this end, HEK293T cells transiently expressing iRFP-EGFP^{Y39TAG}, tRNA^{Pyl} and PylRS (Y306A, Y384F mutant unless otherwise indicated) were grown for 20 hours in DMEM containing different ncAAs at the indicated concentrations before they were analyzed via FFC. Transfected cells were identified by fluorescence and the geometric mean of the EGFP signal and the geometric mean of the iRFP signal were calculated. The plot shows the ratio of the geometric mean of the EGFP signal divided by the geometric mean of the iRFP signal of the different ncAAs (as indicated by the different colors) plotted against the ncAA concentration. The grey dashed line indicates the concentration of each ncAA gaining the same iRFP-EGFP^{Y39ncAA} expression level, which was then used for the *in cellulo* FRET kinetics study.

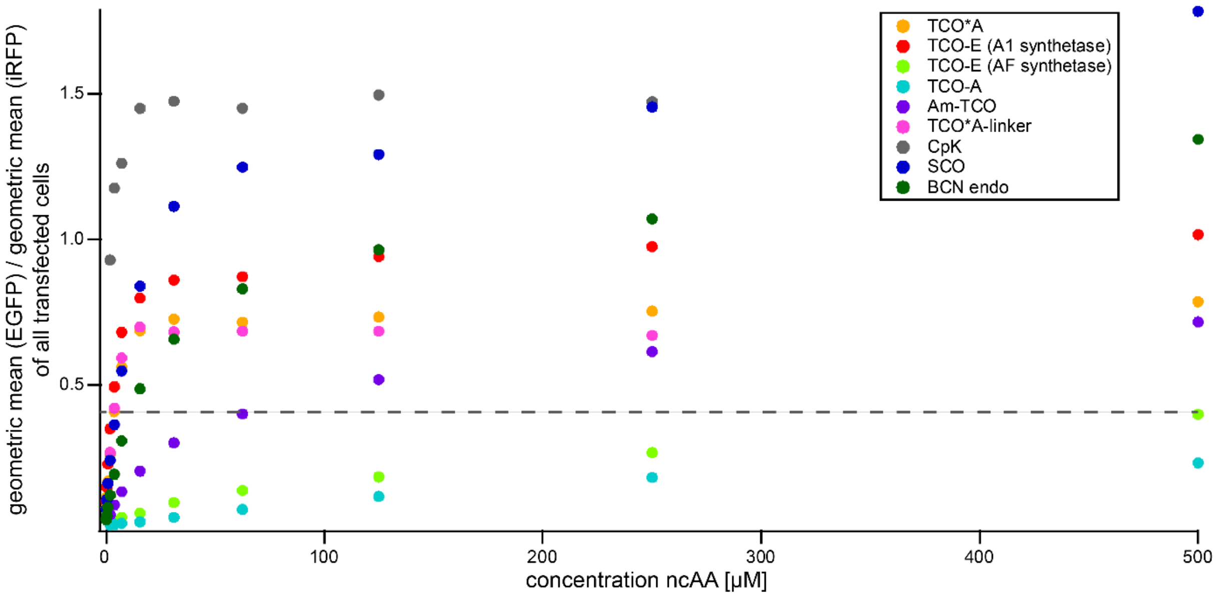

Figure S2. *In cellulo* FRET FFC data, kinetics and reaction rate constant determination of TCO*-A, TCO-E, BCN, SCO and AmTCO-E. Shown are the raw data of the *in cellulo* FRET FFC analysis for the different compounds as described in the supplementary methods. In brief, HEK293T cells expressing EGFP with the respective ncAA were labeled with the indicated concentration of SiR-tet and analyzed via FFC after indicated time points. The FFC plots for a) TCO*-A, b) TCO-E, c) BCN, d) SCO and e) AmTCO-E show the FRET signal [A.U.] over the GFP fluorescence signal [A.U.] for different time points (0-300 minutes) of a representative experiment. f) Kinetic plots for each compound. Each plot contains the data from three different dye concentrations used for the experiment, the highest concentration in cyan, the middle concentration in light blue and the lowest concentration in dark blue. The data points were fitted with an exponential fit using Igor Pro (WaveMetrics), to obtain k_{Obs} for each dye concentration. In the case of SCO, the three exponential fits do not reach the same end points, because the reaction between SCO and the dye (SiR-tet) is very slow and therefore, it is impossible to reach full labeling in the measured time frame. Hence the calculated k_{Obs} represents an upper limit. For AmTCO-E it was not possible to fit the highest concentration data points, because the reaction is very fast and also shows a fast elimination or quenching mechanism, which does not allow robust fitting. Thus this compound was measured again with cells pre incubated on ice (Figure S4). g) The k_{Obs}, resulting from the kinetic plots, were then plotted over the corresponding concentrations for each compound (AmTCO-E in light green, TCO-E in dark green, BCN in cyan, TCO*-A in blue and SCO in black). For all compounds, except for AmTCO-E, the highest and the middle concentrations were plotted. For AmTCO-E only the lowest and the middle concentration were plotted, because the highest concentration could not be fitted. The data points were fitted with a linear fit to obtain the reaction rate constant (k_{On}) for each compound.

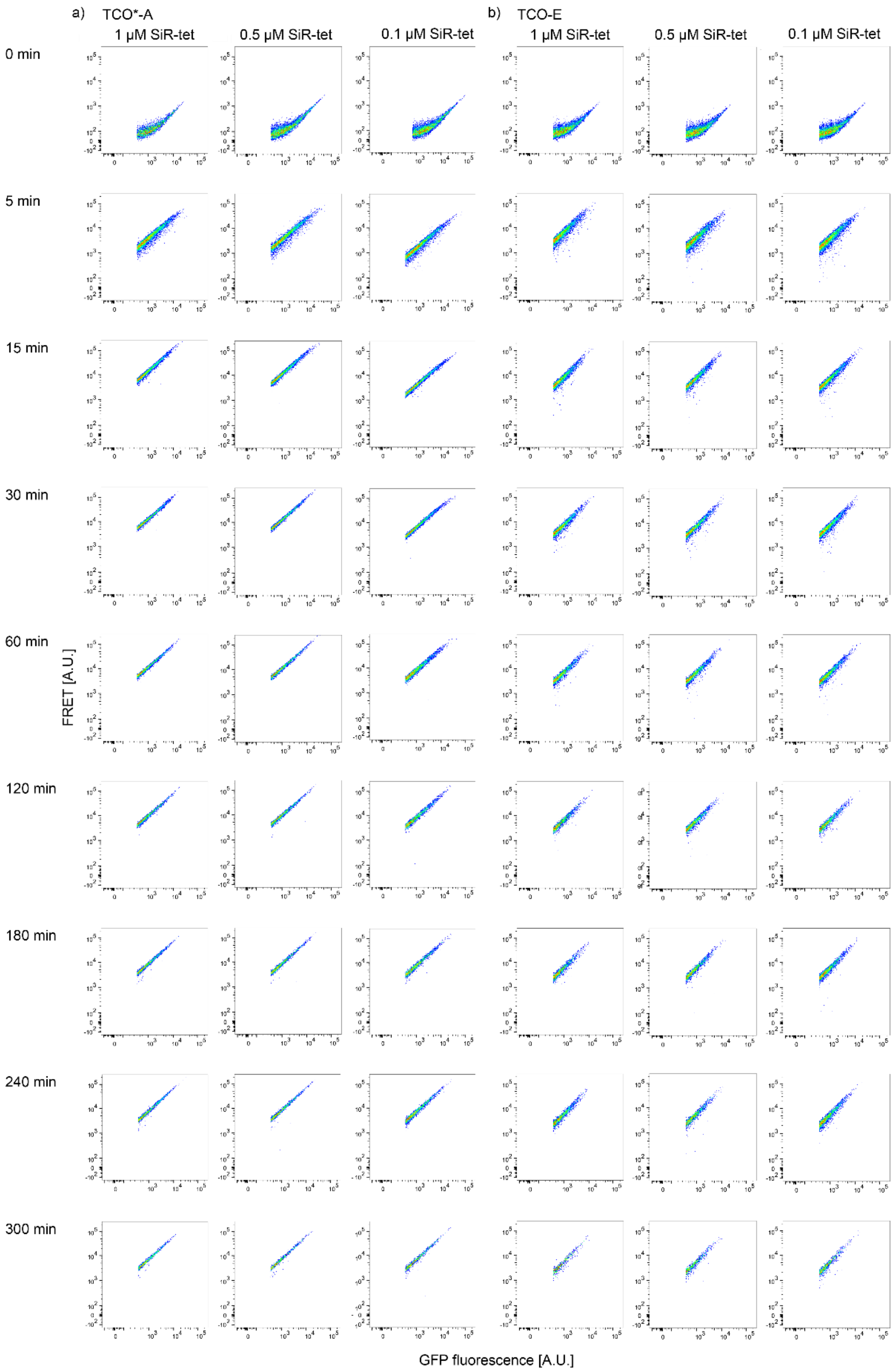

a) TCO*-A
1 µM SiR-tet
0.5 µM SiR-tet
0.1 µM SiR-tet
b) TCO-E
1 µM SiR-tet
0.5 µM SiR-tet
0.1 µM SiR-tet
0 min
5 min
15 min
30 min
60 min
120 min
180 min
240 min
300 min
FRET [A.U.]
GFP fluorescence [A.U.]

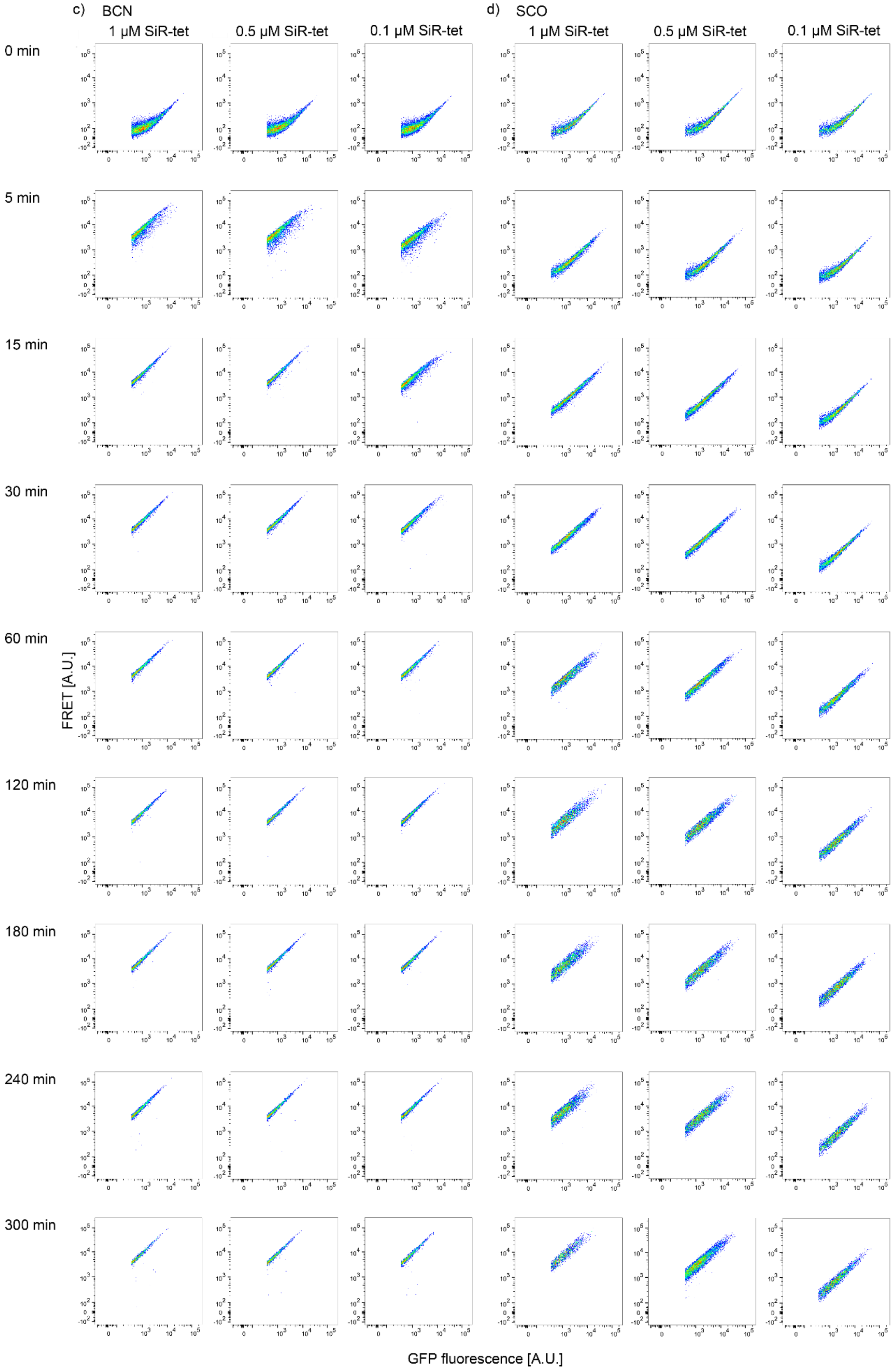
c) BCN
1 µM SiR-tet
0.5 µM SiR-tet
0.1 µM SiR-tet
d) SCO
1 µM SiR-tet
0.5 µM SiR-tet
0.1 µM SiR-tet
0 min
5 min
15 min
30 min
60 min
120 min
180 min
240 min
300 min
FRET [A.U.]
GFP fluorescence [A.U.]

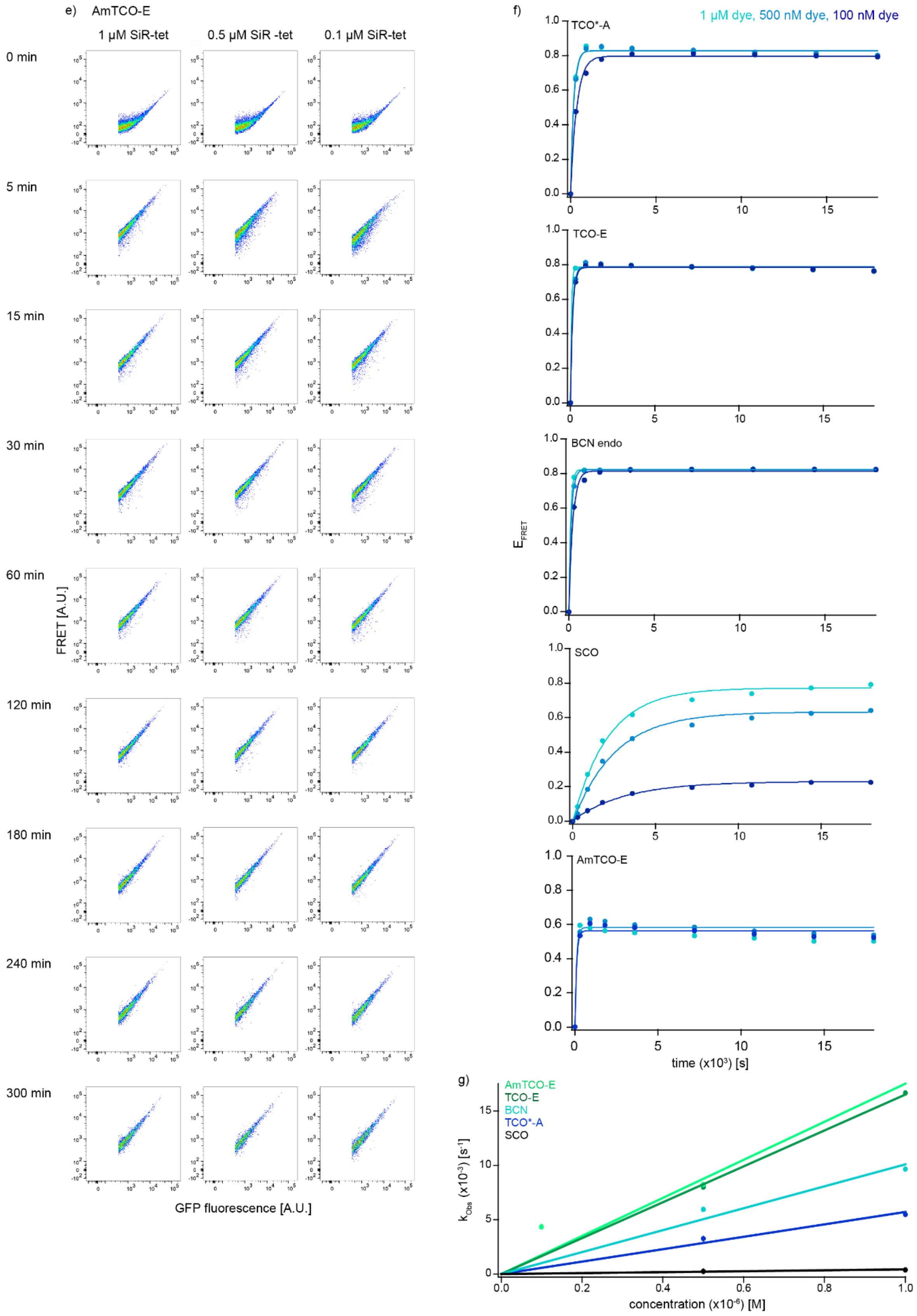
e) AmTCO-E
1 µM SiR-tet
0.5 µM SiR -tet
0.1 µM SiR-tet
0 min
5 min
15 min
30 min
60 min
120 min
180 min
240 min
300 min
FRET [A.U.]
GFP fluorescence [A.U.]
f)
1 µM dye, 500 nM dye, 100 nM dye
TCO*-A
TCO-E
BCN endo
SCO
AmTCO-E
E_{FRET}
time (x10³) [s]
g)
AmTCO-E
TCO-E
BCN
TCO*-A
SCO
k_{Obs} (x10⁻³) [s⁻¹]
concentration (x10⁻⁶) [M]

Figure S3. *In cellulo* FRET FFC data, kinetics and reaction rate constant determination for AmTCO-A, TCO*N, TCO*C-E, TCO*linker-A and CpK. Shown are the raw data of the *in cellulo* FRET FFC analysis for the different compounds as described in the supplementary methods. In brief, HEK293T cells expressing EGFP with the respective ncAA were labeled with the indicated concentration of SiR-tet and analyzed via FFC after indicated time points. The FFC plots for a) AmTCO-A, b) TCO*N, c) TCO*C-E, d) TCO*linker-A and e) CpK show the FRET signal [A.U.] over the GFP fluorescence signal [A.U.] for different time points (0-300 minutes) of a representative experiment. f) Kinetic plots for each compound. Each plot contains the data from the three different dye concentrations used for the experiment, the highest concentration in cyan, the middle concentration in light blue and the lowest concentration in dark blue. The data points were fitted with an exponential fit using Igor Pro (WaveMetrics), to obtain k_{Obs} for each dye concentration. g) The k_{Obs}, resulting from the kinetic plots, were then plotted over the corresponding concentrations for each compound (AmTCO-A in green, TCO*N in purple, CpK in light blue, TCO*linker-A in blue and TCO*C-E in magenta). For all compounds, except for AmTCO-A, the highest and the middle concentrations were plotted. For AmTCO-A only the lowest and the middle concentration were plotted, because the highest concentration was too fast to be fitted. The data points were fitted with a linear fit to obtain the reaction rate constant (k_{On}) for each compound. h) Bar graph showing the measured *in vivo* reaction rates for the ncAAs. Bar graph showing the measured relative $E_{FRET\text{-}MAX}$ values for the ncAAs as well as the $E_{FRET\text{-}Final}$ (relative E_{FRET} after 4 hours). These measurements were performed once.

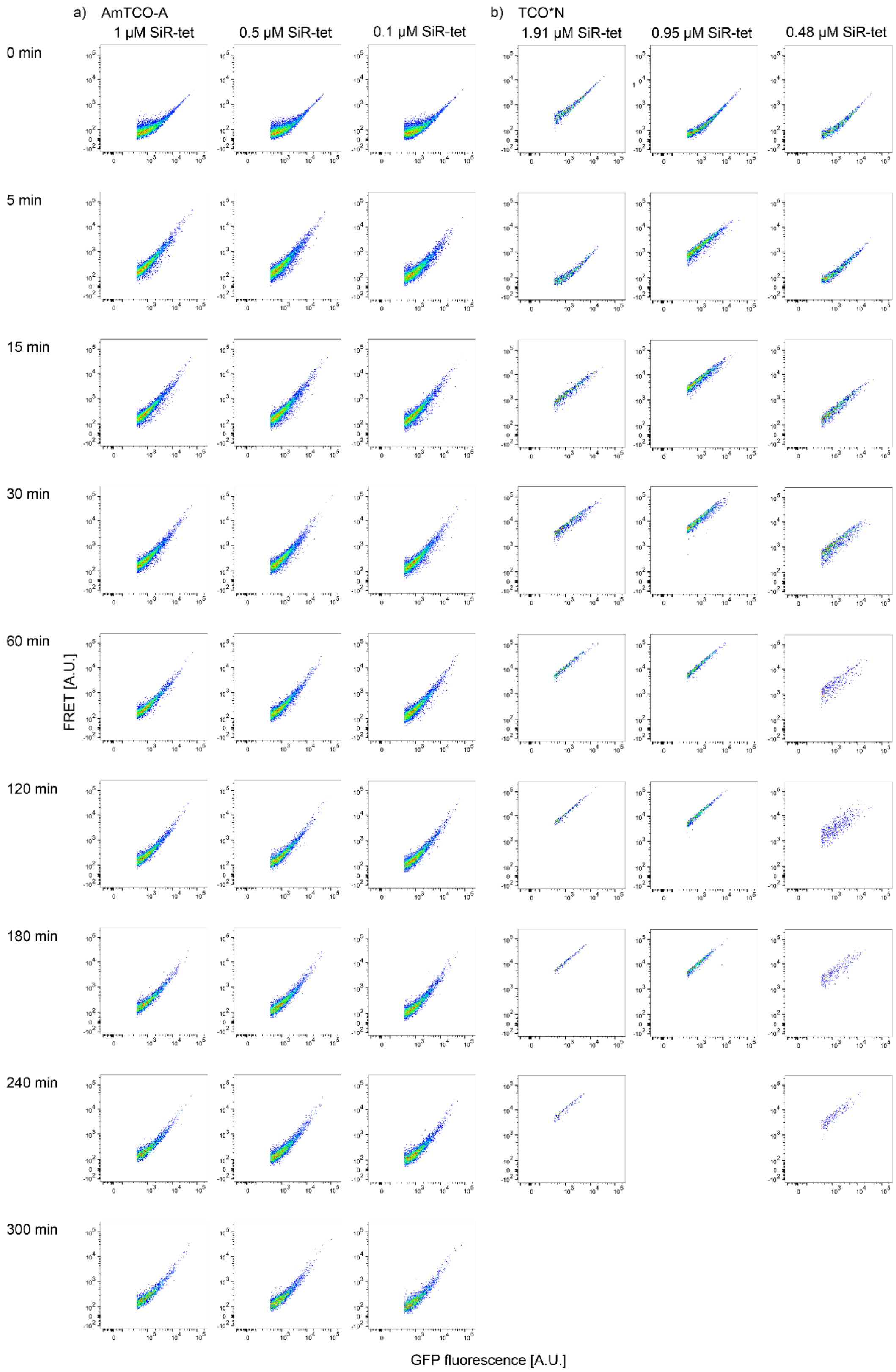
a) AmTCO-A
1 µM SiR-tet
0.5 µM SiR-tet
0.1 µM SiR-tet
b) TCO*N
1.91 µM SiR-tet
0.95 µM SiR-tet
0.48 µM SiR-tet
0 min
5 min
15 min
30 min
60 min
120 min
180 min
240 min
300 min
FRET [A.U.]
GFP fluorescence [A.U.]

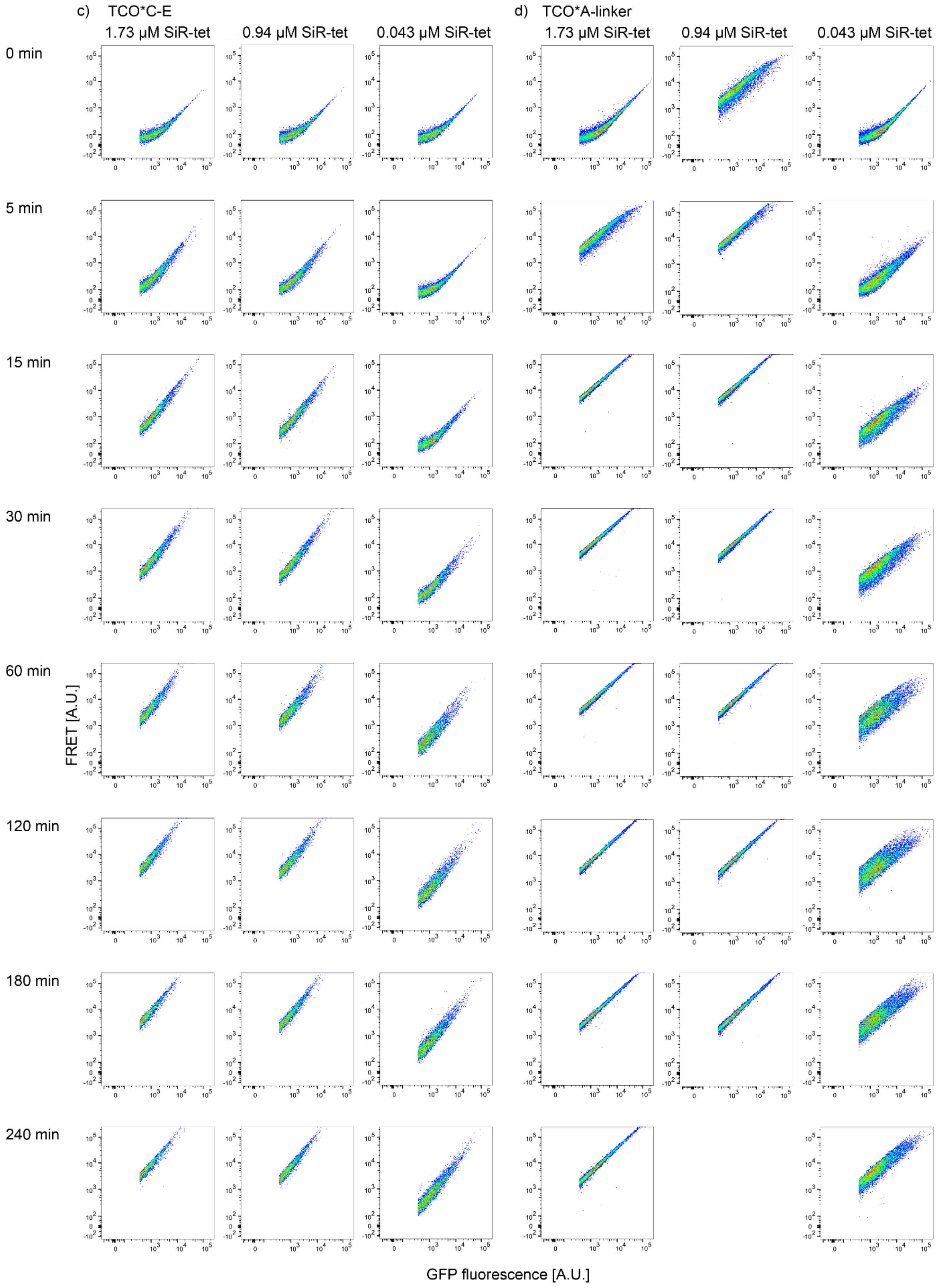

c) TCO*C-E
d) TCO*A-linker
1.73 µM SiR-tet
0.94 µM SiR-tet
0.043 µM SiR-tet
0 min
5 min
15 min
30 min
60 min
120 min
180 min
240 min
FRET [A.U.]
GFP fluorescence [A.U.]

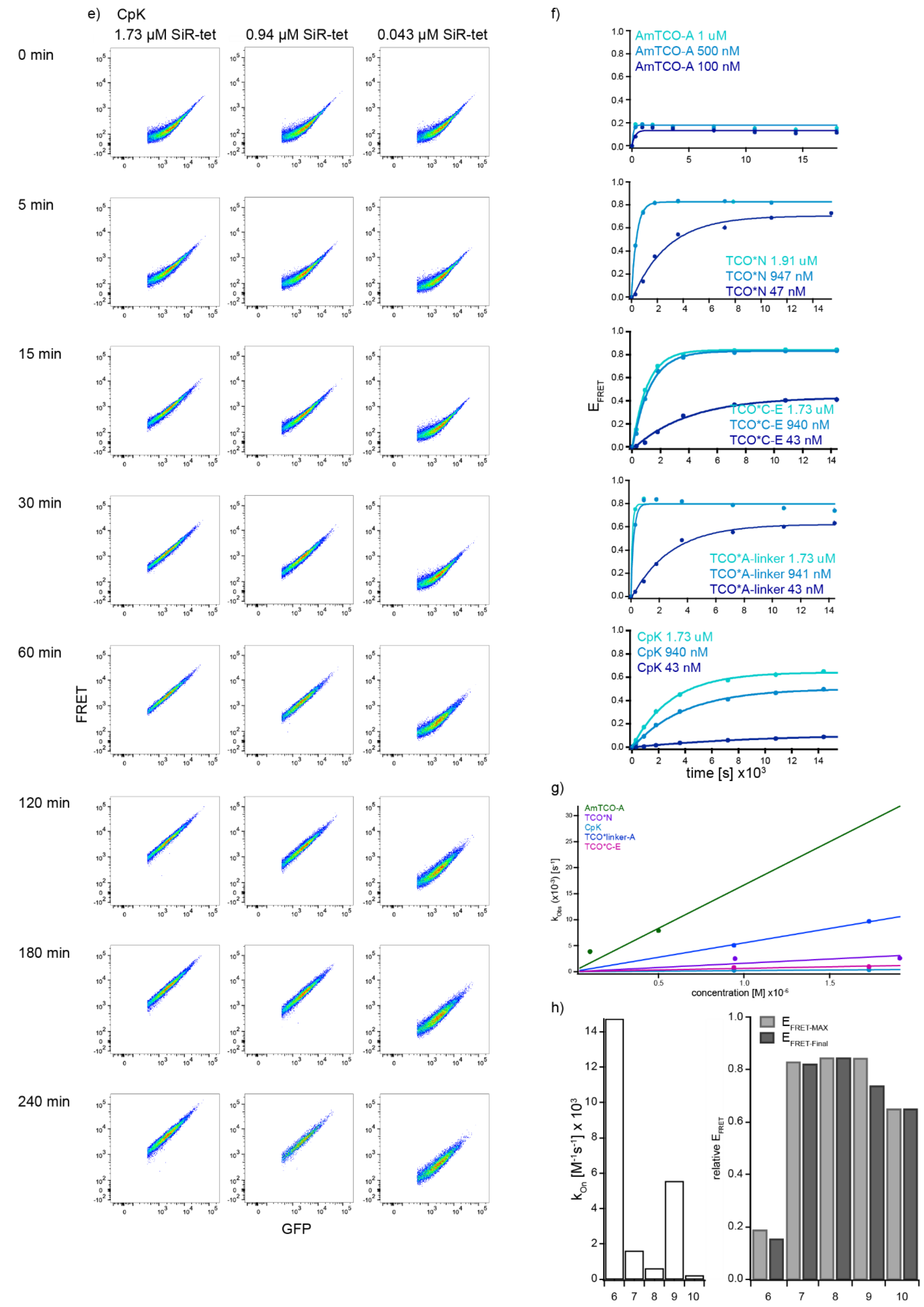

e) CpK
1.73 µM SiR-tet
0.94 µM SiR-tet
0.043 µM SiR-tet
0 min
5 min
15 min
30 min
60 min
120 min
180 min
240 min
FRET
GFP
f)
AmTCO-A 1 uM
AmTCO-A 500 nM
AmTCO-A 100 nM
TCO*N 1.91 uM
TCO*N 947 nM
TCO*N 47 nM
TCO*C-E 1.73 uM
TCO*C-E 940 nM
TCO*C-E 43 nM
TCO*A-linker 1.73 uM
TCO*A-linker 941 nM
TCO*A-linker 43 nM
CpK 1.73 uM
CpK 940 nM
CpK 43 nM
E_{FRET}
time [s] x10^3
g)
AmTCO-A
TCO*N
CpK
TCO*linker-A
TCO*C-E
k_{Obs} (x10^3) [s^{-1}]
concentration [M] x10^{-6}
h)
k_{On} [M^{-1}s^{-1}] x 10^3
relative E_{FRET}
$E_{FRET\text{-}MAX}$
$E_{FRET\text{-}Final}$

Figure S4. *In cellulo* FRET on ice for TCO*-A, TCO-E and AmTCO-E. Shown are the raw data of the *in cellulo* FRET FFC analysis for the different compounds on ice as described in the supplementary methods. In brief, HEK293T cells expressing EGFP with the respective ncAA were labeled with the indicated concentration of SiR-tet and analyzed via FFC after indicated time points. The FFC plots for a) TCO-E, b) TCO*-A and c) AmTCO-E show the FRET signal [A.U.] over the GFP fluorescence signal [A.U.] for different time points (0-300 minutes) of a representative example. d) Kinetic plots for each compound. Each plot contains the data from the three different dye concentrations used for the experiment, the highest concentration in cyan, the middle concentration in light blue and the lowest concentration in dark blue. The data points were fitted with an exponential fit using Igor Pro (WaveMetrics), to obtain the k_{Obs} for each dye concentration. e) Overlay of the FFC data for each compound for three different time points (0 (in black), 30 (in magenta) and 240 (in cyan) minutes). f) The k_{Obs}, resulting from the kinetic plots, were then plotted over the corresponding concentrations for each compound. The data points were fitted with a linear fit to obtain the reaction rate constant for each compound, k_{On}, which is shown in the bar plot (left panel) for each compound. The relative $E_{FRET\text{-}MAX}$ for each compound is shown in the bar plot on the right panel.

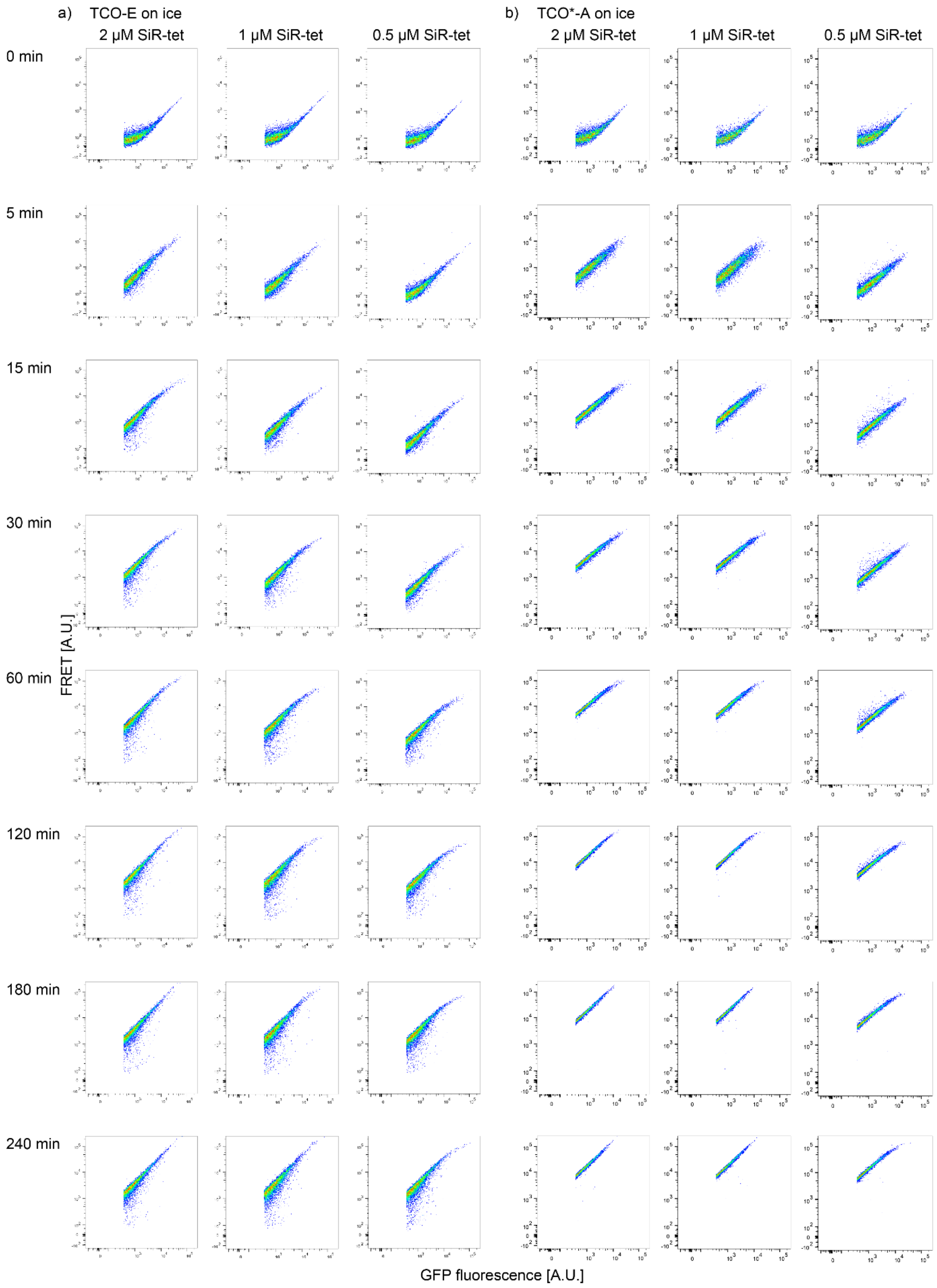
a) TCO-E on ice
2 µM SiR-tet
1 µM SiR-tet
0.5 µM SiR-tet
b) TCO*-A on ice
2 µM SiR-tet
1 µM SiR-tet
0.5 µM SiR-tet
0 min
5 min
15 min
30 min
60 min
120 min
180 min
240 min
FRET [A.U.]
GFP fluorescence [A.U.]

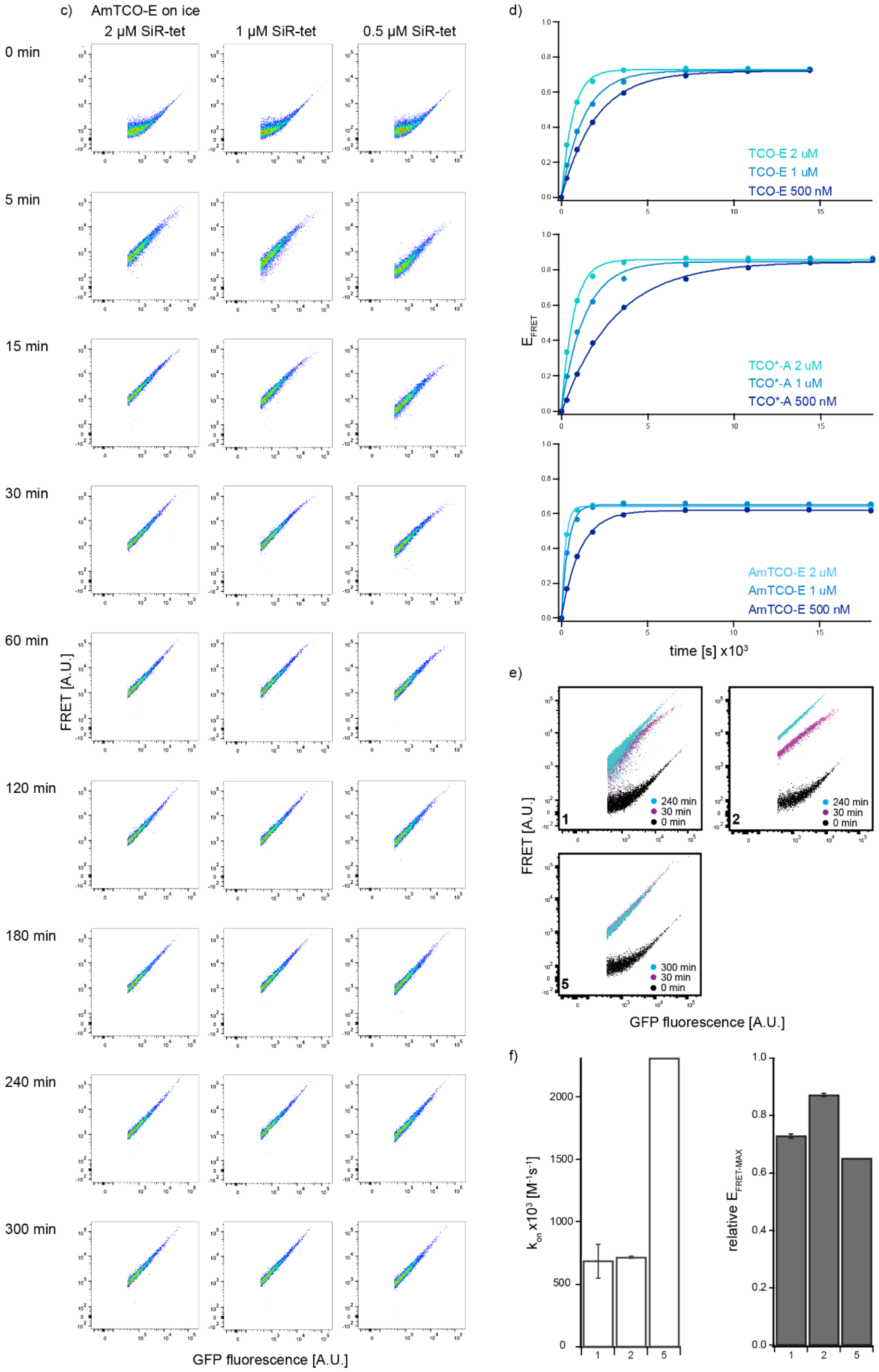
c)
AmTCO-E on ice
2 μM SiR-tet
1 μM SiR-tet
0.5 μM SiR-tet
0 min
5 min
15 min
30 min
60 min
120 min
180 min
240 min
300 min
FRET [A.U.]
GFP fluorescence [A.U.]
d)
TCO-E 2 uM
TCO-E 1 uM
TCO-E 500 nM
TCO*-A 2 uM
TCO*-A 1 uM
TCO*-A 500 nM
AmTCO-E 2 uM
AmTCO-E 1 uM
AmTCO-E 500 nM
E_{FRET}
time [s] x10³
e)
1
2
5
240 min
30 min
0 min
300 min
FRET [A.U.]
GFP fluorescence [A.U.]
f)
k_{on} x10³ [M⁻¹s⁻¹]
relative $E_{FRET\text{-}MAX}$

Figure S5. Results of the evolution of PylRS synthetase. a) After sfGFP test expression of the PylRS library in the 96-well plates, the best expressing clones were chosen for sequencing. The sequencing results are shown in the table in comparison to $PylRS^{AF}$. All PylRS variants show a Leucine residue at position 305 and a Tryptophan at position 417, which are the same for the $PylRS^{AF}$ synthetase. At the three other mutations sites (309, 348 and 405) are mutated to different amino acids. b) The best hits from the screening of the PylRS library after two rounds of selection were cloned into pcDNA3.1 plasmid harboring an U6tRNA expression gene, as well as a CMV promoter for the PylRS expression. The resulting plasmids were co-transfected with the reporter plasmid, pCI-FLAG-iRFP-25Helix-$EGFP^{Y39TAG}$-6His in HEK293T cells and the expression upon addition of TCO-E was analyzed after 24 hours using FFC. The signals were divided into transfected and untransfected cells, depending on their iRFP signal. The geometric mean (GM) of GFP and iRFP was extracted for the transfected cells signal and the ratio of GM GFP to GM iRFP was calculated. The bar graph shows the different expression ratios and indicates the highest ratio for the PylRS variant AF-A1.

a)

PylRS variant	PylRS mutation sites				
	305	309	348	405	417
AF	Leu	Leu	Cys	Ile	Trp
AF-A1	Leu	Met	Gly	Arg	Trp
AF-B11	Leu	Val	Gly	Asn	Trp
AF-C11	Leu	Cys	Gly	Ile	Trp
AF-G3	Leu	Val	Ala	Arg	Trp
AF-H12	Leu	Val	Gly	Arg	Trp

b)

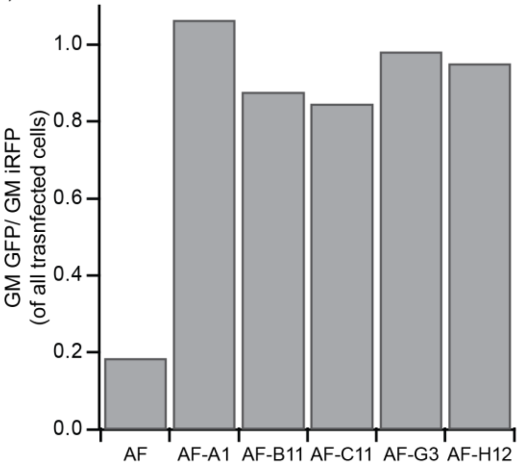

Figure S6. TCO-A *in cellulo* FRET kinetics and *in vitro* reactivity (sfGFP purified). a) Shown are the raw data of the *in cellulo* FRET FFC analysis for TCO-A as described in the supplementary methods. In brief, HEK293T cells expressing EGFP with TCO-A were labeled with the indicated concentration of SiR-tet and analyzed via FFC after indicated time points. The FFC plots show FRET signal [A.U.] over the GFP fluorescence signal [A.U.] for different time points (0-300 minutes). b) Kinetic plot containing the data from the three different dye concentrations used for the experiment, the highest concentration in cyan, the middle concentration in light blue and the lowest concentration in dark blue. The data points were fitted with an exponential fit using Igor Pro (WaveMetrics), to obtain k_{Obs} for each dye concentration. c) and d) show labeling experiments of sfGFP expressed in *E. coli* with the TCO-E (c) and TCO-A (d) respectively (upper panels) and the resulting E_{FRET} signal plotted over time for each compound (lower panels), explained more in details in the experimental details. Briefly, 100 nM of the corresponding sfGFP in a cuvette was mixed with 1 µM Cy5-tet, the mixture was excited at 450 nm and the emission measured from 470-700 nm every 5 seconds for 10 minutes.

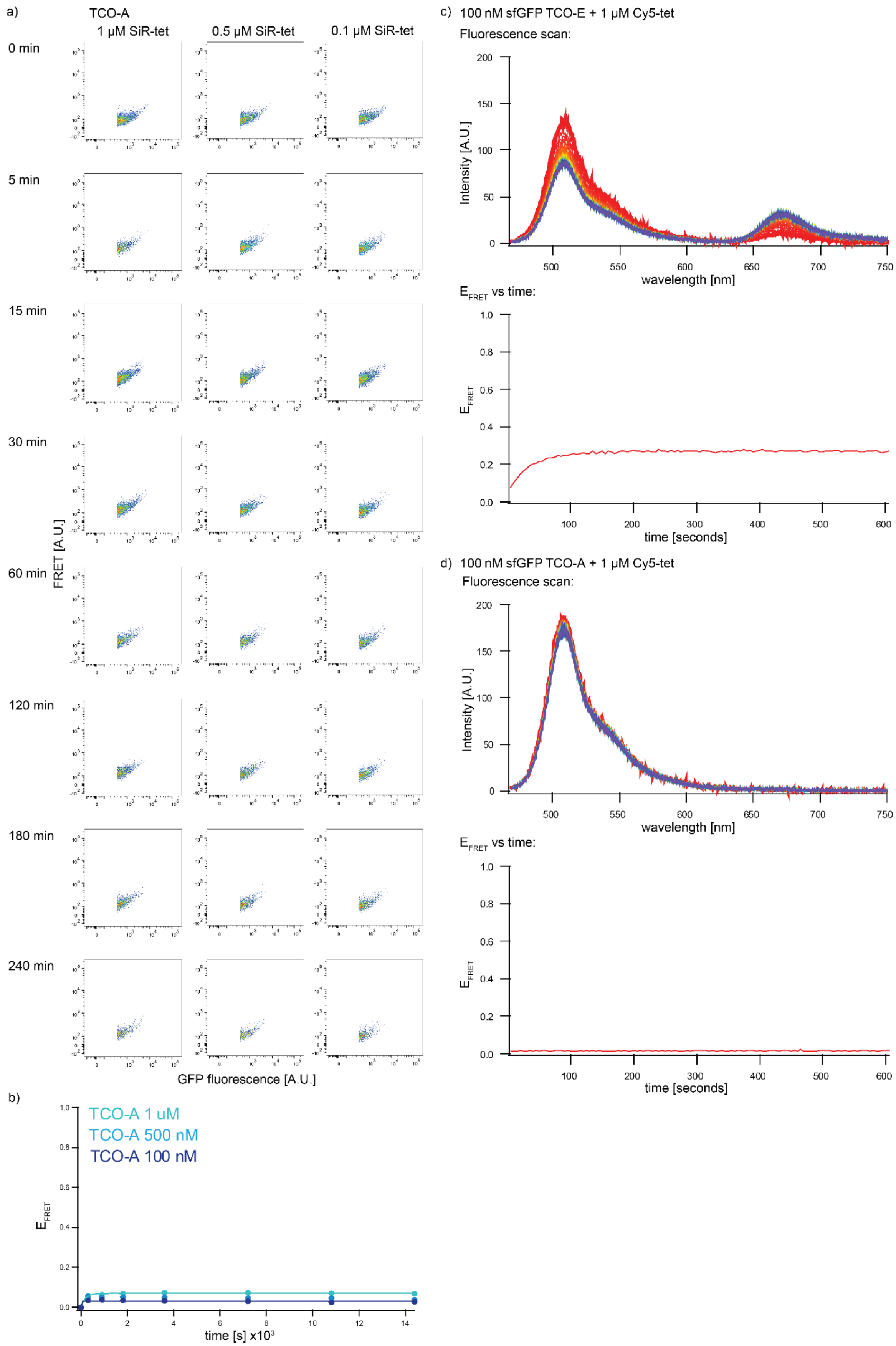
a)
TCO-A
1 μM SiR-tet
0.5 μM SiR-tet
0.1 μM SiR-tet
0 min
5 min
15 min
30 min
60 min
120 min
180 min
240 min
FRET [A.U.]
GFP fluorescence [A.U.]
b)
TCO-A 1 uM
TCO-A 500 nM
TCO-A 100 nM
E_FRET
time [s] x10³
c) 100 nM sfGFP TCO-E + 1 μM Cy5-tet
Fluorescence scan:
Intensity [A.U.]
wavelength [nm]
E_FRET vs time:
E_FRET
time [seconds]
d) 100 nM sfGFP TCO-A + 1 μM Cy5-tet
Fluorescence scan:
Intensity [A.U.]
wavelength [nm]
E_FRET vs time:
E_FRET
time [seconds]

Figure S7. *In vitro* FRET studies of recombinant $EGFP^{Y39ncAA}$. Shown are the *in vitro* FRET analysis of different $EGFP^{Y39ncAA}$ expressed in *E. coli* with the corresponding ncAA. After purification, $EGFP^{Y39ncAA}$ was labeled with the 1 µM of Cy5-tet and monitored over 10 minutes and 2 or 10 hours (as indicated) in a fluorescence spectrometer (excitation 450 nm, emission detection 470-750 nm). Fluorescence emission spectra are shown in the left column (color code indicates different time points, a spectrum was taken every 5 seconds in the case of the 10 min measurement, every 60 seconds for the 120 minutes measurements and every 5 minutes for the 10 hours measurement). The right column shows the E_{FRET} over time as calculated from the fluorescence spectra. $EGFP^{Y39TCO\text{-}E}$ only reaches an $E_{FRET\text{-}Max}$ of around 0.3, but therefore shows a fast kinetic. This is due to the trans- to cis-isomerization of TCO-E in LB medium (as Figure S9 illustrates). $EGFP^{Y39TCO*\text{-}A}$ reaches a high $E_{FRET\text{-}Max}$ of around 0.8, but shows elimination long term (2 hours). $EGFP^{Y39BCN}$ is not reacting with the dye and shows nearly the same $E_{FRET\text{-}MAX}$ as $EGFP^{Y39BOC}$, which is due to its degradation in LB medium (as Figure S9 illustrates). $EGFP^{Y39SCO}$ can reach a high $E_{FRET\text{-}MAX}$ of 0.7, but only if monitored over a long time (10 hours).

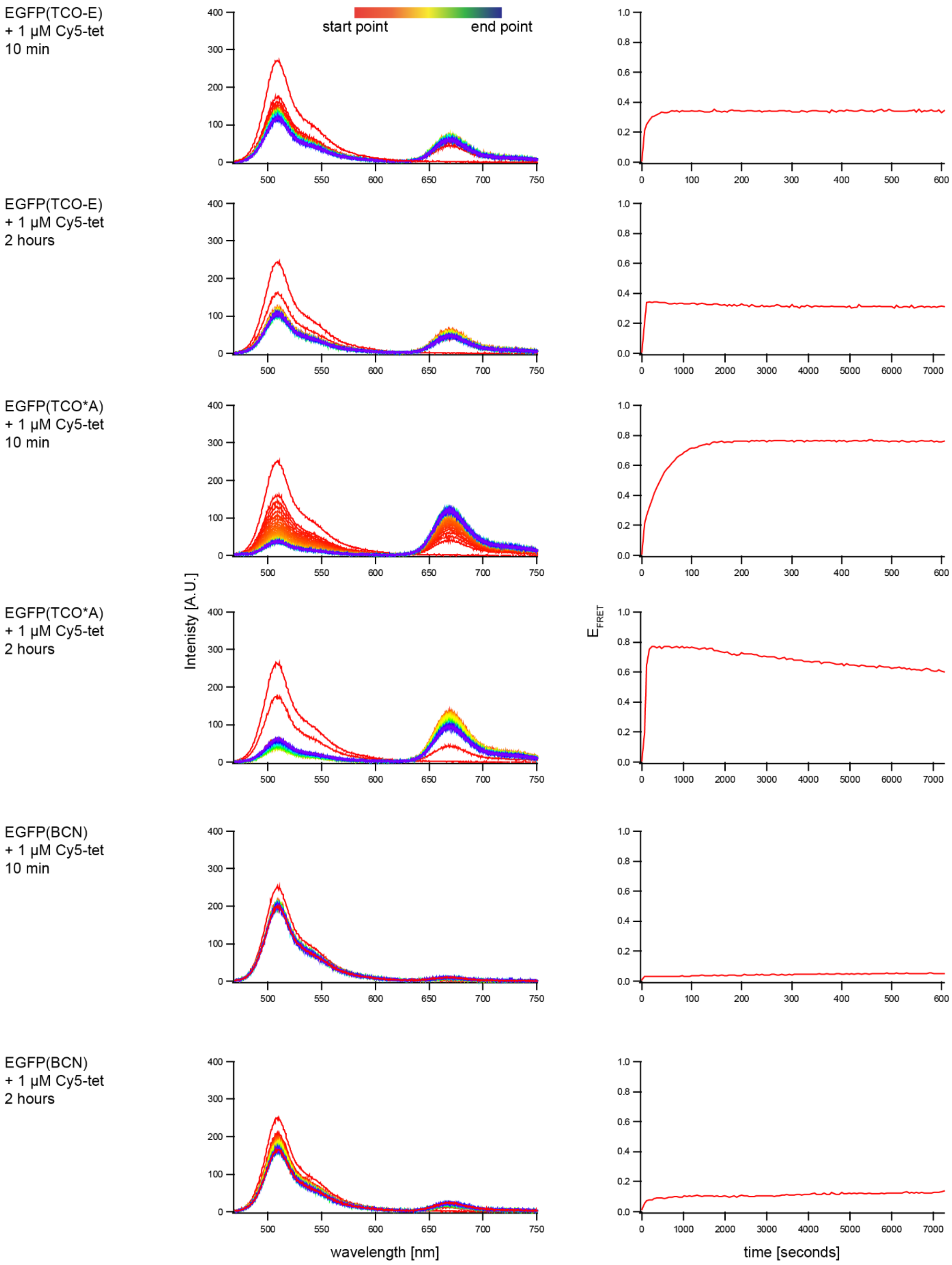

EGFP(TCO-E)
+ 1 µM Cy5-tet
10 min
EGFP(TCO-E)
+ 1 µM Cy5-tet
2 hours
EGFP(TCO*A)
+ 1 µM Cy5-tet
10 min
EGFP(TCO*A)
+ 1 µM Cy5-tet
2 hours
EGFP(BCN)
+ 1 µM Cy5-tet
10 min
EGFP(BCN)
+ 1 µM Cy5-tet
2 hours
start point
end point
Intenisty [A.U.]
E_FRET
wavelength [nm]
time [seconds]

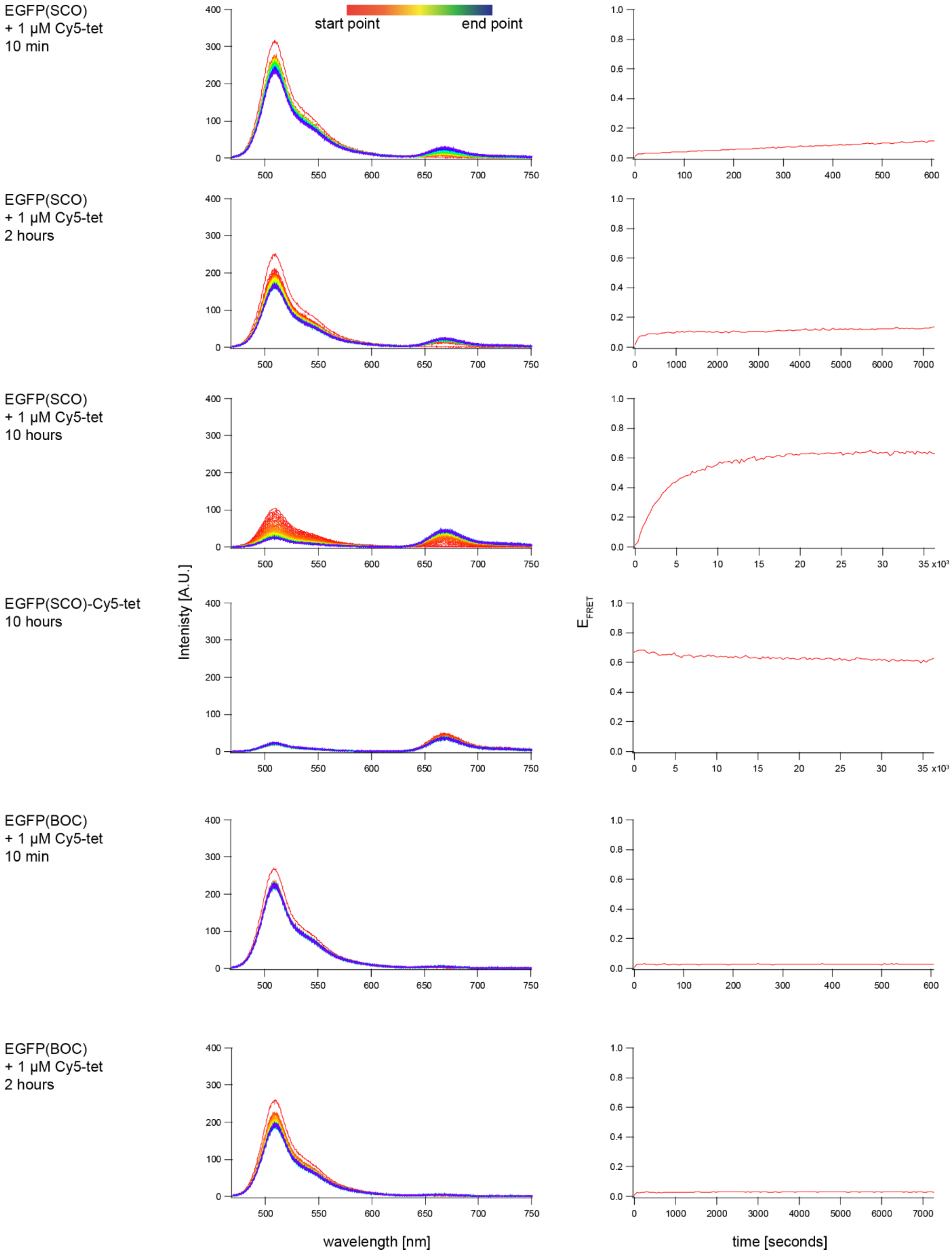
EGFP(SCO)
+ 1 µM Cy5-tet
10 min
EGFP(SCO)
+ 1 µM Cy5-tet
2 hours
EGFP(SCO)
+ 1 µM Cy5-tet
10 hours
EGFP(SCO)-Cy5-tet
10 hours
EGFP(BOC)
+ 1 µM Cy5-tet
10 min
EGFP(BOC)
+ 1 µM Cy5-tet
2 hours
start point
end point
Intenisty [A.U.]
E_{FRET}
wavelength [nm]
time [seconds]
x10³

Figure S8. LC-MS analysis of free ncAAs. The ncAAs TCO-A and TCO-E were first analyzed via LC-MS to identy where the elute from the column (top panels). Subesquently, 1 mM TCO-A or TCO-E in MeCN and were mixed with equal amounts of 1 mM dimethyl-tetrazine and analyzed after five minutes via LC-MS (bottom panels). The ncAAs elute as three peaks after reaction with the dimethyl-tetrazine, which all show the expected mass (lower 3 MS plots). The analysis shows fast and complete product formation for both ncAAs as no starting material can be detected after five minutes.

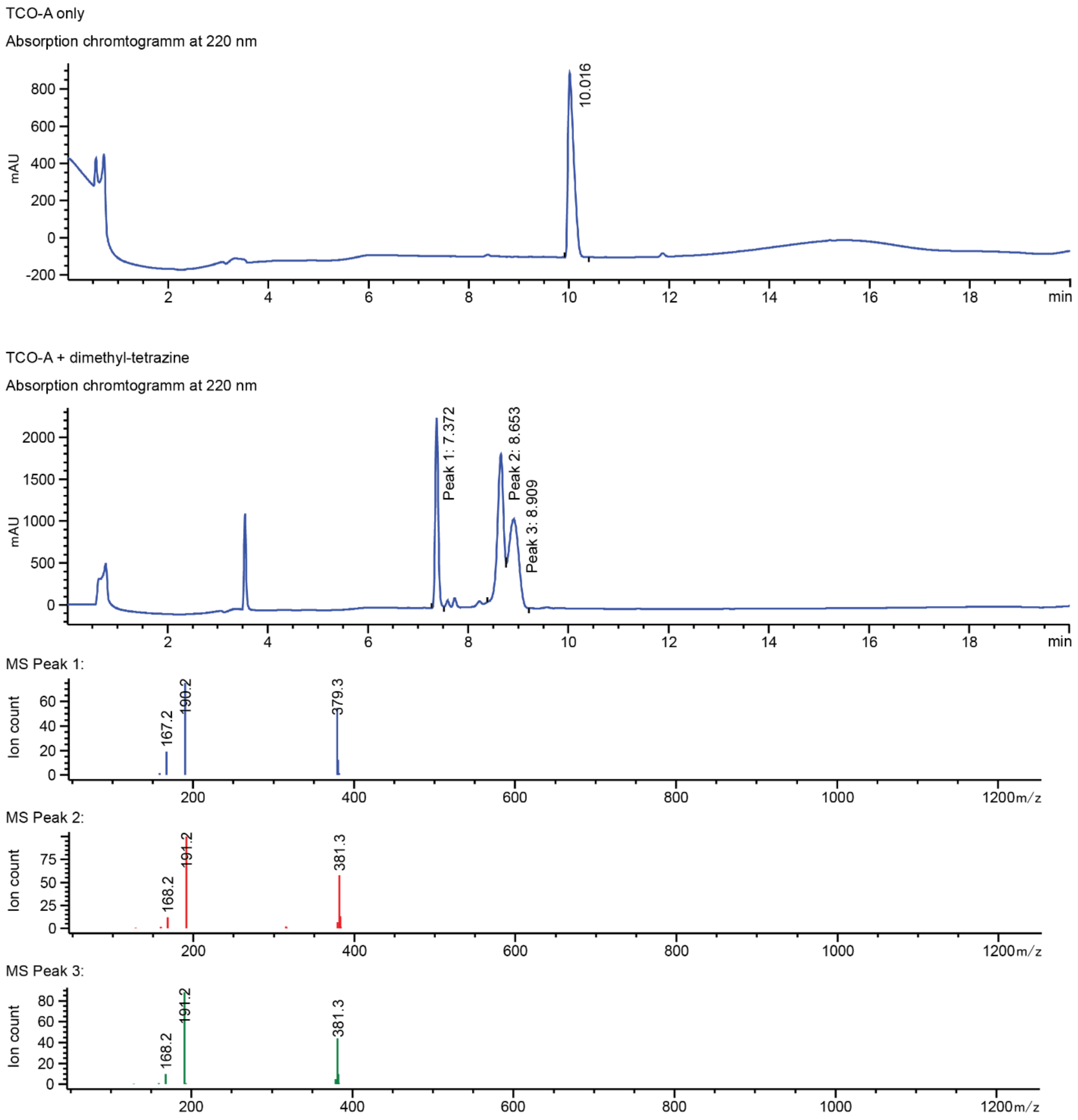

TCO-E only

Absorption chromtogramm at 220 nm

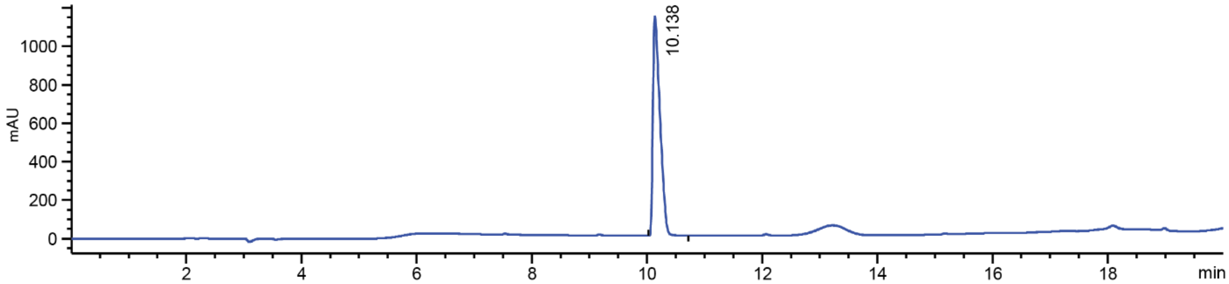

TCO-E + dimethyl-tetrazine

Absorption chromtogramm at 220 nm

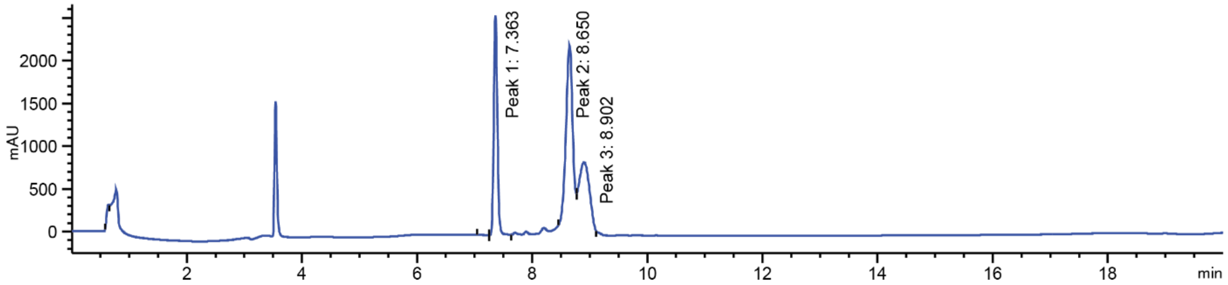

MS Peak 1:

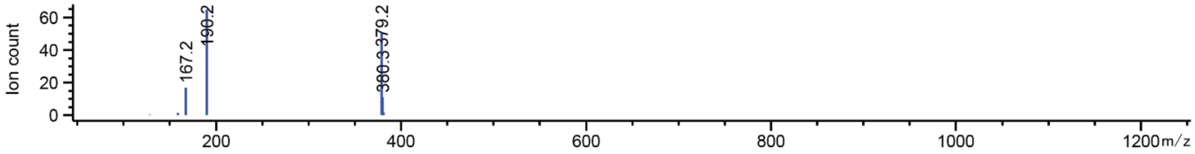

MS Peak 2:

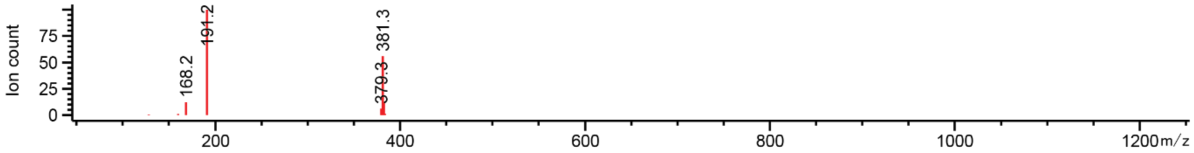

MS Peak 3:

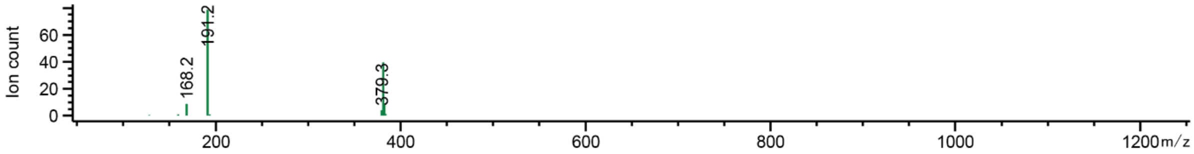

Figure S9. IEC-MS analysis of ncAAs in LB medium and *E. coli* cultures. The plots represent IEC-analysis at different time points for different ncAAs. For the analysis ncAAs were incubated either in LB medium or *E. coli* cultures and processed for analysis as described in the experimental details. ncAAs in LB medium were analyzed without further processing, while ncAAs in *E. coli* cultures were subjected to IEC-MS analysis after cell lysis. In the case of TCO-A, TCO-E and TCO*-A, the IEC-MS analysis show two peaks, one for the trans and one for the cis isomer of the corresponding ncAA. Each plot contains also a table, representing the percentage of trans- and cis-isomer at the specific time points. The control experiment for the molecular weight of 299 g/mol (corresponding to the molecular weight of TCO and TCO*) is shown directly after the chromatograms for TCO-A. For BCN it can be shown, that the overall quantity of the compound decreases over time. The control experiment for the molecular weight of 322 g/mol (corresponding to the molecular weight of BCN) is directly shown after the BCN data.

IEC analysis of different ncAAs in LB medium:

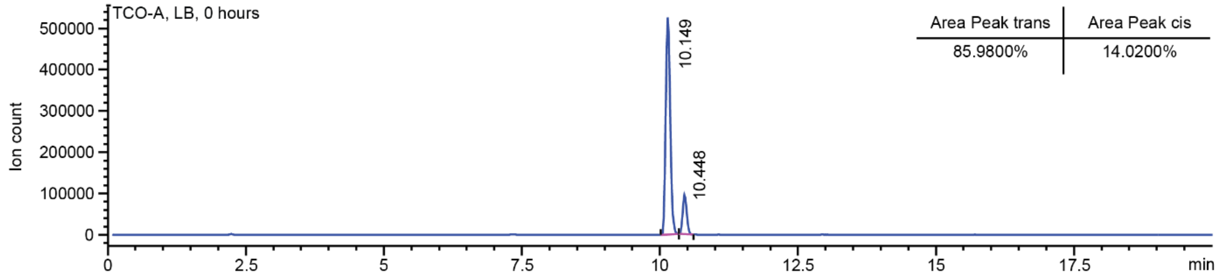

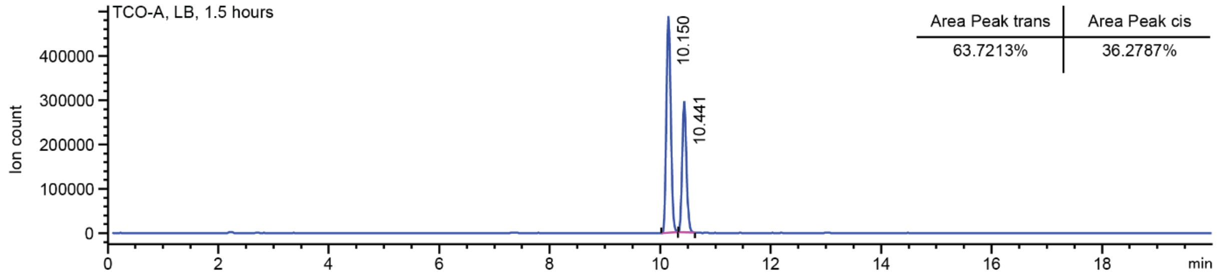

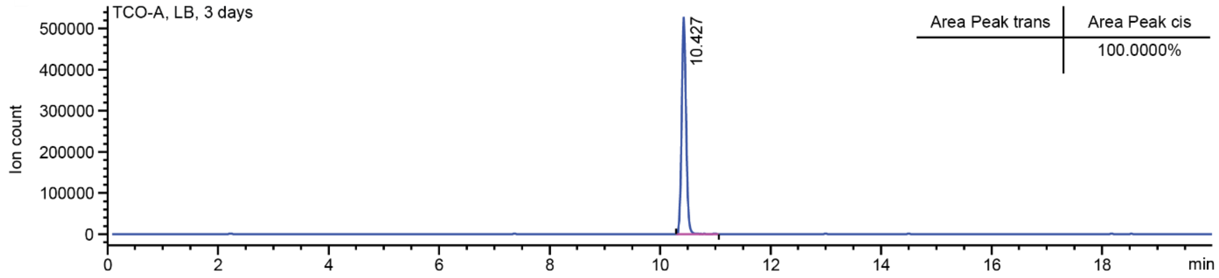

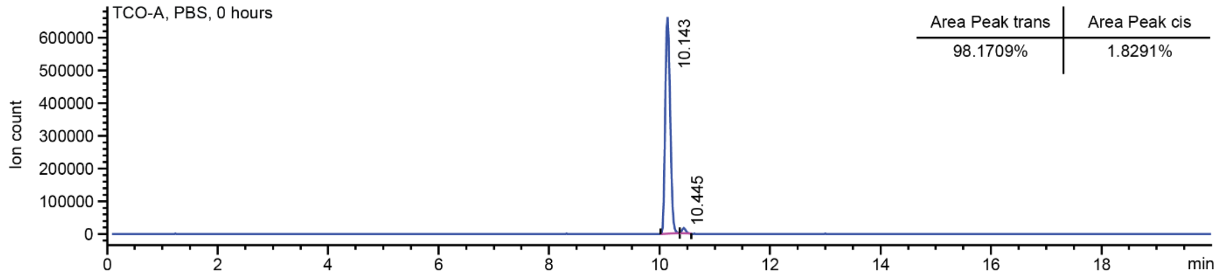

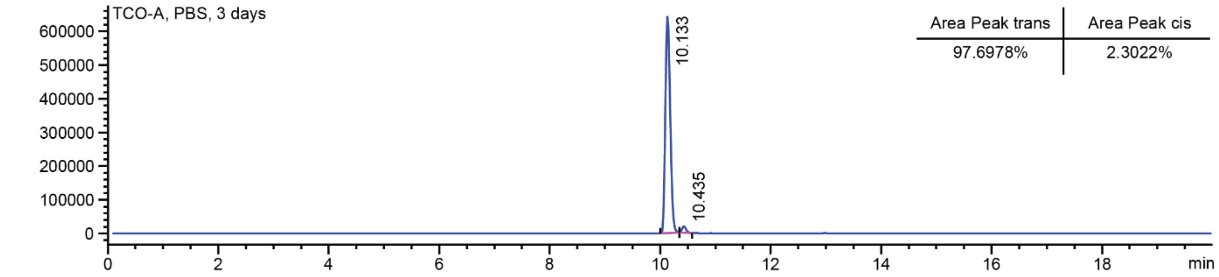

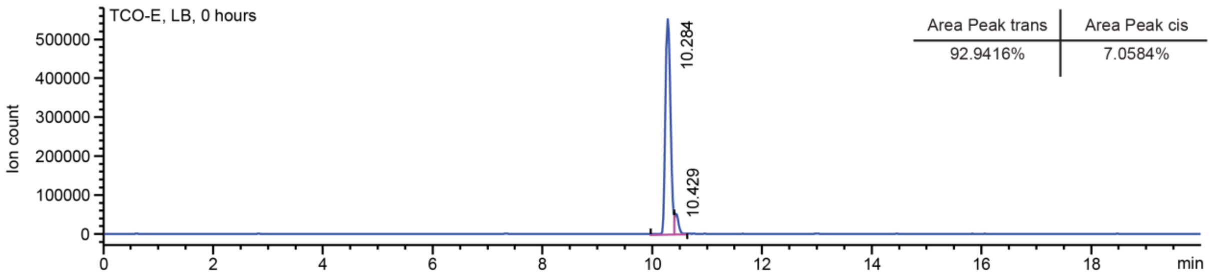
TCO-E, LB, 0 hours
Area Peak trans
Area Peak cis
92.9416%
7.0584%
10.284
10.429
Ion count
min

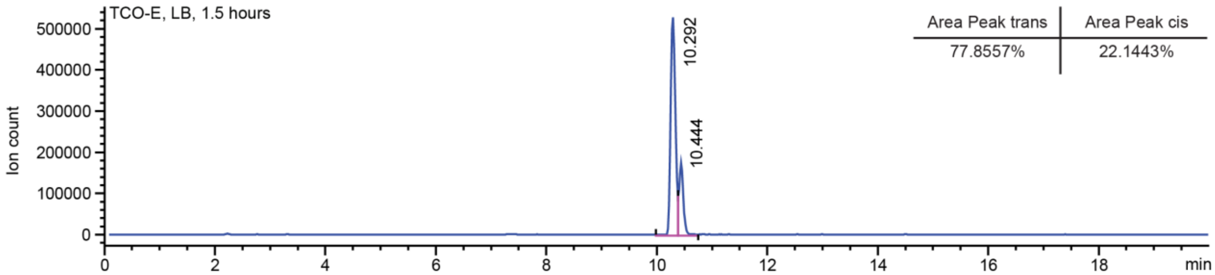
TCO-E, LB, 1.5 hours
Area Peak trans
Area Peak cis
77.8557%
22.1443%
10.292
10.444
Ion count
min

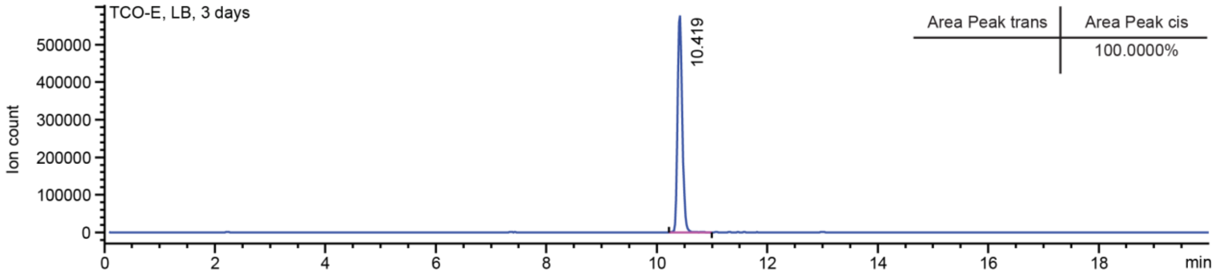
TCO-E, LB, 3 days
Area Peak trans
Area Peak cis
100.0000%
10.419
Ion count
min

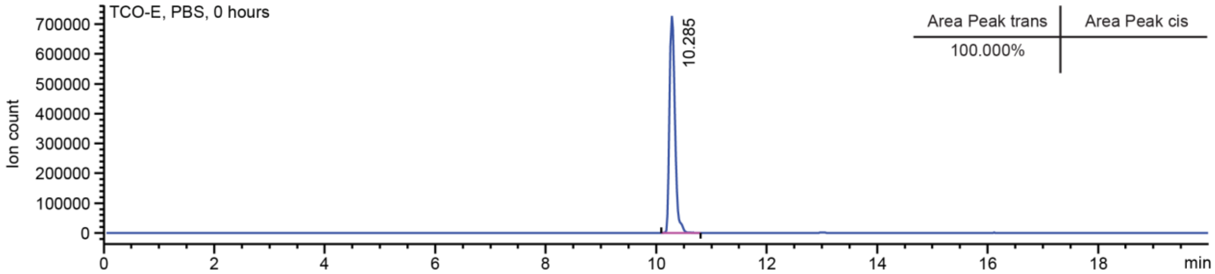
TCO-E, PBS, 0 hours
Area Peak trans
Area Peak cis
100.000%
10.285
Ion count
min

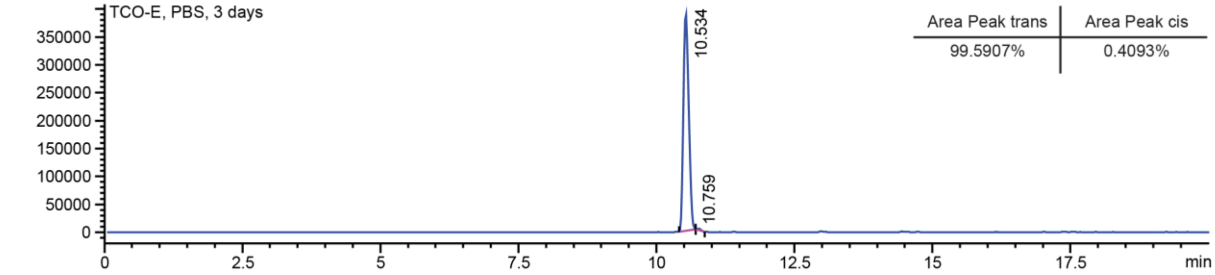
TCO-E, PBS, 3 days
Area Peak trans
Area Peak cis
99.5907%
0.4093%
10.534
10.759
min

IEC analysis of different ncAAs in *E. coli* cultures:

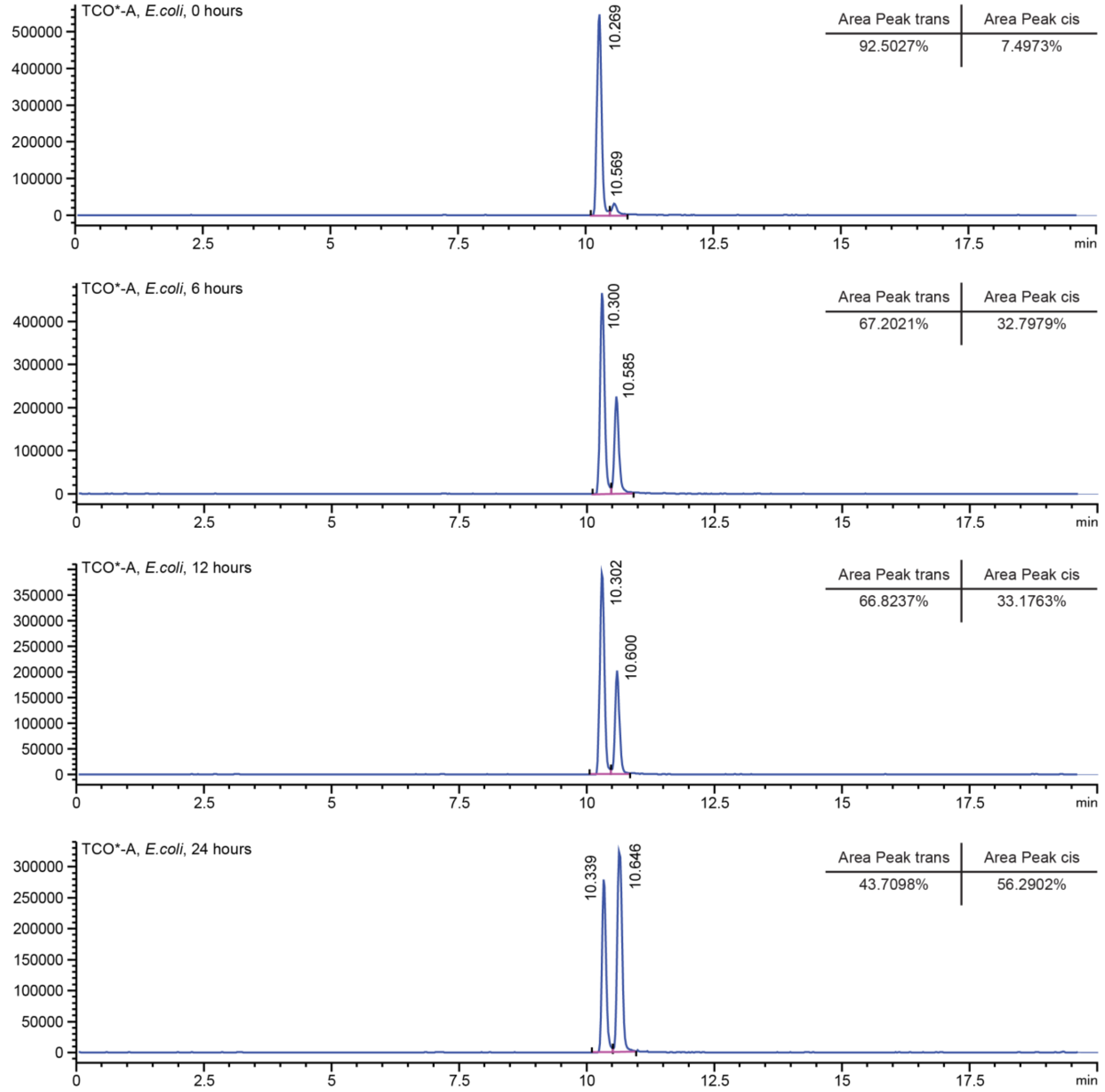

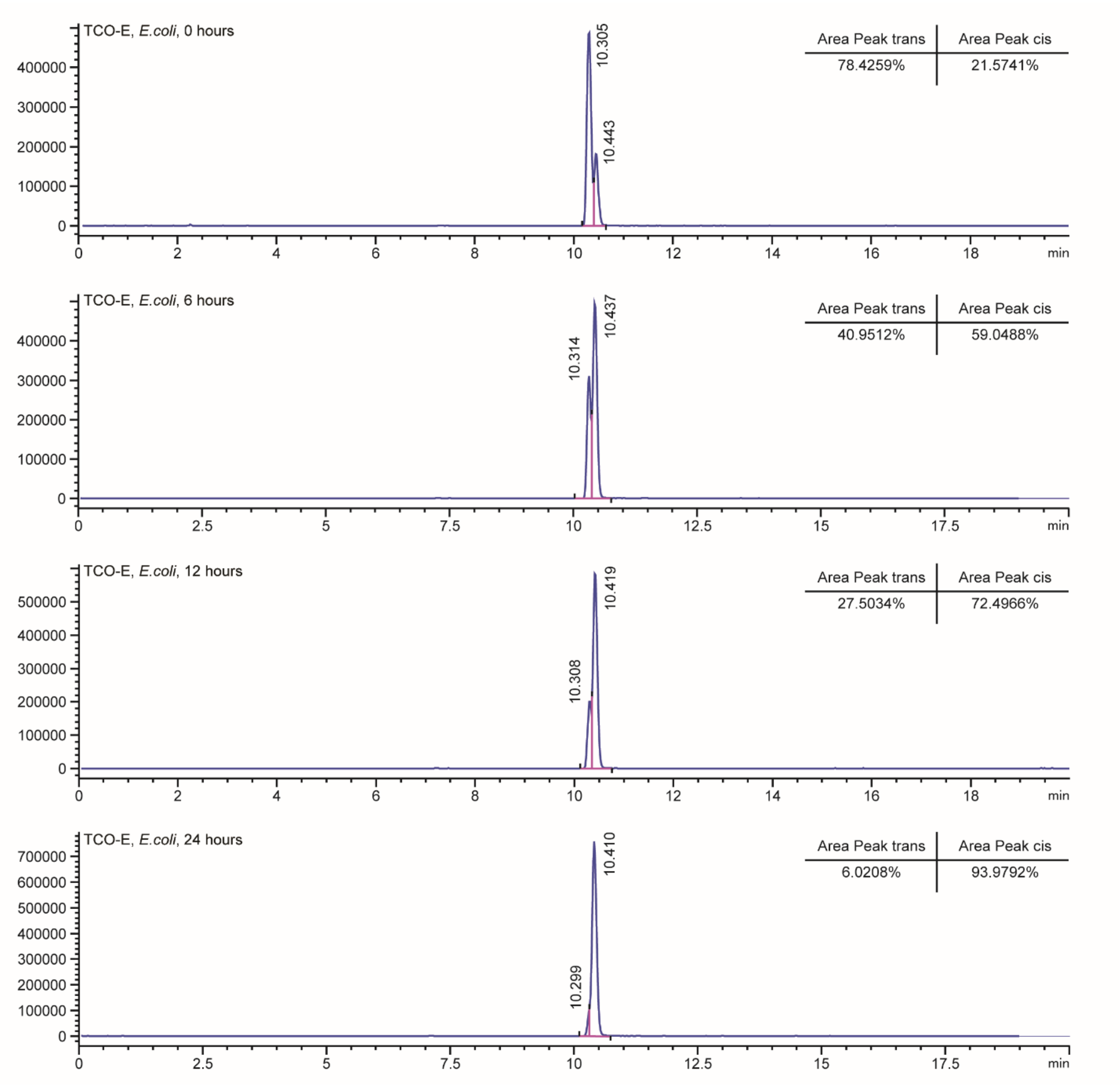
TCO-E, E.coli, 0 hours
Area Peak trans 78.4259%
Area Peak cis 21.5741%
10.305
10.443
TCO-E, E.coli, 6 hours
Area Peak trans 40.9512%
Area Peak cis 59.0488%
10.314
10.437
TCO-E, E.coli, 12 hours
Area Peak trans 27.5034%
Area Peak cis 72.4966%
10.308
10.419
TCO-E, E.coli, 24 hours
Area Peak trans 6.0208%
Area Peak cis 93.9792%
10.299
10.410
min

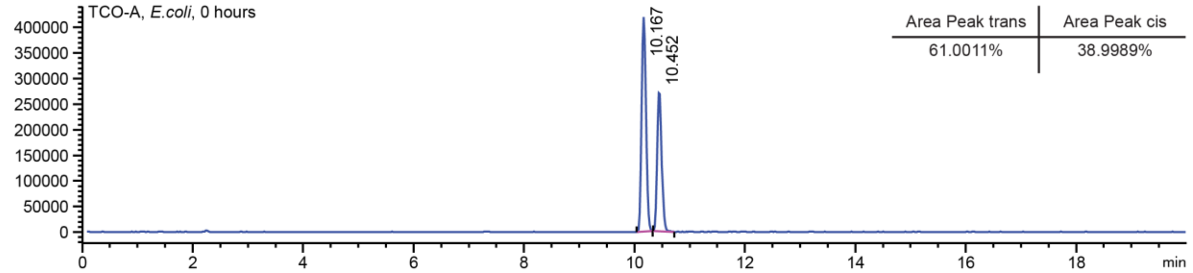
TCO-A, E.coli, 0 hours
10.167
10.452
Area Peak trans
Area Peak cis
61.0011%
38.9989%
min

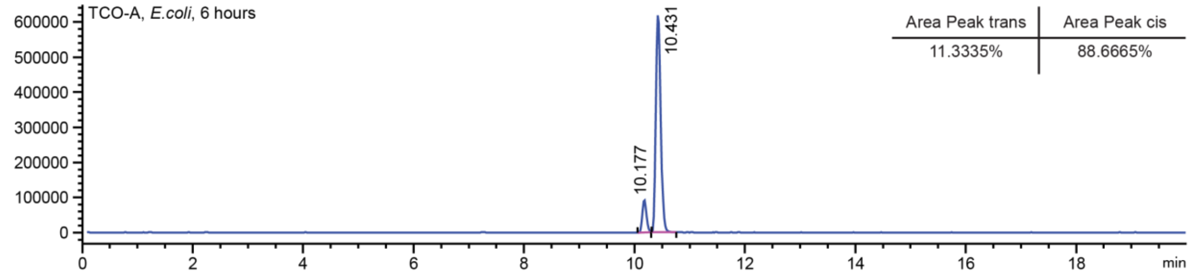
TCO-A, E.coli, 6 hours
10.431
10.177
Area Peak trans
Area Peak cis
11.3335%
88.6665%
min

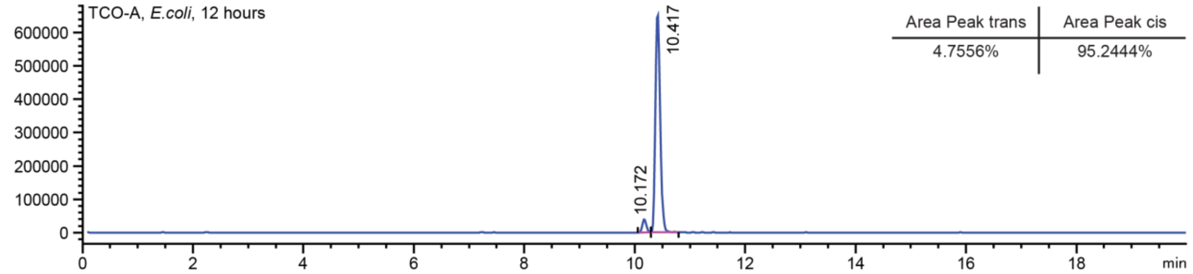
TCO-A, E.coli, 12 hours
10.417
10.172
Area Peak trans
Area Peak cis
4.7556%
95.2444%
min

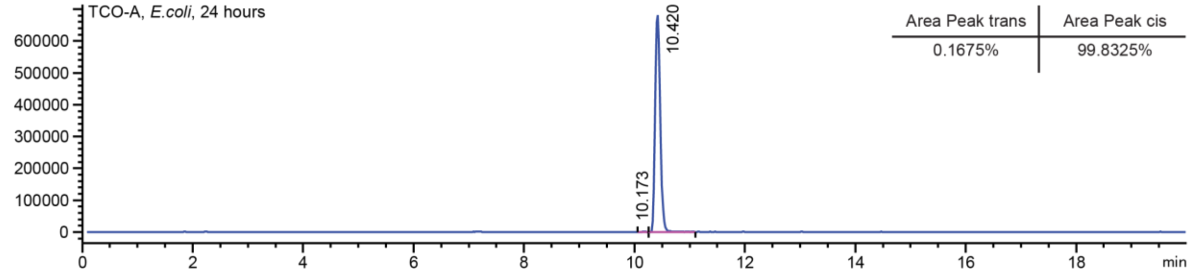
TCO-A, E.coli, 24 hours
10.420
10.173
Area Peak trans
Area Peak cis
0.1675%
99.8325%
min

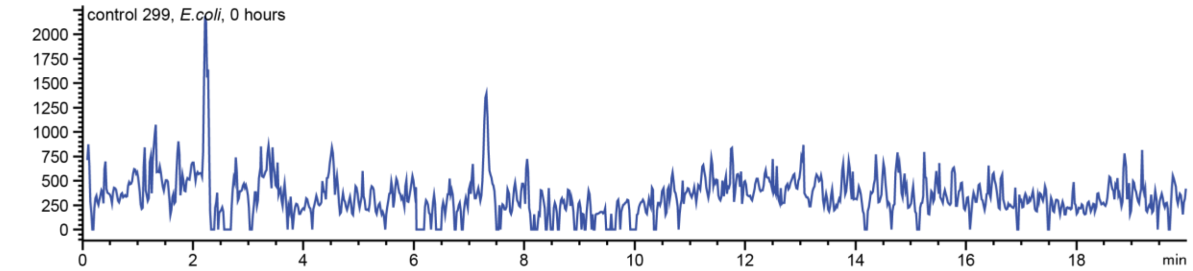
control 299, E.coli, 0 hours
min

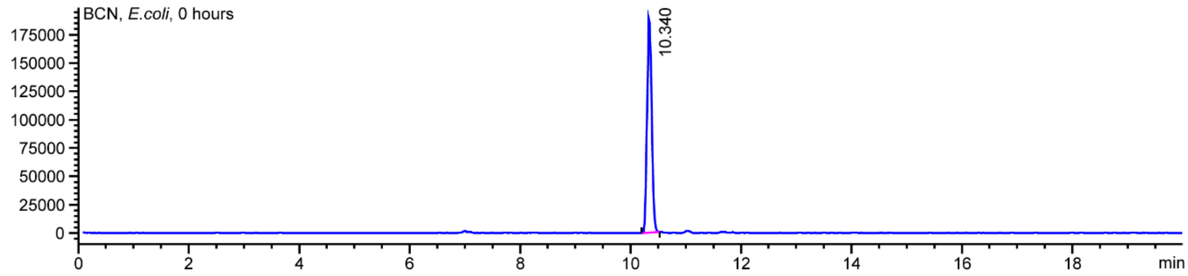
BCN, E.coli, 0 hours
10.340
175000
150000
125000
100000
75000
50000
25000
0
0 2 4 6 8 10 12 14 16 18 min

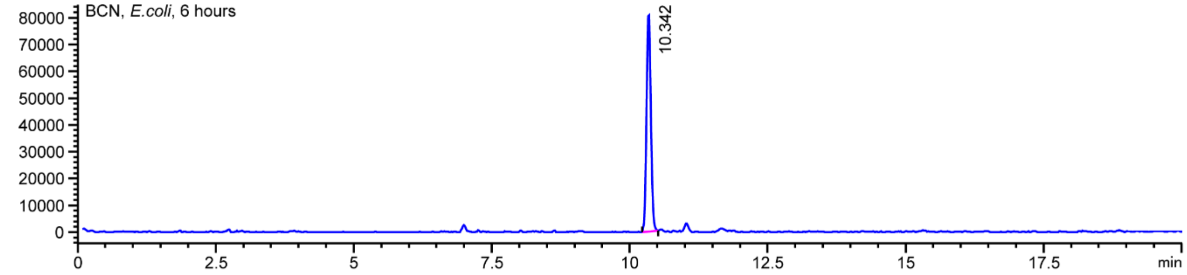
BCN, E.coli, 6 hours
10.342
80000
70000
60000
50000
40000
30000
20000
10000
0
0 2.5 5 7.5 10 12.5 15 17.5 min

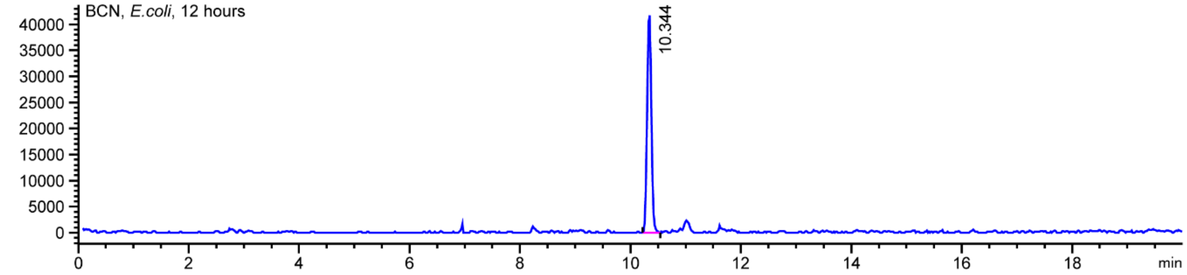
BCN, E.coli, 12 hours
10.344
40000
35000
30000
25000
20000
15000
10000
5000
0
0 2 4 6 8 10 12 14 16 18 min

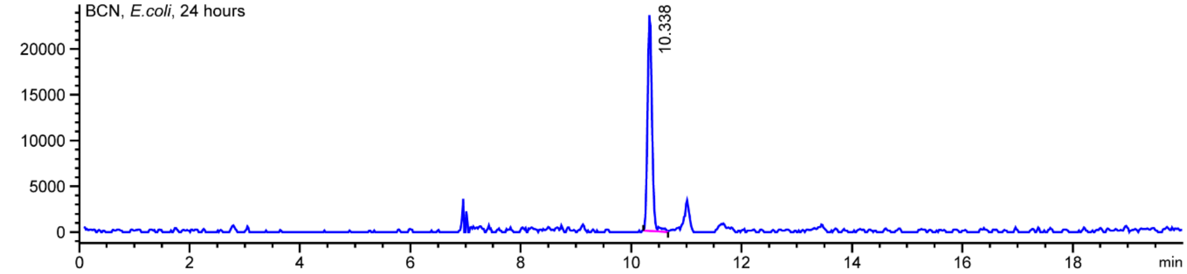
BCN, E.coli, 24 hours
10.338
20000
15000
10000
5000
0
0 2 4 6 8 10 12 14 16 18 min

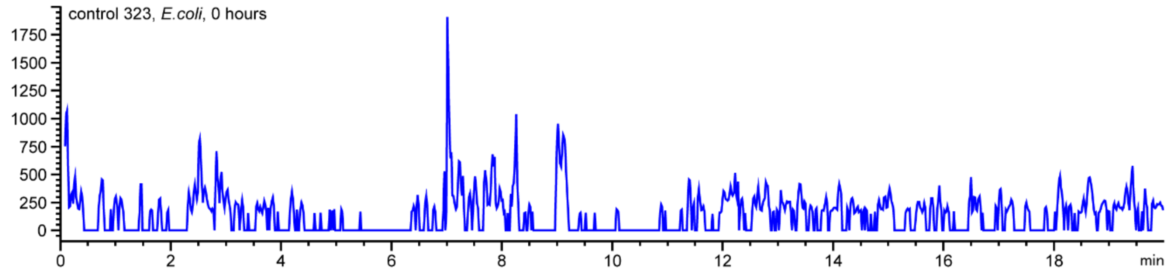
control 323, E.coli, 0 hours
1750
1500
1250
1000
750
500
250
0
0 2 4 6 8 10 12 14 16 18 min

Supporting references

[1] I. Nikic, G. Estrada Girona, J. H. Kang, G. Paci, S. Mikhaleva, C. Koehler, N. V. Shymanska, C. Ventura Santos, D. Spitz, E. A. Lemke, *Angew Chem Int Ed Engl* **2016**, *55*, 16172-16176.

[2] R. B. Cooley, J. L. Feldman, C. M. Driggers, T. A. Bundy, A. L. Stokes, P. A. Karplus, R. A. Mehl, *Biochemistry* **2014**, *53*, 1916-1924.

[3] J. J. Porter, H. S. Jang, E. M. Van Fossen, D. P. Nguyen, T. S. Willi, R. B. Cooley, R. A. Mehl, *ACS Chem Biol* **2019**, *14*, 1328-1336.

[4] I. Nikic, J. H. Kang, G. E. Girona, I. V. Aramburu, E. A. Lemke, *Nat Protoc* **2015**, *10*, 780-791.

[5] G. Lukinavicius, K. Umezawa, N. Olivier, A. Honigmann, G. Yang, T. Plass, V. Mueller, L. Reymond, I. R. Correa, Jr., Z. G. Luo, C. Schultz, E. A. Lemke, P. Heppenstall, C. Eggeling, S. Manley, K. Johnsson, *Nat Chem* **2013**, *5*, 132-139.

[6] M. Royzen, G. P. Yap, J. M. Fox, *J Am Chem Soc* **2008**, *130*, 3760-3761.

[7] D. Svatunek, C. Denk, V. Rosecker, B. Sohr, C. Hametner, G. Allmaier, J. Frohlich, H. Mikula, *Monatsh Chem* **2016**, *147*, 579-585.

[8] J. E. Hoffmann, T. Plass, I. Nikic, I. V. Aramburu, C. Koehler, H. Gillandt, E. A. Lemke, C. Schultz, *Chemistry* **2015**, *21*, 12266-12270.

[9] M. Kardos, L. Kiss, F. Fülöp, *Asian Journal of Organic Chemistry* **2015**, *4*, 1155-1159.

[10] B. K. Shull, T. Sakai, J. B. Nichols, M. Koreeda, *J Org Chem* **1997**, *62*, 8294-8303.

[11] C. Echalier, A. Rutkowska, A. Kojic, D. W. Thomson, L. J. Edwards, B. S. J. McKay, M. Mulbaier, C. Schultz, G. Bergamini, *Chem Commun (Camb)* **2021**. DOI: 10.1039/D0CC06709A
